# New Antibacterial Chloro-Containing Polyketides from the Alga-Derived Fungus *Asteromyces cruciatus* KMM 4696

**DOI:** 10.3390/jof8050454

**Published:** 2022-04-27

**Authors:** Olesya I. Zhuravleva, Galina K. Oleinikova, Alexandr S. Antonov, Natalia N. Kirichuk, Dmitry N. Pelageev, Anton B. Rasin, Alexander S. Menshov, Roman S. Popov, Natalya Yu. Kim, Ekaterina A. Chingizova, Artur R. Chingizov, Olga O. Volchkova, Gunhild von Amsberg, Sergey A. Dyshlovoy, Ekaterina A. Yurchenko, Irina V. Guzhova, Anton N. Yurchenko

**Affiliations:** 1 G.B. Elyakov Pacific Institute of Bioorganic Chemistry, Far Eastern Branch of the Russian Academy of Sciences, Prospect 100-Letiya Vladivostoka, 159, 690022 Vladivostok, Russia; oleingk@mail.ru (G.K.O.); antonov_as@piboc.dvo.ru (A.S.A.); sheflera@bk.ru (N.N.K.); pelageev@mail.ru (D.N.P.); abrus__54@mail.ru (A.B.R.); menshov90@piboc.dvo.ru (A.S.M.); popov_rs@piboc.dvo.ru (R.S.P.); kim_ny@piboc.dvo.ru (N.Y.K.); martyyas@mail.ru (E.A.C.); chingizov_ar@piboc.dvo.ru (A.R.C.); eyurch@piboc.dvo.ru (E.A.Y.); yurchenkoan@piboc.dvo.ru (A.N.Y.); 2 Institute of High Technologies and Advanced Materials, Far Eastern Federal University, 10 Ajax Bay, Russky Island, 690922 Vladivostok, Russia; olavol1309@gmail.com (O.O.V.); s.dyshlovoy@uke.de (S.A.D.); 3 Laboratory of Experimental Oncology, Department of Oncology, Hematology and Bone Marrow Transplantation with Section Pneumology, Hubertus Wald-Tumorzentrum, University Medical Center Hamburg-Eppendorf, 20246 Hamburg, Germany; g.von-amsberg@uke.de; 4 Martini-Klinik Prostate Cancer Center, University Hospital Hamburg-Eppendorf, 20246 Hamburg, Germany; 5 Institute of Cytology Russian Academy of Sciences, Tikhoretskiy Ave. 4, 194064 St. Petersburg, Russia; ina.guzhova@incras.ru

**Keywords:** *Asteromyces cruciatus*, marine fungi, secondary metabolites, polyketides, sortase A, chlore-containing metabolites, *Staphylococcus aureus*, antibacterial activity, biofilm formation

## Abstract

Six new polyketides acrucipentyns A–F (**1**–**6**) were isolated from the alga-derived fungus *Asteromyces cruciatus* KMM 4696. Their structures were established based on spectroscopic methods. The absolute configurations of acrucipentyn A was assigned by the modified Mosher’s method and ROESY data analysis. Acrucipentyns A–E were identified to be the very first examples of chlorine-containing asperpentyn-like compounds. The cytotoxic and antimicrobial activities of the isolated compounds were examined. Acrucipentyns A–F were found as antimicrobial agents, which inhibited sortase A enzyme activity, bacterial growth and biofilm formation of *Staphylococcus aureus* and decreased LDH release from human keratinocytes HaCaT in *S. aureus* skin infection in an in vitro model.

## 1. Introduction

Microfilamentous fungi isolated from a variety of marine environments can be divided into facultative and obligate groups [1]. Facultative marine fungi are those found in both marine and terrestrial sources. The metabolism of such fungi species adapts to marine environment conditions and produces secondary metabolites, which are unusual for these species. For example, the well-known and widespread fungus *Penicillium chrysogenum,* isolated from marine sediments, was reported to be a producer of the unique dimeric nitrophenyl *trans*-epoxyamides, chrysamides A–C [2]. Obligate marine fungi are exclusively found in marine sources and have never been isolated from terrestrial samples. The metabolism of obligate marine fungi is more dramatically altered by the saline stress and other factors. The biosynthesis of previously undescribed chemical structures is the consequence [3].

Obligate marine fungus *Asteromyces cruciatus* C. Moreau et Moreau ex Hennebert is a widespread species, which can be found in the tropical and temperate zones of the Pacific, Atlantic and Indian oceans. This species was initially discovered in the sand sample of the coastal area and was validly described in 1961. *A. cruciatus* can be found drifting or washed up on the sore wood, algae, in sediment bottom samples. Currently, this species is still poorly studied, and its position in the fungal classification tree remains uncertain [4].

Nevertheless, a few chemical studies have shown that *A. cruciatus* is a promising source of new secondary metabolites. Thus, diketopiperazine gliovictin was the first compound isolated from *A. cruciatus* [5]. The fungus *A. cruciatus* 763 yielded the new pentapeptide lajollamide A, which exhibited a weak antibacterial activity, along with several known sulfur-containing diketopiperazines [6]. Two new polyketides, primarolides A and B, were isolated from an A. cruciatus culture treated with suberoylanilide hydroxamic acid and high concentrations of NaCl [7].

Being a part of the microbial community, both facultative and obligate marine fungi produce various bioactive secondary metabolites, which help them to interact and fight with other species. By 2021, about 300 small molecules possessing a potent antimicrobial activity and isolated from various marine fungi had been reported [8,9]. Marine fungal secondary metabolites exhibit their antibacterial effects by directly inhibiting bacterial growth, as well as by decreasing virulence or biofilms formation [10]. A membrane-associated enzyme, sortase A, is responsible for the covalent attachment of many virulent Gram-positive bacteria, including *Staphylococcus aureus*, to the mammalian cell wall [11,12]. Thus, the sortase A enzyme is an attractive target for new drugs against virulent and antibiotic-resistant Gram-positive bacteria, which are known to be one of the main causes of infectious disease worldwide [13]. The compounds capable of sortase A inhibition and lacking, at the same time, cytotoxicity to mammalian cells are of particular interest, because they can inhibite bacterial biofilm formation and decrease the virulence and toxicity of bacteria.

In the current research, as a part of our continuing efforts to search for new antibacterial metabolites in marine fungi [14,15], we isolated new isoprenylated cyclohexanols acrucipentyns A–F (**1**–**6**) from a culture of *Asteromyces cruciatus* KMM 4696 associated with brown alga *Sargassum pallidum* (Vostok Bay, the Sea of Japan). The effect of acrucipentyns on enzymatic activity of sortase A from *Staphylococcus aureus*, growth and biofilm formation of *S. aureus,* as well as toxicity of acrucipentyns to various mammalian (human) cells, were tested. Finally, the effect of isolated compounds on human keratinocytes HaCaT co-cultured with *S. aureus* was investigated.

## 2. Materials and Methods

### 2.1. General Experimental Procedures

Optical rotations were measured on a Perkin-Elmer 343 polarimeter (Perkin Elmer, Waltham, MA, USA). UV spectra were recorded on a Shimadzu UV-1601PC spectrometer (Shimadzu Corporation, Kyoto, Japan) in methanol. CD spectra were measured with a Chirascan-Plus CD spectrometer (Leatherhead, UK) in methanol. NMR spectra were recorded in CDCl_3_, acetone-*d_6_* and DMSO-*d_6_*, on a Bruker DPX-300 (Bruker BioSpin GmbH, Rheinstetten, Germany), a Bruker Avance III-500 (Bruker BioSpin GmbH, Rheinstetten, Germany) and a Bruker Avance III-700 (Bruker BioSpin GmbH, Rheinstetten, Germany) spectrometer, using TMS as an internal standard. HRESIMS spectra were measured on a Maxis impact mass spectrometer (Bruker Daltonics GmbH, Rheinstetten, Germany). Microscopic examination and photography of fungal cultures were performed with Olympus CX41 microscope equipped with an Olympus SC30 digital camera. Detailed examination of ornamentation of the fungal conidia was performed using scanning electron microscopy (SEM) EVO 40.

Low-pressure liquid column chromatography was performed using silica gel (60/100 μm, Imid Ltd., Krasnodar, Russia) and Gel ODS-A (12 nm, S—75 um, YMC Co., Ishikawa, Japan). Plates precoated with silica gel (5–17 μm, 4.5 cm × 6.0 cm, Imid Ltd., Russia) and silica gel 60 RP-18 F_254_S (20 cm × 20 cm, Merck KGaA, Darmstadt, Germany) were used for thin-layer chromatography. Preparative HPLC was carried out on an Agilent 1100 chromatograph (Agilent Technologies, Santa Clara, CA, USA) with an Agilent 1100 refractometer (Agilent Technologies, Santa Clara, CA, USA) and a Shimadzu LC-20 chromatograph (Shimadzu USA Manufacturing, Canby, OR, USA) with a Shimadzu RID-20A refractometer (Shimadzu Corporation, Kyoto, Japan) using YMC ODS-AM (YMC Co., Ishikawa, Japan) (5 µm, 10 mm × 250 mm), YMC ODS-AM (YMC Co., Ishikawa, Japan) (5 µm, 4.6 mm × 250 mm) and Hydro-RP (Phenomenex, Torrance, CA, USA) (4 μm, 250 mm × 10 mm) columns.

### 2.2. Fungal Strain

The brown algae samples were collected in Vostok Bay (Sea of Japan) in sterile plastic bags. Before use, they were stored in a freezer at −18 °C. Isolation of fungi from algae samples was carried out by the plate method using Tubaki agar medium. The fungus was isolated into a pure culture by transferring the inoculum from a Petri dish onto a slant wort agar, where it was further stored. Microscopic examination of the strain was performed using Olympus CX41.

For DNA isolation, a fungus culture grown at 25 °C for 7 days was used. Isolation of genomic DNA was carried out using a commercial DNA kit (DNA-Technology Ltd., Moscow, Russia) in accordance with the protocol. Amplification and sequencing of the ITS genes were performed using ITS1 and ITS4 gene-specific primers [16]. The obtained sequence was compared with the GenBank sequence dataset and registered under accession number OL477331.

### 2.3. Cultivation of Fungus

The fungus was cultured at 22 °C for three weeks in 60 × 500 mL Erlenmeyer flasks, each containing rice (20.0 g), yeast extract (20.0 mg), KH_2_PO_4_ (10 mg) and natural seawater from the Marine Experimental Station of PIBOC, Troitsa (Trinity) Bay, Sea of Japan (40 mL).

### 2.4. Extraction and Isolation

At the end of the incubation period, the mycelia and medium were homogenized and extracted with EtOAc (1 L). The obtained extract was concentrated to dryness. The residue (17.5 g) was dissolved in H_2_O–EtOH (4:1) (100 mL) and extracted successively with *n*-hexane (0.2 L × 3), EtOAc (0.2 L × 3) and BuOH (0.2 L × 3). After evaporation of the ethyl acetate layer, the residual material (5.5 g) was subjected to column chromatography on silica gel, which was eluted with a gradient of *n*-hexane in ethyl acetate (100:1, 95:5, 90:10, 80:20, 70:30, 50:50). Fractions of 250 mL were collected and combined on the basis of TLC (silica gel, toluene–isopropanol 6:1 and 3:1, *v/v*).

The fractions of *n*-hexane-EtOAc (95:5, 80 mg) and *n*-hexane-EtOAc (90:10, 200 mg) were separated on a Gel ODS-A column (1.5 cm × 8 cm), which was eluted by a step gradient from 40% to 80% CH_3_OH in H_2_O (total volume 1 L), to afford subfractions I and II. Subfraction I (40% CH_3_OH, 146 mg) was purified by RP HPLC on a YMC ODS-AM column eluted with CH_3_OH-H_2_O (90:10) and then with CH_3_OH-H_2_O (60:40) to yield **2** (1.8 mg) and **4** (6.0 mg). Subfraction II (60% CH_3_OH, 110 mg) was purified by RP HPLC on a YMC ODS-AM column eluted with CH_3_OH-H_2_O (80:20) and then with CH_3_OH-H_2_O (55:45) to yield **1** (7 mg).

The *n*-hexane-EtOAc (80:20, 470 mg) fraction was separated on a Gel ODS-A column (1.5 cm × 8 cm), which was eluted with a step gradient from 40% to 80% CH_3_OH in H_2_O (total volume 1 L) to give subfraction III. Subfraction III (40% CH_3_OH, 250 mg) was separated by RP HPLC on a YMC ODS-AM column eluting with CH_3_OH-H_2_O (90:10) and then with CH_3_OH-H_2_O (60:40) to yield **6** (58 mg).

After evaporation of the BuOH layer, the residual material (0.98 g) was passed through a silica column (3 cm × 14 cm), which was separated in the same way as the ethyl acetate extract.

The *n*-hexane-EtOAc (50:50, 252 mg) fraction was purified by RP HPLC on a YMC ODS-A column eluted with CH_3_OH-H_2_O (45:1055) and then with CH_3_CN-H_2_O (25:75) to yield **3** (3.9 mg) and **5** (5.2 mg).

### 2.5. Spectral Data

Acrucipentyn A (**1**): amorphous solids; [α]_D_^20^ – 36.0 (*c* 0.09 CH_3_OH); CD (*c* 9.6 × 10^−4^, CH_3_OH), λ_max_ (∆ε) 210 (−0.97), 266 (−0.14) nm, see Supplementary Figure S1; UV (CH_3_OH) *λ*_max_ (log *ε*) 271 (2.54), 256 (2.50) and 224 (3.77) nm, see Supplementary Figure S7; ^1^H and ^13^C NMR data, see Table 1 and Table 2, Supplementary Figures S13–S22; HRESIMS *m*/*z* 229.0625 [M – H]^–^ (calcd. for C_11_H_14_ClO_3_, 229.0637, Δ5.0 ppm), 253.0599 [M + Na]^+^ (calcd. for C_11_H_15_ClO_3_Na, 253.0602, Δ1.2 ppm).

Acrucipentyn B (**2**): amorphous solids; [α]_D_^20^ +46.2 (*c* 0.09 CH_3_OH); CD (*c* 8.7 × 10^−4^, CH_3_OH), λ_max_ (∆ε) 223 (+0.87) nm, see Supplementary Figure S2; UV (CH_3_OH) *λ*_max_ (log *ε*) 224 (3.93), 200 (3.41) and 194 (3.51) nm, see Supplementary Figure S8; ^1^H and ^13^C NMR data, see Table 1 and Table 2, Supplementary Figures S23–S29; HRESIMS *m*/*z* 229.0634 [M – H]^–^ (calcd. for C_11_H_14_ClO_3_, 229.0637, Δ1.4 ppm), 253.0601 [M + Na]^+^ (calcd. for C_11_H_15_ClO_3_Na, 253.0602, Δ0.5 ppm).

Acrucipentyn C (**3**): amorphous solids; [α]_D_^20^ –127.9 (*c* 0.07 CH_3_OH); CD (*c* 1.2 × 10^−3^, CH_3_OH), λ_max_ (∆ε) 223 (−0.89), 297 (−0.11), 305 (−0.12) nm, see Supplementary Figure S3; UV (CH_3_OH) *λ*_max_ (log *ε*) 270 (3.06), 251 (2.63) and 223 (3.70) nm, see Supplementary Figure S9; ^1^H and ^13^C NMR data, see Table 1 and Table 2, Supplementary Figures S30–S35; HRESIMS *m*/*z* 229.0631 [M – H]^–^ (calcd. for C_11_H_14_ClO_3_, 229.0637, Δ2.5 ppm), 253.0595 [M + Na]^+^ (calcd. for C_11_H_15_ClO_3_Na, 253.0602, Δ2.7 ppm).

Acrucipentyn D (**4**): amorphous solids; [α]_D_^20^ – 94.3 (*c* 0.06 CH_3_OH); CD (*c* 8.8 × 10^−4^, CH_3_OH), λ_max_ (∆ε) 210 (−3.73), 227 (−1.44), 238 (−1.17) nm, see Supplementary Figure S4; UV (CH_3_OH) *λ*_max_ (log *ε*) 259 (3.90), 226 (3.56) and 198 (3.80) nm, see Supplementary Figure S10; ^1^H and ^13^C NMR data, see Table 1 and Table 2, Supplementary Figures S36–S42; HRESIMS *m*/*z* 227.0471 [M – H]^–^ (calcd. for C_11_H_12_ClO_3_, 227.0480, Δ4.0 ppm), 251.0441 [M + Na]^+^ (calcd. for C_11_H_13_ClO_3_Na, 251.0445, Δ1.6 ppm).

Acrucipentyn E (**5**)*:* amorphous solids; [α]_D_^20^ –60.5 (*c* 0.09 CH_3_OH); CD (*c* 8.8 × 10^−4^, CH_3_OH), λ_max_ (∆ε) 199 (−5.11), 226 (−1.19), 237 (−0.83) nm, see Supplementary Figure S5; UV (CH_3_OH) *λ*_max_ (log *ε*) 260 (4.22), 226 (3.77) and 198 (4.10) nm, see Supplementary Figure S11; ^1^H and ^13^C NMR data, see Table 1 and Table 2, Supplementary Figures S43–S49; HRESIMS *m*/*z* 227.0470 [M – H]^–^ (calcd. for C_11_H_12_ClO_3_, 227.0480, Δ4.5 ppm), 251.0441 [M + Na]^+^ (calcd. for C_11_H_13_ClO_3_Na, 251.0445, Δ1.6 ppm).

Acrucipentyn F (**6**): amorphous solids; [α]_D_^20^ +40.3 (*c* 0.11 CH_3_OH); CD (*c* 1.0 × 10^−3^, CH_3_OH), λ_max_ (∆ε) 199 (−4.56), 226 (−0.72), 259 (+1.13) nm, see Supplementary Figure S6; UV (CH_3_OH) *λ*_max_ (log *ε*) 259 (4.05), 226 (3.59) and 201 (3.88) nm, see Supplementary Figure S12; ^1^H and ^13^C NMR data, see Table 1 and Table 2, Supplementary Figures S50–S56; HRESIMS *m*/*z* 191.0704 [M – H]^–^ (calcd. for C_11_H_11_O_3_, 191.0714, Δ5.2 ppm), 215.0677 [M + Na]^+^ (calcd. for C_11_H_12_O_3_Na, 215.0679, Δ0.8 ppm).

### 2.6. Preparation of Acetonides of ***1a*** and ***4a***

To a DMFA solution of **1** (4.0 mg) 2,2-dimethoxypropane (0.5 mL) and catalyst *p*-toluenesulfonic acid (0.8 mg) at room temperature were added and the solution was stirred for 24 h. After the evaporation of the solvent, the product was dissolved in MeOH and purified by reversed-phase HPLC (YMC ODS-A column) eluted with CH_3_CN-H_2_O (50:50) to yield the acetonide product **1a** (1.5 mg). Compound **4** (4.0 mg) was treated similarly and yielded the acetonide product **4a** (1.4 mg).

Acetonide of acrucipentyn A (**1a**): amorphous solids; ^1^H NMR (Acetone-*d*_6_, 500.13 MHz) δ: 5.20 (1H, s, H-4a′), 5.19 (1H, s, H-4b′), 4.37 (1H, t, *J =* 4.3 Hz, H-1), 4.20 (1H, dd, *J =* 8.3; 4.8 Hz, H-2), 4.11 (1H, m, H-4), 4.00 (1H, dd, *J* = 8.3; 2.6 Hz, H-3), 3.50 (1H, dt, *J =* 12.2; 4.4 Hz, H-6), 2.03 (1H, m, H-5b), 1.97 (1H, dt, *J =* 13.7; 4.7 Hz, H-5a), 1.85 (3H, s, H_3_-5′), 1.47 (3H, s, H_3_-3″), 1.34 (3H, s, H_3_-2″); ^13^C NMR (Acetone-*d*_6_, 125.77 MHz) δ: 128.9 (C-3′), 122.0 (C-4′), 110.3 (C-1″), 90.3 (C-1′), 84.1 (C-2′), 80.5 (C-2), 77.4 (C-1), 70.7 (C-4), 67.1 (C-3), 35.0 (C-6), 29.3 (C-3″), 27.2 (C-2″), 26.5 (C-5), 24.6 (C-5′), see Supplementary Figures S57–S58; HRESIMS *m*/*z* 293.0912 [M + Na]^+^ (calcd. for C_14_H_19_ClO_3_Na, 293.0915, Δ0.9 ppm).

Acetonide of acrucipentyn D (**4a**): amorphous solids; ^1^H NMR (Acetone-*d*_6_, 700.13 MHz) δ: 5.33 (1H, t, *J =* 0.9 Hz, H-4a′), 5.31 (1H, t, *J =* 1.7 Hz, H-4b′), 6.11 (1H, d, *J =* 3.0 Hz, H-5), 4.63 (1H, dd, *J =* 5.7; 0.9 Hz, H-1), 4.58 (1H, m, H-4), 4.57 (1H, t, *J* = 5.7 Hz, H-2), 4.38 (1H, dd, *J =* 5.6; 3.5 Hz, H-3), 1.91 (3H, t, *J =* 1.4 Hz, H_3_-5′), 1.37 (3H, s, H_3_-2″/H_3_-3″), 1.33 (3H, s, H_3_-2″/H_3_-3″); ^13^C NMR (Acetone-*d*_6_, 125.77 MHz) δ: 137.7 (C-5), 130.0 (C-6), 129.3 (C-3′), 122.8 (C-4′), 110.6 (C-1″), 91.3 (C-2′), 87.9 (C-1′), 77.1 (C-2), 74.6 (C-1), 66.0 (C-4), 62.8 (C-3), 27.8 (C-2″/C-3″), 26.0 (C-2″/C-3″), 23.4 (C-5′), see Supplementary Figures S63–S64; HRESIMS *m*/*z* 291.0758 [M + Na]^+^ (calcd. for C_14_H_17_ClO_3_Na, 291.0758, Δ0.9 ppm).

### 2.7. Preparation of (S)-MTPA and (R)-MTPA Esters of ***1a***

To a pyridine solution of **1a** (0.7 mg) 4-dimethylaminopyridine (a few crystals) and (*S*)-MTPA-Cl (10 μL) at room temperature were added, and the solution was stirred for 24 h. After the evaporation of the solvent, the residue was purified by RP HPLC on a YMC ODS-AM column eluted with CH_3_CN-H_2_O (70:30) to afford the (*R*)-MTPA ester (**1a-1**). The (*S*)-MTPA ester (**1a-2**) was prepared in a similar manner using (*R*)-MTPACl. ^1^H NMR and COSY data, see Supplementary Figure S59–S62.

(*R*)-MTPA ester of **1a**: ^1^H NMR (Acetone-*d*_6_, 500.13 MHz) δ: 5.56 (1H, brs, H-4), 5.22 (1H, s, H-4a′), 5.20 (1H, s, H-4b′), 4.32 (1H, brs, H-3), 4.30 (1H, brs, H-1), 4.09 (1H, dd, *J* = 8.1; 4.9 Hz, H-2), 2.80 (1H, m, H-6), 2.24 (1H, t, *J =* 13.7 Hz, H-5a), 2.09 (1H, m, H-5b), 1.84 (3H, s, H_3_-5′), 1.50 (3H, s, H_3_-3″), 1.33 (3H, s, H_3_-2″). HRESIMS *m/z* 509.1310 [M + Na]^+^ (calcd for C_24_H_26_ClF_3_O_5_, 509.1313)

(*S*)-MTPA ester of **1a**: ^1^H NMR (Acetone-*d*_6_, 500.13 MHz) δ: 5.65 (1H, brs, H-4), 5.24 (1H, s, H-4a′), 5.21 (1H, s, H-4b′), 4.29 (1H, d, *J =* 7.9 Hz, H-3), 4.44 (1H, t, *J =* 4.1 Hz, H-1), 4.06 (1H, dd, *J* = 8.0; 5.2 Hz, H-2), 3.27 (1H, dt, *J* = 12.8; 4.1 Hz, H-6), 2.33 (1H, t, *J =* 13.4 Hz, H-5a), 2.22 (1H, dt, *J* = 14.6; 4.7 Hz, H-5b), 1.85 (3H, s, H_3_-5′), 1.50 (3H, s, H_3_-3″), 1.34 (3H, s, H_3_-2″). HRESIMS *m/z* 509.1307 [M + Na]^+^ (calcd for C_24_H_26_ClF_3_O_5_, 509.1313).

### 2.8. An Epoxy Ring-Opening Reaction

Next, 2,2-dimethylpropanoyl chloride (0.5 mL) was added to an aqueous solution of **6** (12.0 mg) at room temperature, and the solution was stirred for 12 h. After the evaporation of the solvent, the product was dissolved in MeOH and purified by reversed-phase HPLC (Phenomenex Hydro-RP column) eluted with CH_3_OH-H_2_O (50:50) to yield compound **4** (3.7 mg) and compound **5** (6.4 mg).

### 2.9. Cell Lines and Culture Conditions

The human normal cell lines HEK 293T and MRC-9 cell lines were purchased from ECACC (Salisbury, UK). Human normal cell lines PNT2 and RWPE-1 as well as human cancer cell lines PC-3, DU145, 22Rv1, VCaP and LNCaP, as well as a human normal prostate line were purchased from ATCC (Manassas, VA, USA). Human normal cell line HUVEC (passage 11) was kindly donated by Prof. Sonja Loges (University Medical Center Hamburg-Eppendorf, Hamburg, Germany). The human keratinocytes cell line HaCaT was kindly provided by Prof. N. Fusenig (Cancer Research Centre, Heidelberg, Germany). All the cells had a passage number ≤ 30.

Cells were incubated in humidified 5% CO_2_ at 37 °C. The cells were continuously kept in a culture for 3 months maximum and regularly checked for mycoplasma infection using MycoAlert™ PLUS Mycoplasma Detection Kit (Lonza, Karlsruhe, Germany) and stable phenotype using light microscopy [17].

The following culture media were used: RPMI medium supplemented with Glutamax^TM^-I (Invitrogen, Paisley, UK) with 10% fetal bovine serum (FBS, Invitrogen) and 1% penicillin/streptomycin (Invitrogen) for PC-3, DU145, LNCaP, 22Rv1 and PNT2 cells. DMEM medium supplemented with Glutamax^TM^-I (Invitrogen) containing 10% FBS and 1% penicillin/streptomycin (Invitrogen) for MRC-9, HEK 293 and VCaP cells. Clonetics^®^ EGM^TM^-2 SingleQuots^®^ medium (Lonza, Walkersville, MD, USA) containing 10% FBS for RWPE-1 cells. Clonetics^®^ EGM^TM^-2 SingleQuots^®^ medium (Lonza, Walkersville, MD, USA) containing 10% FBS for HUVEC cells. DMEM medium (BioloT, St. Petersburg, Russia) containing 10% FBS and 1% penicillin/streptomycin (Invitrogen) for HaCaT cells.

### 2.10. MTT Assay

Cytotoxicity of the isolated compounds to mammalian cells was evaluated using an MTT assay as previously reported with minor modification [18]. In brief, 6000 cells/well were seeded in 96-well planes in 100 µL/well and were incubated overnight. Then the media were exchanged with fresh media containing tested compounds in different concentrations. Following 72 h of incubation, 10 µL of MTT solution (5 mg/mL, Sigma-Aldrich, Munich, Germany) was added to each well, the cells were incubated for 2–4 h. Then the media was carefully aspirated and the plates were dried for 2 h. Then 50 µL/well of DMSO was added to each well to dissolve formazan crystals and the absorbance was measured using plate reader according to the manufacture’s protocol. The data were analyzed and the IC_50_s values were calculated using GraphPad Prism software v.9.1.1 (GraphPad Software, San Diego, CA, USA).

### 2.11. Sortase Activity Inhibition Assay

The enzymatic activity of sortase A from *Staphylococcus aureus* was determined using SensoLyte 520 Sortase A Activity Assay Kit * Fluorimetric * (AnaSpec AS-72229, AnaSpec, San Jose, CA, USA) in accordance with the manufacturer’s instructions. Substances **1**–**6** were dissolved in DMSO and diluted with reaction buffer to obtain a final concentration of 0.8% DMSO, which did not affect enzyme activity. DMSO at a concentration of 0.8% was used as a control. PCMB (4-(hydroxymercuri)benzoic acid) was used as sortase A enzyme activity inhibitor. Fluorescence was measured with a plate reader PHERAStar FS (BMG Labtech, Offenburg, Germany) for 60 min, with a time interval of 5 min. The data were processed with MARS Data Analysis v. 3.01R2 (BMG Labtech, Offenburg, Germany). The results were presented as relative fluorescent units (RFUs) and percentage of the control data, calculated using STATISTICA 10.0 software [14].

### 2.12. Antimicrobial Activity

The antibacterial activity of compounds **1**–**6** was evaluated as described previously [19].

The bacterial culture of *Staphylococcus aureus* ATCC 21027 (Collection of Marine Microorganisms PIBOC FEBRAS) was cultured in a Petri dish at 37 °C for 24 h on solid medium Mueller Hinton broth with agar—16.0 g/L.

The assays were performed in 96-well microplates in appropriate Mueller Hinton broth. Each well contained 90 µL of bacterial suspension (10^9^ CFU/mL). Then, 10 µL diluted at concentrations from 1.5 µM to 100.0 µM using two-fold dilution was added to compounds **1**–**6** (DMSO concentration < 1%). Culture plates were incubated overnight at 37 °C, and the OD_620_ was measured using a Multiskan Spectrum spectrophotometer (Thermo Labsystems Inc., Beverly, MA, USA). Gentamicin was used as a positive control in concentration 1 mg/mL; 1% DMSO solution in PBS as a negative.

### 2.13. Biofilm Formation

The inhibition of the reducing biofilm formation and growth was assessed using the crystal violet biofilm assay as described [20]. Mueller Hinton broth was inoculated with 10^9^ CFU/mL of *S. aureus* overnight cultures. A total of 90 µL of this cell suspension was then dispensed into 96-well microtiter plates containing 10 µL of different concentrations of compounds **1**–**6**. After 24 h growth at 37 °C the plates were washed with PBS to remove unbound cells. Next, the wells were stained with 0.1% crystal violet solution for 10 min at 37 °C. At the completion of the incubation, plates were washed 3 times with PBS and dried. Then, the crystal violet dye from the biofilm was solubilized with 100 µL of ethanol. A total of 100 µL of this solution was then moved to a new microtiter plate for absorbance measurement at λ = 570 nm. The results were reported as percent inhibition normalized to the wild-type control.

### 2.14. Co-Cultivation of HaCaT Cells with S. aureus

Co-cultivation of HaCaT cells with *S. aureus* was carried out as described [21]. HaCaT cells at a concentration of 1.5×10^4^ cells per well were seeded in 96-well plates for 24 h. Then, culture medium in each well was changed with *S. aureus* suspension (10^2^ CFU/mL) in full DMEM medium. Fresh DMEM medium without *S. aureus* suspension was added in other wells as needed. Compounds **1**–**6** at a concentration of 10 μM were added in wells after 1 h. HaCaT cells and *S. aureus* were cultured at 37 °C in a humidified atmosphere with 5% (*v*/*v*) CO_2_ for 48 h.

After incubation, the plate was centrifuged at 250× *g* for 10 min and 50 µL of supernatant from each well was transferred into the corresponding wells of an optically clear 96-well plate. An equal volume of the reaction mixture (50 µL) from LDH Cytotoxicity Assay Kit (Abcam, Cambridge, UK) was added to each well and incubated for up to 30 min at room temperature. The absorbance of all samples was measured at λ = 450 nm using a Multiskan FC microplate photometer (Thermo Scientific, Waltham, MA, USA) and expressed in optical units (o.u.).

### 2.15. Statistical Analysis

All the experiments were performed in biological triplicates. Statistical analyses were performed using GraphPad Prism v.9.1.1 (GraphPad Software, San Diego, CA, USA) or STATISTICA 10.0 software. The data are reported as mean ± SD (standard deviation). For the analysis of statistical differences between the control and drug-exposed group, a one-way ANOVA test followed by Dunnett’s post-hoc test was used. Asterisk (*) indicates statistically significant difference between the treated group and control group if *p* < 0.05.

## 3. Results and Discussion

### 3.1. Isolated Compounds from Asteromyces cruciatus

**Figure 1:**
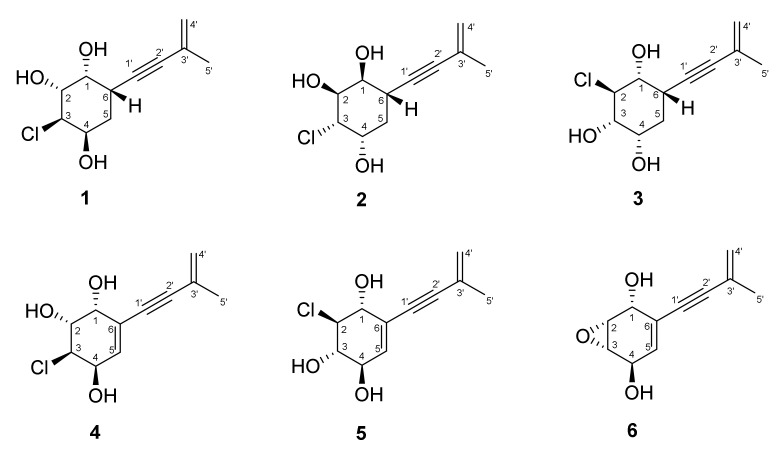
Chemical structures of **1**–**6**.

The fungus *Asteromyces cruciatus* was cultivated on a solid rice medium for 21 days. The ethyl acetate extract of the mycelium was fractionated on silica gel, followed by C_18_-SiO_2_-column chromatography, and reversed-phase HPLC to produce compounds **1**–**6** (Figure 1).

### 3.2. Structural Characterization of New Compounds

**Figure 2:**
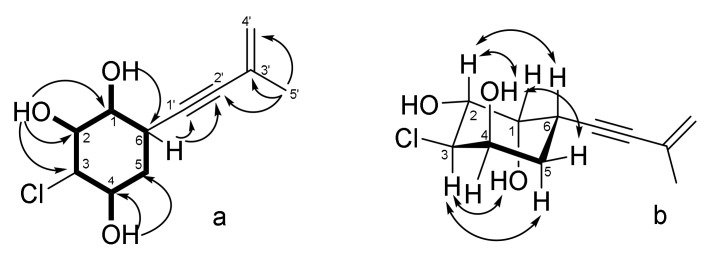
Key ^1^H–^1^H COSY, ^1^H–^13^C HMBC (**a**) and ROESY (**b**) correlations of **1**.

The molecular formula of **1** was determined as C_11_H_15_ClO_3_, based on the analysis of HRESIMS (*m/z* 229.0625 [M – H]^–^ calcd for C_11_H_14_ClO_3_, 229.0637), showing the characteristic isotope pattern with one chlorine atom, and confirmed by NMR data. A close inspection of the ^1^H and ^13^C NMR data (Table 1 and Table 2; Figures S13–S19) of **1** by DEPT and HSQC revealed the presence of one methyl (δ_H_ 1.80, δ_C_ 23.5), two methylenes (δ_H_ 1.65, 1.92, δ_C_ 33.1; δ_H_ 5.14, 5.18, δ_C_ 120.7), and five methines (δ_H_ 2.96, δ_C_ 28.5; δ_H_ 4.00, δ_C_ 65.7; δ_H_ 3.92, δ_C_ 68.2; δ_H_ 3.57, δ_C_ 70.2 and δ_H_ 3.81, δ_C_ 71.9), including three oxygen-bearing methines, one *sp^2^* quaternary carbon and one triple bond (δ_C_ 81.7 and 91.1). The ^1^H–^1^H COSY correlations of H-1(OH)/H-2(OH)/H-3/H-4(OH)/H_2_-5/H-6/H-1 together with the ^1^H-^13^C HMBC correlations (Figure 2) OH-1 (δ_H_ 4.89)/C-6 (δ_C_ 28.5); OH-2 (δ_H_ 4.92)/C-1 (δ_C_ 71.9), C-2 (δ_C_ 70.2), C-3 (δ_C_ 65.7) and OH-4 (δ_H_ 5.06)/C-4 (δ_C_ 68.2), C-5 (δ_C_ 33.1) indicated the presence of a penta-substituted cyclohexane ring and the location of the hydroxy groups at C-1, C-2 and C-4 in **1**. These data, as well as the chemical shifts of CH-3 (δ_H_ 4.00, δ_C_ 65.7), indicated the location of the chlorine atom at C-3.

The HMBC correlations from H-4′a (δ_H_ 5.14) to C-2′ (δ_C_ 81.7) and C-3′ (δ_C_ 126.8), from H_3_-5′ (δ_H_ 1.80) to C-2′, C-3′ and C-4′ (δ_C_ 120.7) and cross ^1^H–^1^H COSY correlations between H_2_-4′ and H_3_-5′ revealed the presence of a 3-methyl-3-buten-1-ynyl side chain in **1**. The correlations H-6 (δ_H_ 3.19)/C-1′ (δ_C_ 87.5) and C-2′ (δ_C_ 84.1) observed in the HMBC spectrum, recorded in CDCl_3_ (Figures S20–S22), established the position of the side chain at C-6.

**Figure 3:**
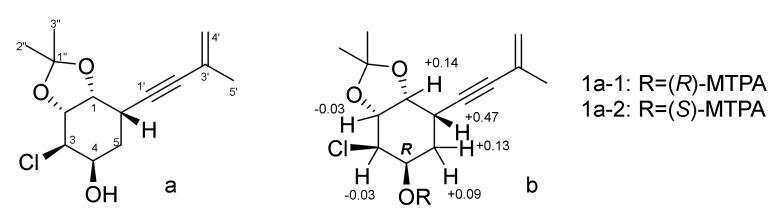
Chemical structure of **1a** (**a**) and ∆δ(δ_S_-δ_R_) values (in ppm) for MTPA esters of **1a** (**b**).

The relative configurations of **1** were assigned based on ROESY correlations (Figure 2) H-6 (δ_H_ 2.96)/H-2 (δ_H_ 3.57); OH-4 (δ_H_ 5.06)/H-5β (δ_H_ 1.65) and H-2; H-3 (δ_H_ 4.00)/H-2β (δ_H_ 1.92), OH-1 (δ_H_ 4.89) and ^1^H-^1^H coupling constants (Table 1). For further investigation, we analyzed the stereoconfigurations of diol at C-1 and C-2 and for protection of these groups before MTPA-esters obtaining the acetonide derivative (**1a**) of compound **1** (Figure 3) was prepared. The small coupling constant (*J*_1,2_ = 4.3 Hz) and dissimilar magnetic environment of acetonide methyls (Δ = 0.13 ppm) (Figure S57) indicate an *erythro* configuration of the diol group at C-1 and C-2 [22]. The absolute configuration of **1** was established by the modified Mosher’s method [23]. Esterification of **1a** with (*S*)- and (*R*)-MTPA chloride occurred at the C-4 hydroxy group to yield the (*R*)-and (*S*) MTPA esters **1a-1** and **1a-2**, respectively. The observed chemical shift differences Δδ(δ*_S_*-δ*_R_*) (Figure 3) indicated the 4*R* configuration and, therefore, the absolute configurations of **1** were established as 1*R*,2*R*,3*R*,4*R*,6*S*. Compound **1** was named acrucipentyn A.

The HRESIMS of **2** and **3** showed the peaks of [M–H]^–^ at *m/z* 229.0634 and *m/z* 229.0631, respectively. These data, coupled with ^13^C NMR spectral data (DEPT), established the molecular formulas of **2** and **3** as C_11_H_15_ClO_3_ for both. A close inspection of the ^1^H and ^13^C NMR data (Table 1 and Table 2 and Figures S23–S35) of **2** and **3** by DEPT and HSQC revealed the presence of a penta-substituted cyclohexane ring with three hydroxy groups and a 3-methyl-3-buten-1-ynyl side chain.

The main ^1^H–^1^H COSY and HMBC correlations (Figures S26 and S28) indicated that compound **2** has the same planar structure as **1**. The relative configuration of **2** was assigned based on ^1^H-^1^H vicinal coupling constants (Table 1) and ROESY (Figure S29) correlations H-6 (δ_H_ 2.87)/H-4 (δ_H_ 4.22) and H-5β (δ_H_ 1.91)/H-1 (δ_H_ 3.92). Due to the small amount of compound **2,** the absolute configuration establishing by Mosher’s method was impossible. Compound **2** was named acrucipentyn B.

The ^1^H–^1^H COSY correlations of H-1(OH)/H-2/H-3(OH)/H-4(OH)/H_2_-5/H-6 together with the ^1^H-^13^C HMBC correlations (Figure S34) OH-1 (δ_H_ 4.38)/C-1 (δ_C_ 74.2), C-2 (δ_C_ 68.9) and C-6 (δ_C_ 35.0); OH-3 (δ_H_ 4.50)/C-2, C-3 (δ_C_ 79.5) and C-4 (δ_C_ 71.1), and OH-4 (δ_H_ 4.04)/C-3, C-4 and C-5 (δ_C_ 34.2) indicated the location of the hydroxy groups at C-1, C-3, C-4 and a chlorine atom at C-2 in a penta-substituted cyclohexane ring of **3**. The structure of the 3-methyl-3-buten-1-ynyl side chain and its position at C-6 in **3** were determined by HMBC correlations (Figure S34), as for acrucipentyn A (**1**).

The relative configurations of the chiral centers in **3** were determined based on ^1^H-^1^H coupling constants (Table 1). Using the Mosher’s method to determine absolute configurations of compound **3** was unsuccessful, due to lability in this compound. Compound **3** was named acrucipentyn C.

The HRESIMS of **4** showed the peak of [M – H]^–^ at *m/z* 227.0471. These data, coupled with ^13^C NMR spectral data (DEPT), established the molecular formula of **4** as C_11_H_13_ClO_3_. The ^1^H and ^13^C NMR (Table 1 and Table 2 and Figures S36–S42), DEPT and HSQC spectra showed the presence of three hydroxy protons (δ_H_ 5.25, 5.17, 5.04), one methyl group (δ_H_ 1.86, δ_C_ 23.0), one olefinic methylene (δ_H_ 5.32, 5.27, δ_C_ 122.2) and five methines (δ_H_ 4.25, δ_C_ 63.3; δ_H_ 4.37, δ_C_ 65.1; δ_H_ 4.06, δ_C_ 67.9; δ_H_ 3.87, δ_C_ 68.6 and δ_H_ 5.92, δ_C_ 136.0), including three oxygen-bearing methines and one olefinic methine, two *sp^2^* quaternary carbons and one triple bond (δ_C_ 88.2 and 89.9).

**Figure 4:**
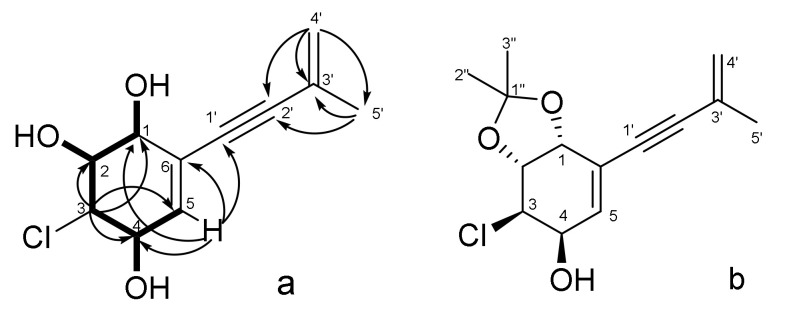
Key ^1^H–^1^H COSY, ^1^H–^13^C HMBC correlations of **4** (**a**) and chemical structure of acetonide derivatives **4a** (**b**).

The HMBC correlations (Figure 4) from H-3 (δ_H_ 4.25) to C-1 (δ_C_ 67.9), C-2 (δ_C_ 68.6), C-4 (δ_C_ 65.1) and C-5 (δ_C_ 136.0); from H-5 (δ_H_ 5.92) to C-1, C-4, C-6 (δ_C_ 123.5) and C-1′ (δ_C_ 88.2), and from OH-4 (δ_H_ 5.25) to C-4, C-5, together with ^1^H–^1^H COSY correlations of H-1(OH)/H-2(OH)/H-3/H-4(OH)/H-5, indicated the presence of a penta-substituted cyclohexene ring with ∆^5,6^ double bond, the location of the hydroxy groups at C-1, C-2, C-4 and a chlorine atom at C-3 in **4**. The structure of the 3-methyl-3-buten-1-ynyl side chain and its position at C-6 in **4** were determined by HMBC correlations (Figure S41), as for acrucipentyn A (**1**).

The relative configuration of **4** was assigned based on ^1^H-^1^H coupling constants (Table 1) and ROESY correlation (Figure S42) H-2 (δ_H_ 3.87)/OH-4 (δ_H_ 5.25). The acetonide derivative (**4a**) of compound **4** (Figure 4) was prepared for further investigation of the stereochemistry at the diol position. The small coupling constant (*J*_1,2_ = 5.7 Hz) and dissimilar magnetic environment of acetonide methyls (Δ = 0.01 ppm) (Figure S63) indicate an *erythro* configuration of the diol group at C-1 and C-2 [21]. Esterification of **4a** with (*S*)- and (*R*)-MTPA-Cl led to destruction of the compound. Etherification of compound **4** with (*S*)- and (*R*)-MTPA chloride resulted in the formation of esters at three hydroxyl groups, which made it impossible to establish the absolute configuration using the modified Mosher’s method. Compound **4** was named acrucipentyn D.

The HRESIMS of **5** showed the peak of [M – H]^–^ at *m/z* 227.0468. These data, coupled with ^13^C NMR spectral data (DEPT), suggested the molecular formula of **5** as C_11_H_13_ClO_3_. The ^1^H and ^13^C NMR data (Table 1 and Table 2 and Figures S43–S49) of **5** revealed the presence of a penta-substituted cyclohexene ring with three hydroxy groups and a 3-methyl-3-buten-1-ynyl side chain, the same as in **4**. The location of the hydroxy groups at C-1, C-3, C-4, a chlorine atom at C-2 and a 3-methyl-3-buten-1-ynyl side chain at C-6 in **5** were determined by ^1^H–^1^H COSY and HMBC correlations (Figures S46 and S48), as for acrucipentyn C (**3**).

The relative configuration of **5** was assigned based on ROESY correlations (Figure S49) H-2 (δ_H_ 3.69)/H-4 (δ_H_ 3.97), OH-1 (δ_H_ 5.73), OH-3 (δ_H_ 5.48); H-3 (δ_H_ 3.31)/H-1 (δ_H_ 4.05), OH-4 (δ_H_ 5.31) and ^1^H-^1^H coupling constants (Table 1). The attempts to obtain an acetonide of compound **5** were unsuccessful. Etherification of compound **5** with (*S*)- and (*R*)-MTPA-Cl resulted in the formation of esters at three hydroxyl groups, which made it impossible to establish the absolute configuration by the modified Mosher’s method. Compound **5** was named acrucipentyn E.

The molecular formula of compound **6** was determined as C_11_H_12_O_3_, based on the analysis of HRESIMS (*m/z* 191.0705 [M – H]^−^, calcd for C_11_H_11_O_3_ 191.0714) and NMR data (Table 1 and Table 2; Figures S50–S56). The ^1^H and ^13^C NMR data for this compound were similar to those obtained for (+)-asperpentyn [24,25], with the exception of the CH-2 and CH-3 proton and carbon signals. These data, as well as the biogenetic relationship of compound **6** with acrucipentyns D (**4**) and E (**5**), led us to suggest a configuration of asymmetric centers for it, different from the known asperpentyns.

**Figure 5:**
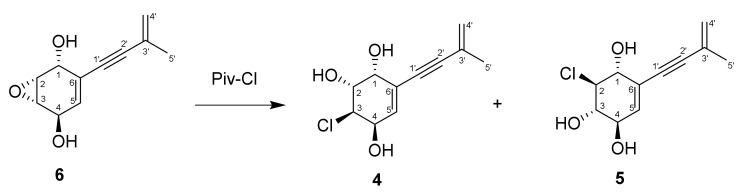
Scheme of an epoxy ring reaction in compound **6**.

The ROESY spectrum data and ^1^H-^1^H coupling constants were useless to establish the relative stereochemistry of **6** unambiguously. Therefore, an epoxy ring-opening reaction was carried out in **6**. The reaction of compound **6** with 2,2-dimethylpropanoyl chloride in an aqueous medium (Figure 5) yielded two products, the spectra of which (^1^H, ^13^C NMR, HRESIMS and CD) were identical to acrucipentyns D (**4**) and E (**5**). The presence of only two reaction products confirmed the S_N2_ mechanism of the epoxy ring opening, which corresponded to the literature data [26]. The orientation of the hydroxyl groups in the reaction products corresponds with the configuration of the epoxy ring in the initial compound. These data made it possible to establish the relative configuration of **6**. Compound **6** was named acrucipentyn F.

It should be noted that acrucipentyn F is a stereoisomer of well-known fungal metabolites (−)-asperpentyn [27,28] and (+)-asperpentyn [24]. To the best of our knowledge, acrucipentyns are the first chlorine-containing asperpentyn-like compounds. However, it should be noted that several other related groups of 3-methylbutenynyl cyclohexanols, e.g., truncateols from the marine-derived fungi *Truncatella angustata* [29,30] and oxirapentyns from the marine-derived fungi *Beauveria felina* KMM 4639 [31], also have chloro-containing members.

### 3.3. Biological Activity

We evaluated the safety and toxicity of compounds **1**–**6** in various human cells. As such, we examined cytotoxicity in ten different human cell lines, including human prostate cells PNT2 and RWPE-1, human embryonic kidney cells HEK 293T, human fibroblast cells MRC-9 and human umbilical vascular endothelial cell line HUVEC, human keratinocytes HaCaT, as well as human prostate cancer cells PC-3, DU145, 22Rv1, VCaP and LNCaP using MTT assay. Indeed, none of the investigated compounds exhibited any significant cytotoxicity at concentrations up to 100 µM, following 72 h of treatment (IC_50_ > 100 µM, Figure S65). Additionally, no morphological changes of the cells exposed to the isolated compounds (100 µM for 72 h) could be detected (Figure S66). Therefore, the isolated acrucipentyns A–F were assumed to be nontoxic to mammalian (human) cells.

**Figure 6:**
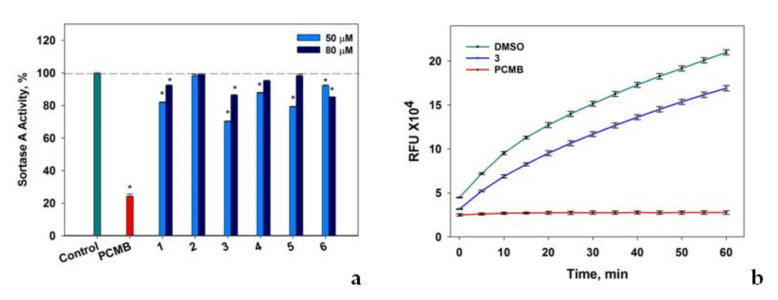
The effect of compounds **1**–**6** on sortase A enzymatic activity. (**a**) The effect of compounds **1**–**6** on sortase A enzymatic activity measured after 10 min of incubation with the substrate. (**b**) The time-dependent effect of compound **3** (50 μM) on sortase A enzymatic activity. Data presented as relative fluorescent units (RFU). DMSO (0.8%) did not show any inhibition activity in comparison with sortase A assay buffer and was used as a control. The sortase inhibitor—4-(hydroxymercuri)benzoic acid (PCMB) in DMSO 0.8% was used as a positive control. All experiments were performed in three independent replicates and the data presented as a mean ± standard error mean (SEM). * indicates the significant differences with *p* ≤ 0.05.

The inhibitory effect of compounds **1**–**6** on sortase A enzyme from *Staphylococcus aureus* activity was investigated to detect their antibacterial potential (Figure 6).

Compounds **1**, **3**, and **5,** at a concentration of 50 μM, significantly decreased sortase A activity by 18%, 30%, and 21%, respectively (Figure 6a). Compounds **4** and **6** showed less significant effects on sortase A enzymatic activity and compound **2** was inactive in this test. It was observed that an increase in concentrations of **1**–**6** up to 80 μM resulted in some decrease in their sortase A inhibitory activity, which was sometimes detected [32]. The inhibitory effect of compound **3** on sortase A activity was detected throughout the entire period of data acquisition (Figure 6b) and the effects of other studied compounds were similar.

**Figure 7:**
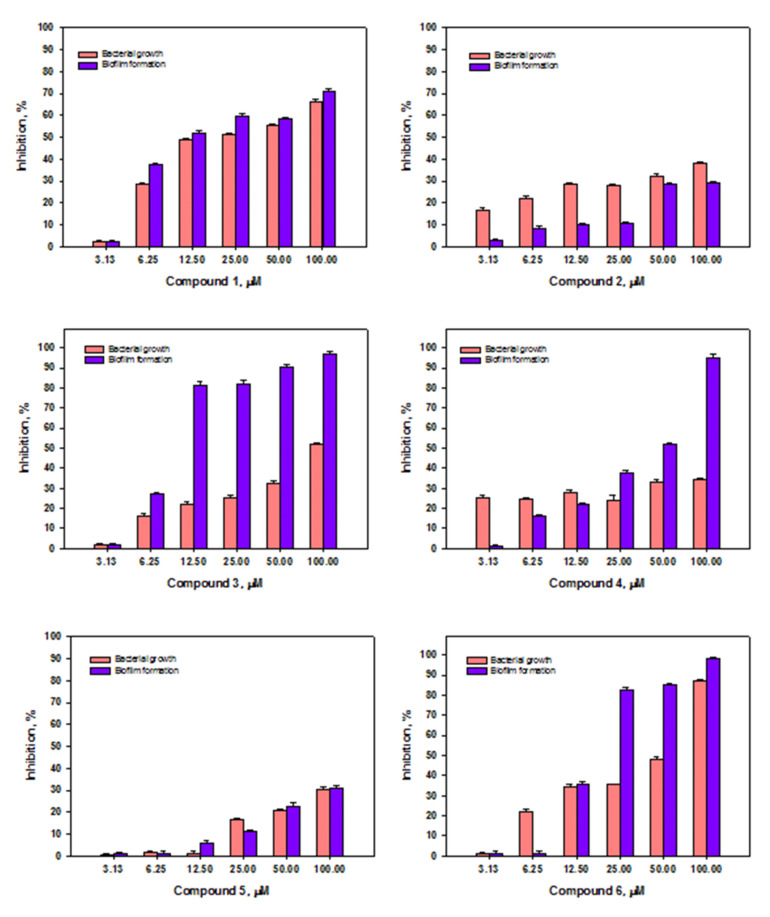
Effect of acrucipentyns A–F (**1**–**6**) on growth and biofilm formation of *Staphylococcus aureus*. All experiments were performed in three independent replicates and data were presented as a mean ± SEM.

To detect the antibacterial activity of isolated acrucipentyns A–E (**1**–**6**), their inhibitory effects on bacterial growth and biofilm formation of *Staphylococcus aureus* were investigated (Figure 7).

Compounds **1** and **6** have shown the most pronounced antimicrobial effects against Gram-positive bacterium *S. aureus*. Compound **6** almost completely inhibited the growth of *S. aureus* at a concentration of 100 μM; a two-fold decrease in the concentration of the substance also halved the antimicrobial activity. Compound **1** at a concentration of 100 μM reduced the bacterial growth by 60%. A decrease in concentration to 12.5 μM reduced antimicrobial activity up to 50%. Compound **3** at 100 μM inhibited *S. aureus* growth by 50%. A decrease in the concentration of compound **3** led to a two-fold decrease in activity. The antimicrobial effects of **2**, **4,** and **5**, even at the highest used concentration of 100 μM, do not exceed 50% inhibition of bacterial growth.

When studying the effect of compounds **1**–**6** on the ability to inhibit the biofilm formation by Gram-positive bacteria *S. aureus*, it was noted that compounds **3**, **4**, and **6** have the most pronounced inhibitory activity at a concentration of 100 μM, in the case of which the formation of biofilms is almost absent. A high level of inhibition of biofilm formation in the case of substances **3** and **6** is kept up to concentrations of 12.5 and 25 μM, respectively. When substance **4** is diluted twice, its effect on biofilm formation is halved. Compound **1** inhibits the biofilm formation at concentrations of 12.5–100 μM by 50–70%, respectively. Compounds **2** and **5** at a concentration of 100 µM inhibited biofilm formation by 30%.

Sortase A is an essential component of *S. aureus* virulence because it is responsible for the covalent anchoring of many virulent factors of Gram-positive bacteria onto the cell wall (Appendix A) and, as a result, sortase A plays a key role in the pathogenic processes of *S. aureus* infection [33]. The decrease in sortase activity leads to the abolition of bacterial adhesion to mammalian cells and, thus, is one of the mechanisms preventing the formation of biofilms, which are the predominant form of bacterial existence [34].

The inhibitory effect of investigated compounds **1**, **3**, **4**, and **6** on the biofilm formation correlated with an ability to affect the activity of sortase A. Compound **2**, which did not show a significant effect on biofilm formation, also did not have any effect on the sortase A activity. Opposite to this, compound **5**, in the same experiments, had a significant effect on the activity of sortase A, but had a weaker inhibitory effect on the biofilm formation, in comparison with compounds **1**, **3**, **4**, and **6**. Thus, substances **1**–**6** can be assumed as anti-Staphylococcal agents.

**Figure 8:**
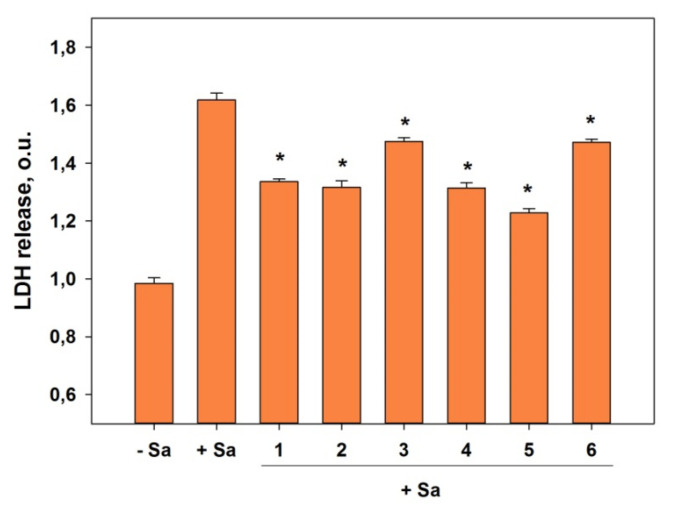
Effect of acrucipentyns A–F (**1**–**6**) on LDH release from human keratinocytes HaCaT co-cultivated with *Staphylococcus aureus* (Sa) for 48 h. All compounds were tested at a concentration of 10 µM. All experiments were performed in triplicates and data are presented as a mean ± SEM. The difference between control (without Sa) and HaCaT/Sa co-cultivation was statistically significant with *p* < 0.05 (one-way ANOVA test). Asterisk (*) indicates significant differences (*p* < 0.05) between HaCaT/Sa without compounds and HaCaT/Sa with compounds variants.

To further confirm their antibacterial properties in a model of infectious damage to human cells, we investigated their effects on lactate dehydrogenase (LDH) release from human keratinocytes HaCaT, co-cultivated with *S. aureus* (Figure 8).

In normal conditions, LDH weakly releases from cells to culture media. *S. aureus* caused a significant increase in the LDH release from keratinocytes during co-cultivation. The addition of compounds **1**–**6** at a concentration of 10 μM reduced the LDH release by 30–50%. The greatest effect was registered for compounds **1**, **2**, **4,** and **5**.

Interestingly, compounds **2** and **4**, which showed significant activity in co-cultivation HaCaT cells with *S. aureus*, did not show high antibacterial and anti-biofilm-forming activity, in contrast to substances **1** and **5**. We assume that the cytoprotective effect of substances **1**–**6** in the in vitro infectious skin lesions could be due not only to their anti-*S. aureus* effects, but also to their anti-inflammatory and other cytoprotective effects. Recently, we observed similar dual effects during an investigation on flavuside B, an inhibitor of sortase A enzymatic activity derived from fungi. Flavuside B was able to inhibit *S. aureus* growth and biofilm formation, as well as protect HaCaT keratinocytes against *S. aureus* infection in a co-culture model via an anti-inflammatory pathway [14].

Our work is the very first detailed investigation of anti-Staphylococcal activity of isoprenylated cyclohexanols. To the best of our knowledge, earlier, only oxirapentyns A and D were found as antimicrobial agents among related compounds [35]. Thus, this group of secondary metabolites of marine fungi is interesting for future study, including their structure–activity relationships.

## 4. Conclusions

Six new isoprenylated cyclohexanols acrucipentyns A–F (**1**–**6**) were isolated from the alga-derived fungus *Asteromyces cruciatus* KMM 4696. The absolute configuration of acrucipentyn A was assigned by the modified Mosher’s method and ROESY data. Acrucipentyns A–E are the very first members of chlorine-contained monocyclic cyclohexanols containing a 3-methylbutenynyl unit. The compounds have shown inhibitory activity against sortase A from *Staphylococcus aureus,* as well as inhibition of *S. aureus* growth and biofilm formation, while no cytotoxicty to mammalian cells was observed. Moreover, acrucipentyns A–F (**1**–**6**) protected human keratinocytes HaCaT from *S. aureus* toxicity in skin infection in an in vitro model. Thus, the isolated compounds hold a good potential as antimicrobal agents and should be further investigated.

## Figures and Tables

**Table 1:** ^1^H NMR data (*δ* in ppm, *J* in Hz) for compounds (**1**–**6**).

Position	1 ^a^	2 ^a^	3 ^b^	4 ^c^	5 ^c^	6 ^d^
1	3.81, brs	3.70, m	3.68, dt (10.4, 5.4)	4.06, brt (4.2)	4.05, t (8.2)	4.50, brs
2	3.57, ddd (10.2, 6.8, 2.6)	3.83, brs	3.91, t (9.9)	3.87, ddd (8.5, 5.2, 4.0)	3.69, dd (11.2, 8.6)	3.60, t (3.3)
3	4.00, dd (10.3, 2.3)	4.18, t (4.1)	3.32, td (9.8, 4.6)	4.25, dd (8.6, 3.9)	3.31, ddd (11.3, 7.7, 6.2)	3.50, m
4	3.92, brs	4.03, dq (12.0, 3.9)	3.81, m	4.37, brq (4.4)	3.97, m	4.53, d (4.8)
5	α: 1.92, td (12.9, 2.1) β: 1.65, dt (13.0, 3.2)	1.74, dt (12.4, 3.9) 1.65, q (12.1)	1.63, ddd (13.3, 11.8, 4.1) 2.01, ddd (13.3, 4.5, 3.2)	5.92, d (4.2)	5.82, brt (2.0)	6.04, dt (5.0, 2.0)
6	2.96, dt (12.6, 2.5)	2.73, td (12.0, 3.9)	3.21, q (4.0)			
4′	a: 5.14, s b: 5.18, s	a: 5.16, s b: 5.19, s	5.21, m	a: 5.27, s b: 5.32, s	a: 5.27, s b: 5.31, s	a: 5.30, s b: 5.36, s
5′	1.80, s	1.81, s	1.86, t (1.2)	1.86, s	1.86, s	1.92, s
1-OH	4.89, d (4.3)	4.77, d (6.7)	4.38, d (5.6)	5.04, d (6.7)	5.73, d (7.9)	
2-OH	4.92, d (7.0)	5.40, brs		5.17, d (5.5)		
3-OH			4.50, d (4.4)		5.48, d (6.0)	
4-OH	5.06, d (4.2)	4.90, d (4.9)	4.04, d (3.4)	5.25, brd (5.8)	5.31, d (5.4)	

^a^ Chemical shifts were measured at 700.13 MHz in DMSO-d_6_. ^b^ Chemical shifts were measured at 700.13 MHz in acetone-d_6_. ^c^ Chemical shifts were measured at 500.13 MHz in DMSO-d_6_. ^d^ Chemical shifts were measured at 500.13 MHz in CDCl_3_.

**Table 2:** ^13^C NMR data (*δ* in ppm) for compounds **1**–**6**.

Position	1 ^a^	2 ^a^	3 ^b^	4 ^c^	5 ^c^	6 ^d^
1	71.9, CH	68.7, CH	74.2, CH	67.9, CH	72.9, CH	65.2, CH
2	70.2, CH	72.5, CH	68.9, CH	68.6, CH	68.3, CH	53.5, CH
3	65.7, CH	66.1, CH	79.5, CH	63.3, CH	74.8, CH	55.3, CH
4	68.2, CH	64.0, CH	71.1, CH	65.1, CH	71.2, CH	63.0, CH
5	33.1, CH_2_	33.4, CH_2_	34.2, CH_2_	136.0, CH	137.2, CH	131.4, CH
6	28.5, CH	30.3, CH	35.0, CH	123.5, C	123.8, C	123.5, C
1′	91.1, C	91.7, C	88.4, C	88.2, C	86.8, C	85.1, C
2′	81.7, C	81.7, C	86.3, C	89.9, C	90.7, C	93.5, C
3′	126.8, C	126.7, C	128.6, C	126.2, C	126.2, C	126.2, C
4′	120.7, CH_2_	120.7, CH_2_	121.4, CH_2_	122.2, CH_2_	122.2, CH_2_	123.5, CH_2_
5′	23.5, CH_3_	23.5, CH_3_	23.8, CH_3_	23.0, CH_3_	23.0, CH_3_	23.2, CH_3_

^a^ Chemical shifts were measured at 176.04 MHz in DMSO-d_6_. ^b^ Chemical shifts were measured at 75.47 MHz in acetone-d_6_.^c^ Chemical shifts were measured at 125.77 MHz in DMSO-d_6_. ^d^ Chemical shifts were measured at 125.77 MHz in CDCl_3_.

**Figure A1:**
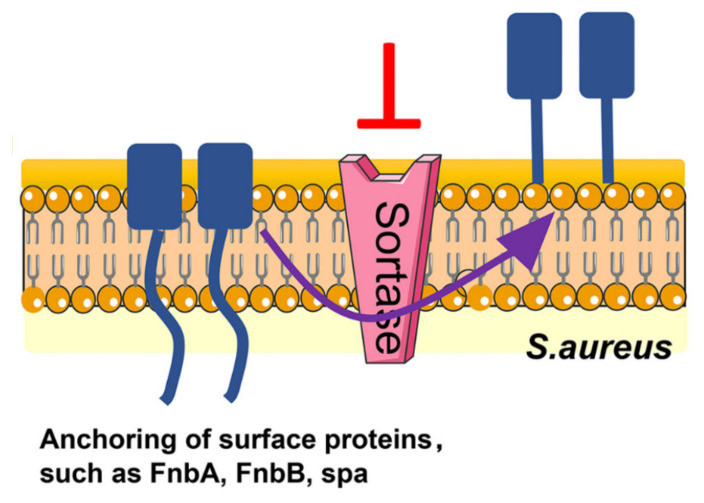
Representation of sortase A role [36].

## Data Availability

Data are contained within the article or Supplementary Material.

## Supplementary Materials

The following are available online at https://www.mdpi.com/article/10.3390/jof8050454/s1, Figures S1–S12: CD and UV spectra of compound **1**–**6**, Figures S13–S22, S23–S28, S29–S35, S36–S43, S44–S49, S50–S56: NMR spectra of compounds **1**–**6**, Figures S57–S58, S59–S60, S61–S62, S63–S64: NMR spectra of compounds **1a**, **1a-1**, **1a-2**, **4a**, Figure S65: Viability of the various cells exposed to the tested compounds, Figure S66: Microphotographs of human cells exposed to the tested compounds.
