# Supplementary material for: New Antibacterial Chloro-Containing Polyketides from the Alga-Derived Fungus Asteromyces cruciatus KMM 4696
Source: J Fungi (Basel). 2022 Apr 27;8(5):454. doi: 10.3390/jof8050454 (PMC9147975; doi:10.3390/jof8050454)
Supplement: Supplementary file 1 [file jof-08-00454-s001.zip › jof-1617096-supplementary.pdf]

SUPPLEMENTARY MATERIAL

New Antibacterial Chloro-Containing Polyketides from the Alga-Derived Fungus *Asteromyces cruciatus* KMM 4696

Olesya I. Zhuravleva[1,2,*], Galina K. Oleinikova[1], Alexandr S. Antonov[1], Natalia N. Kirichuk[1], Dmitry N. Pelageev[1], Anton B. Rasin[1], Alexander S. Menshov[1], Roman S. Popov[1], Natalya Yu. Kim[1], Ekaterina A. Chingizova[1], Artur R. Chingizov[1], Olga O. Volchkova[2], Gunhild von Amsberg[3,4], Sergey A. Dyshlovoy[2,3,4], Ekaterina A. Yurchenko[1] , Irina V. Guzhova[5] and Anton N. Yurchenko[1]

[1] G.B. Elyakov Pacific Institute of Bioorganic Chemistry, Far Eastern Branch of the Russian Academy of Sciences, Prospect 100-letiya Vladivostoka, 159, Vladivostok 690022, Russia; antonov_as@piboc.dvo.ru(A.S.A.); oleingk@mail.ru (O.G.K.); sheflera@bk.ru (N.N.K.) menshov90@piboc.dvo.ru (A.S.M.); pelageev@mail.ru (D.N.P.); abrus__54@mail.ru (A.B.R.); popov_rs@piboc.dvo.ru (R.S.P.); kim_ny@piboc.dvo.ru (N.Y.K.); martyyas@mail.ru (E.A.C.); chingizov_ar@piboc.dvo.ru (A.R.C.); eyurch@piboc.dvo.ru (E.A.Y.); yurchenkoan@piboc.dvo.ru (A.N.Y.)

[2] Institute of High Technologies and Advanced Materials, Far Eastern Federal University, 10 Ajax Bay, Russky Island, Vladivostok 690922, Russia; s.dyshlovoy@uke.de (S.A.D.); olavol1309@gmail.com (O.O.V.)

[3] Laboratory of Experimental Oncology, Department of Oncology, Hematology and Bone Marrow Transplantation with Section Pneumology, Hubertus Wald-Tumorzentrum, University Medical Center Hamburg-Eppendorf, 20246 Hamburg, Germany; g.von-amsberg@uke.de

[4] Martini-Klinik Prostate Cancer Center, University Hospital Hamburg-Eppendorf, 20246 Hamburg, Germany

[5] Institute of Cytology Russian Academy of Sciences, Tikhoretskiy Ave. 4, St. Petersburg 194064, Russia; irina.guzhova@incras.ru (I.V.G.)

* Correspondence: zhuravleva.oi@dvfu.ru; Tel.: +7-423-231-1168

New Antibacterial Chloro-Containing Polyketides from the Alga-Derived Fungus *Asteromyces cruciatus* KMM 4696

Abstract: Six new polyketides acrucipentyns A–F (**1**–**6**) were isolated from the alga-derived fungus *Asteromyces cruciatus* KMM 4696. Their structures were established based on spectroscopic methods. The absolute configurations of some compounds were assigned by the modified Mosher's method, ROESY data analysis as well as biogenetic considerations. Acrucipentyns A–E were identified to be the very first examples of chlorine-containing asperpentyn-like compounds. The cytotoxic and antimicrobial activities of the isolated compounds were examined. Acrucipentyns A–F were found as antimicrobial agents which inhibited sortase A enzyme activity, bacterial growth and biofilm formation of *Staphylococcus aureus* and decreased LDH release from human keratinocytes HaCaT in *S. aureus* skin infection *in vitro* model.

Keywords: *Asteromyces cruciatus*; marine fungi; secondary metabolites; polyketides; sortase A; chlore-containing metabolites; *Staphylococcus aureus*; antibacterial activity; biofilm formation.

Content

Experimental Section 5
Figure S1. CD spectrum of **1** 5
Figure S2. CD spectrum of **2** 5
Figure S3. CD spectrum of **3** 6
Figure S4. CD spectrum of **4** 6
Figure S5. CD spectrum of **5** 7
Figure S6. CD spectrum of **6** 7
Figure S7. UV spectrum of **1** 8
Figure S8. UV spectrum of **2** 8
Figure S9. UV spectrum of **3** 9
Figure S10 UV spectrum of **4** 9
Figure S11. UV spectrum of **5** 10
Figure S12. UV spectrum of **6** 10
Figure S13. ^{1}H NMR spectrum (700 MHz, DMSO-d_6) of **1** 11
Figure S14. ^{13}C NMR spectrum (176 MHz, DMSO-d_6) of **1** 12
Figure S15. DEPT-135 NMR spectrum (176 MHz, DMSO-d_6) of **1** 13
Figure S16. COSY-45 spectrum (700 MHz, DMSO-d_6) of **1** 14
Figure S17. HSQC spectrum (700 MHz, DMSO-d_6) of **1** 15
Figure S18. HMBC spectrum (700 MHz, DMSO-d_6) of **1** 16
Figure S19. ROESY spectrum (700 MHz, DMSO-d_6) of **1** 17
Figure S20. ^{1}H NMR spectrum (700 MHz, $CDCl_3$) of **1** 18
Figure S21. ^{13}C NMR spectrum (176 MHz, $CDCl_3$) of **1** 19
Figure S22. HMBC spectrum (700 MHz, $CDCl_3$) of **1** 20
Figure S23. ^{1}H NMR spectrum (700 MHz, DMSO-d_6) of **2** 21
Figure S24. ^{13}C NMR spectrum (176 MHz, DMSO-d_6) of **2** 22
Figure S25. DEPT-135 NMR spectrum (176 MHz, DMSO-d_6) of **2** 23
Figure S26. COSY-45 spectrum (700 MHz, DMSO-d_6) of **2** 24
Figure S27. HSQC spectrum (700 MHz, DMSO-d_6) of **2** 25
Figure S28. HMBC spectrum (700 MHz, DMSO-d_6) of **2** 26
Figure S29. ROESY spectrum (700 MHz, DMSO-d6) of **2** 27
Figure S30. ^{1}H NMR spectrum (700 MHz, acetone-d_6) of **3** 28
Figure S31. ^{13}C NMR spectrum (75 MHz, acetone-d_6) of **3** 29
Figure S32. COSY-45 spectrum (700 MHz, acetone-d_6) of **3** 30
Figure S33. HSQC spectrum (700 MHz, acetone-d_6) of **3** 31
Figure S34. HMBC spectrum (700 MHz, acetone-d_6) of **3** 32
Figure S35. ROESY spectrum (700 MHz, acetone-d_6) of **3** 33
Figure S36. ^{1}H NMR spectrum (500 MHz, DMSO-d_6) of **4** 34
Figure S37. ^{13}C NMR spectrum (125 MHz, DMSO-d_6) of **4** 35
Figure S38. DEPT-135 NMR spectrum (125 MHz, DMSO-d_6) of **4** 36
Figure S39. COSY-45 spectrum (500 MHz, DMSO-d_6) of **4** 37
Figure S40. HSQC spectrum (500 MHz, DMSO-d_6) of**4** 38
Figure S41. HMBC spectrum (500 MHz, DMSO-d_6) of **4** 39

Figure S42. ROESY spectrum (500 MHz, DMSO-d_6) of **4**....40
Figure S43. ^{1}H NMR spectrum (500 MHz, DMSO-d_6) of **5**....41
Figure S44. ^{13}C NMR spectrum (125 MHz, DMSO-d_6) of **5**....42
Figure S45. DEPT-135 NMR spectrum (125 MHz, DMSO-d_6) of **5**....43
Figure S46. COSY-45 spectrum (500 MHz, DMSO-d_6) of **5**....44
Figure S47. HSQC spectrum (500 MHz, DMSO-d_6) of **5**....45
Figure S48. HMBC spectrum (500 MHz, DMSO-d_6) of **5**....46
Figure S49. ROESY spectrum (500 MHz, DMSO-d_6) of **5**....47
Figure S50. ^{1}H NMR spectrum (500 MHz, $CDCl_3$) of **6**....48
Figure S51. ^{13}C NMR spectrum (125 MHz, $CDCl_3$) of **6**....49
Figure S52. DEPT-135 NMR spectrum (125 MHz, $CDCl_3$) of **6**....50
Figure S53. COSY-45 spectrum (500 MHz, $CDCl_3$) of **6**....51
Figure S54. HSQC spectrum (500 MHz, $CDCl_3$) of **6**....52
Figure S55. HMBC spectrum (500 MHz, $CDCl_3$) of **6**....53
Figure S56. NOESY spectrum (500 MHz, $CDCl_3$) of **6**....54
Figure S57. ^{1}H NMR spectrum (500 MHz, acetone-d_6) of **1a**....55
Figure S58. ^{13}C NMR spectrum (125 MHz, acetone-d_6) of **1a**....56
Figure S59. ^{1}H NMR spectrum (500 MHz, acetone-d_6) of **1a-1**....57
Figure S60. COSY-45 spectrum (500 MHz, acetone-d_6) of **1a-1**....58
Figure S61. ^{1}H NMR spectrum (500 MHz, acetone-d_6) of **1a-2**....59
Figure S62. COSY-45 spectrum (500 MHz, acetone-d_6) of **1a-2**....60
Figure S63. ^{1}H NMR spectrum (700 MHz, acetone-d_6) of **4a**....61
Figure S64. ^{13}C NMR spectrum (125 MHz, acetone-d_6) of **4a**....62
Figure S65. Viability of the cells exposed to the investigated compounds for 72 h. Viability was measured using MTT assay and normalized to control....63
Figure S66. Microphotographies of the cells exposed to the investigated compounds for 72 h. Viability was measured using MTT assay and normalized to control....63

Experimental Section

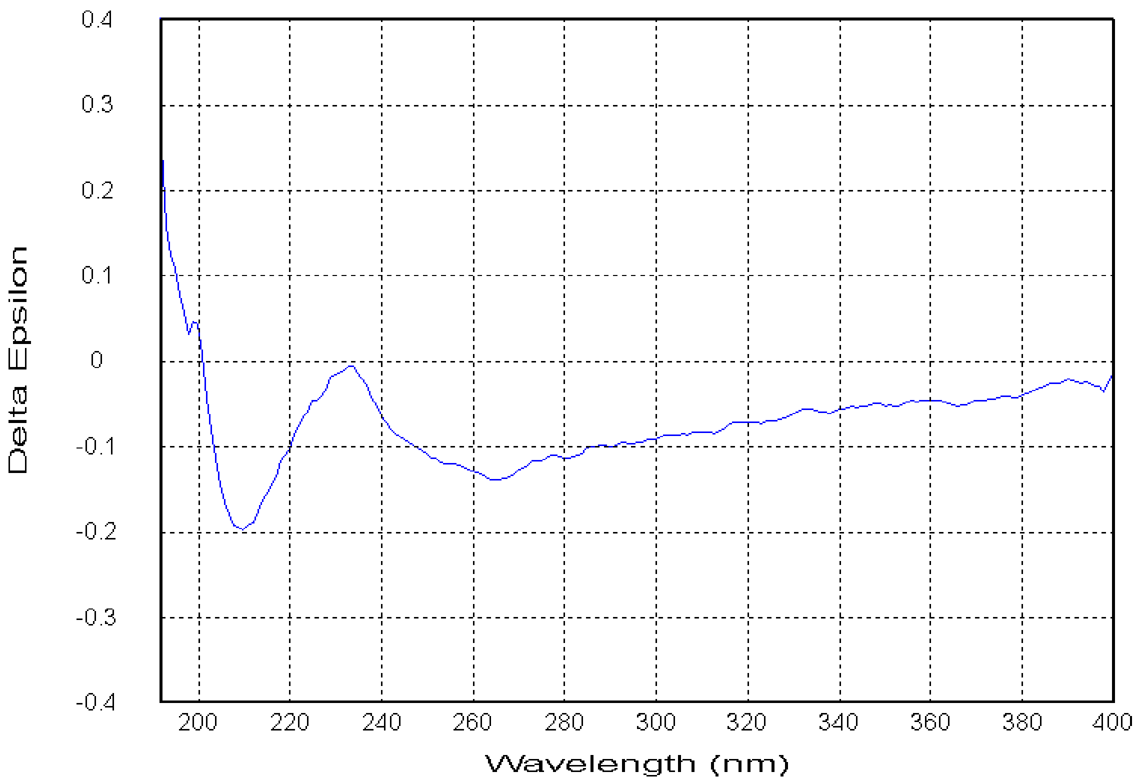

Figure S1. CD spectrum of **1**

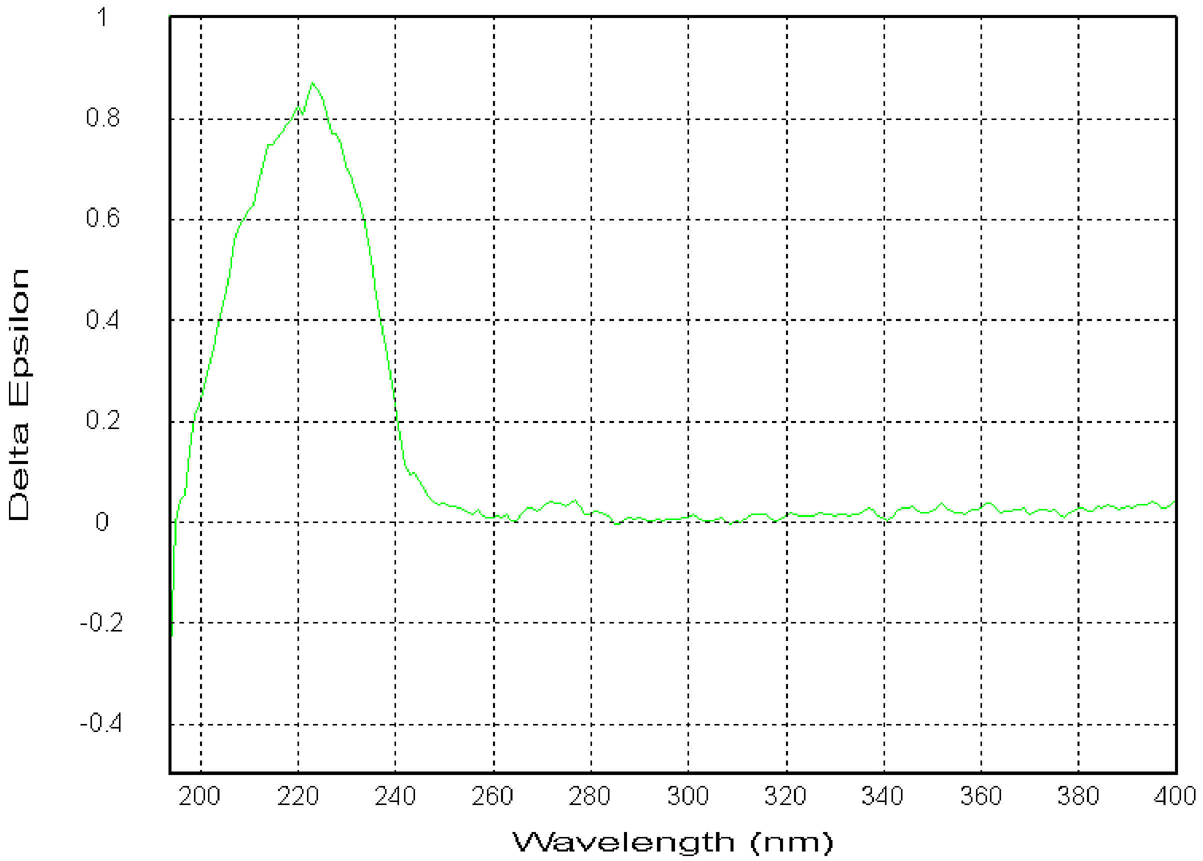

Figure S2. CD spectrum of **2**

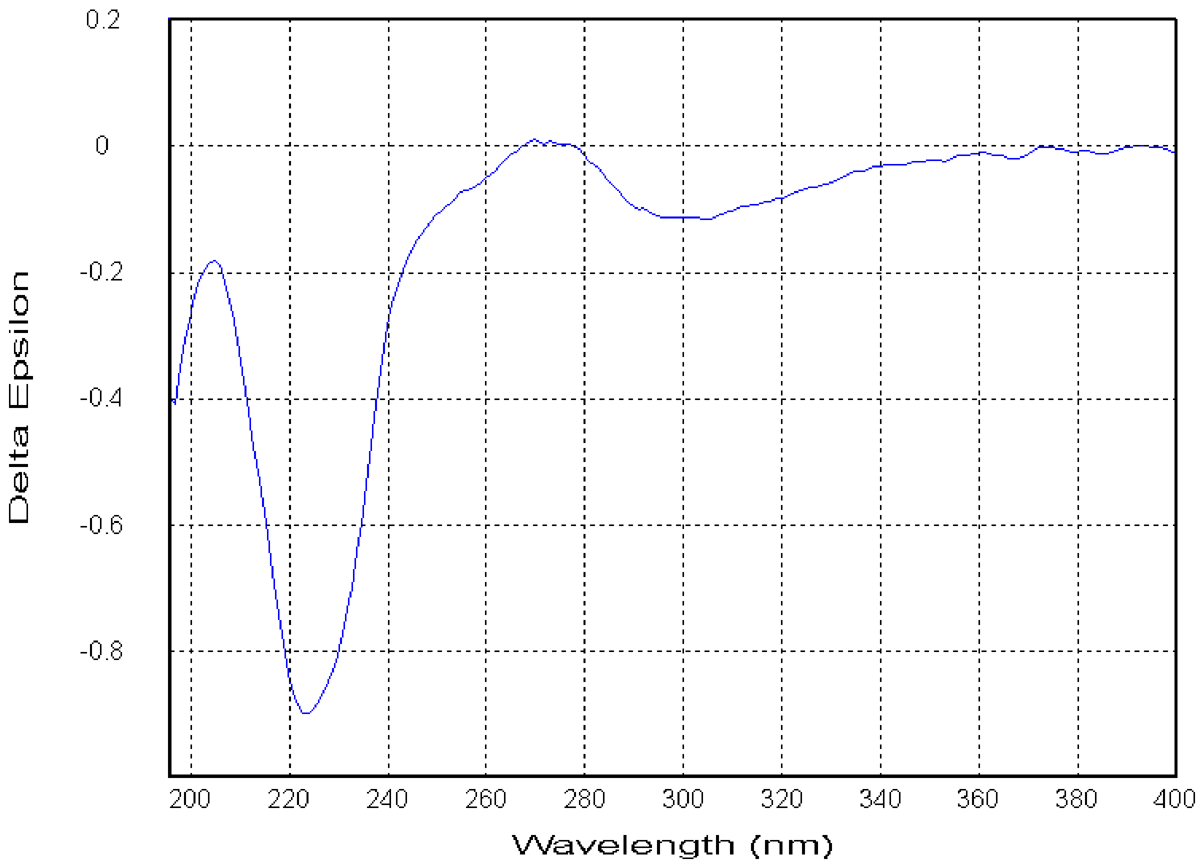

Figure S3. CD spectrum of **3**

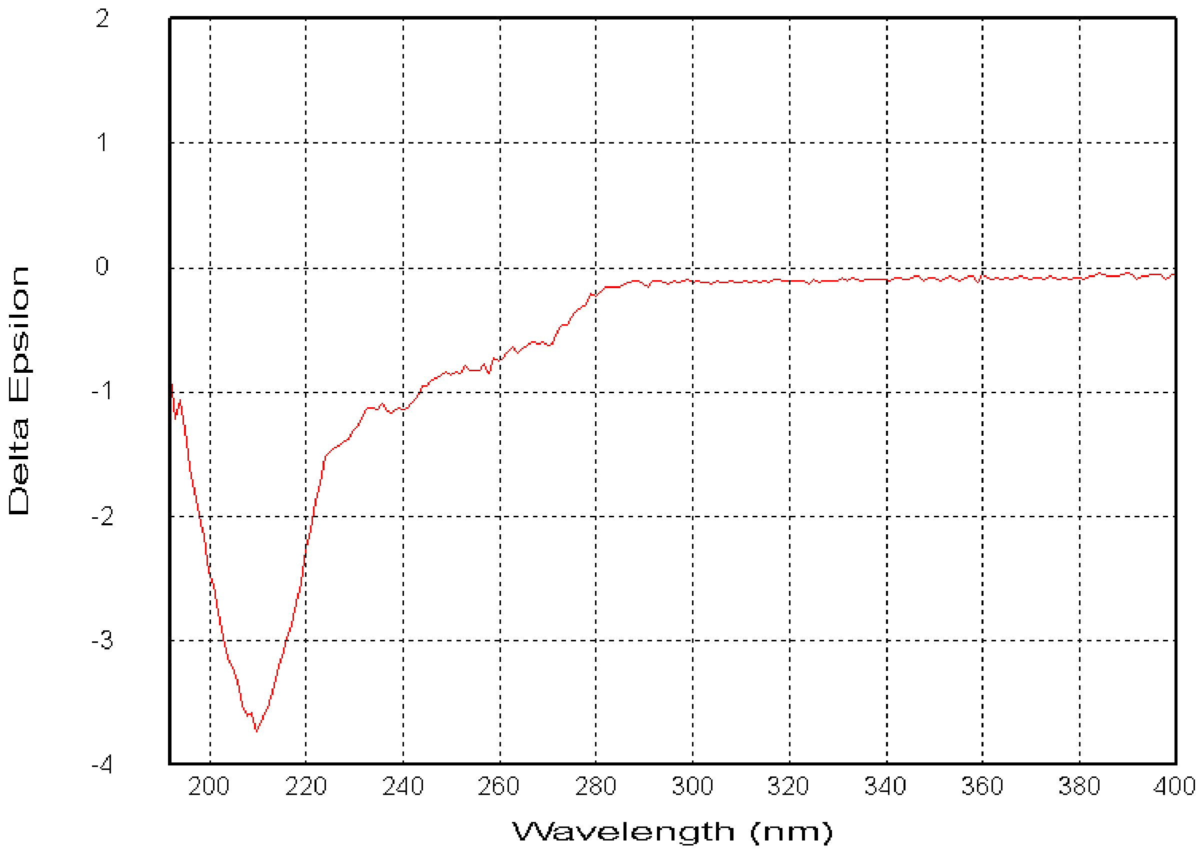

Figure S4. CD spectrum of **4**

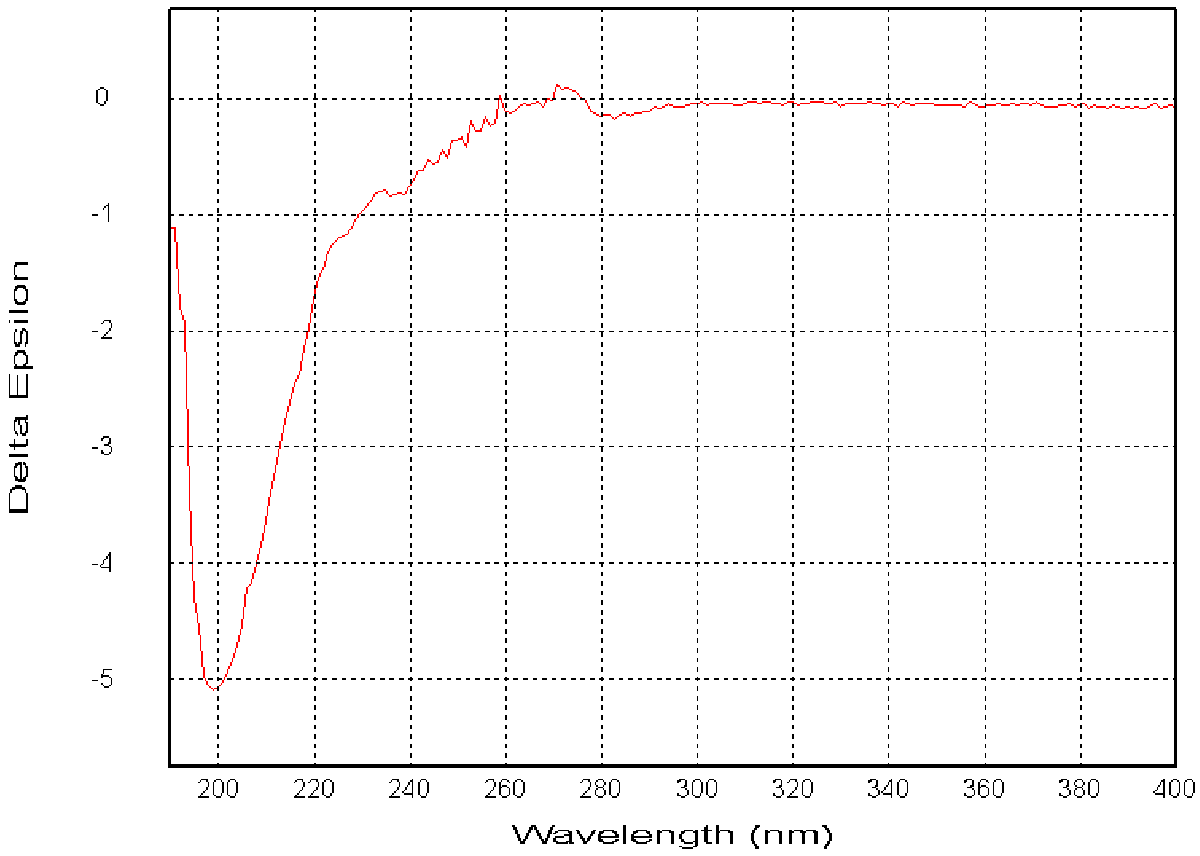

Figure S5. CD spectrum of **5**

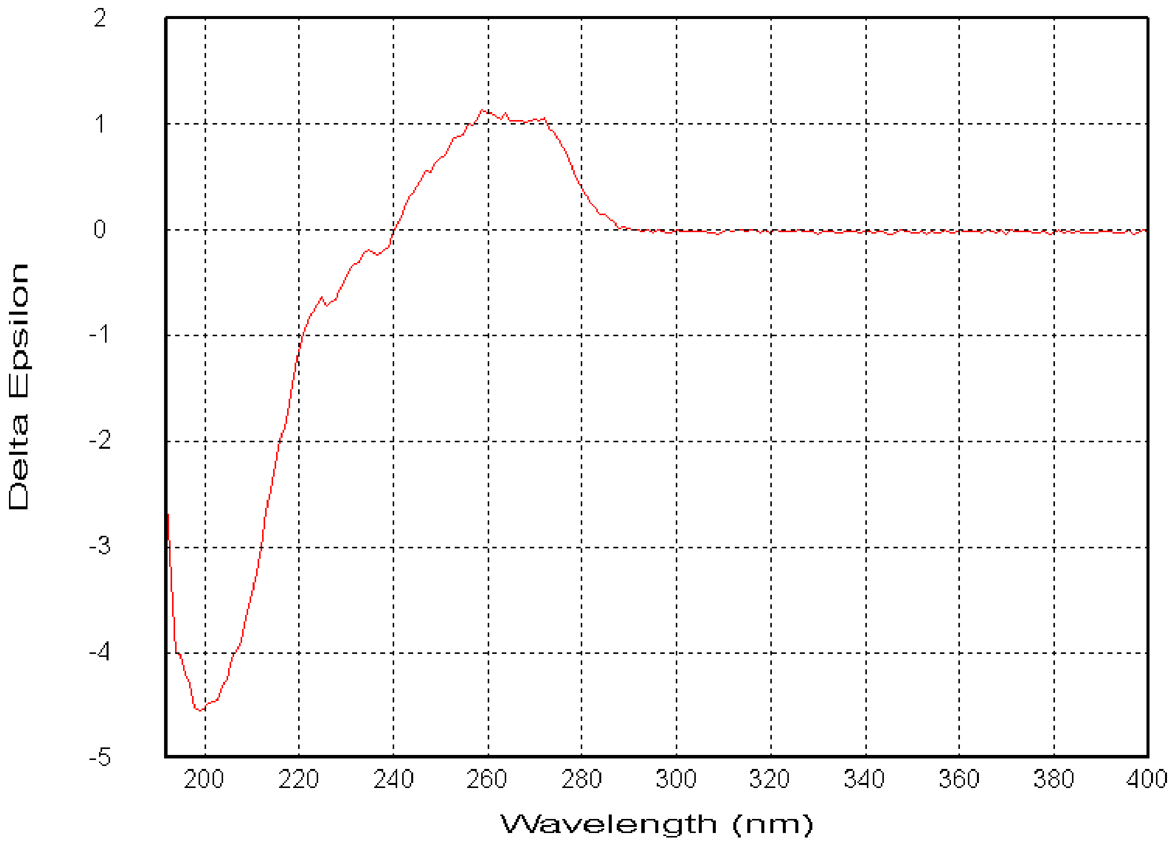

Figure S6. CD spectrum of **6**

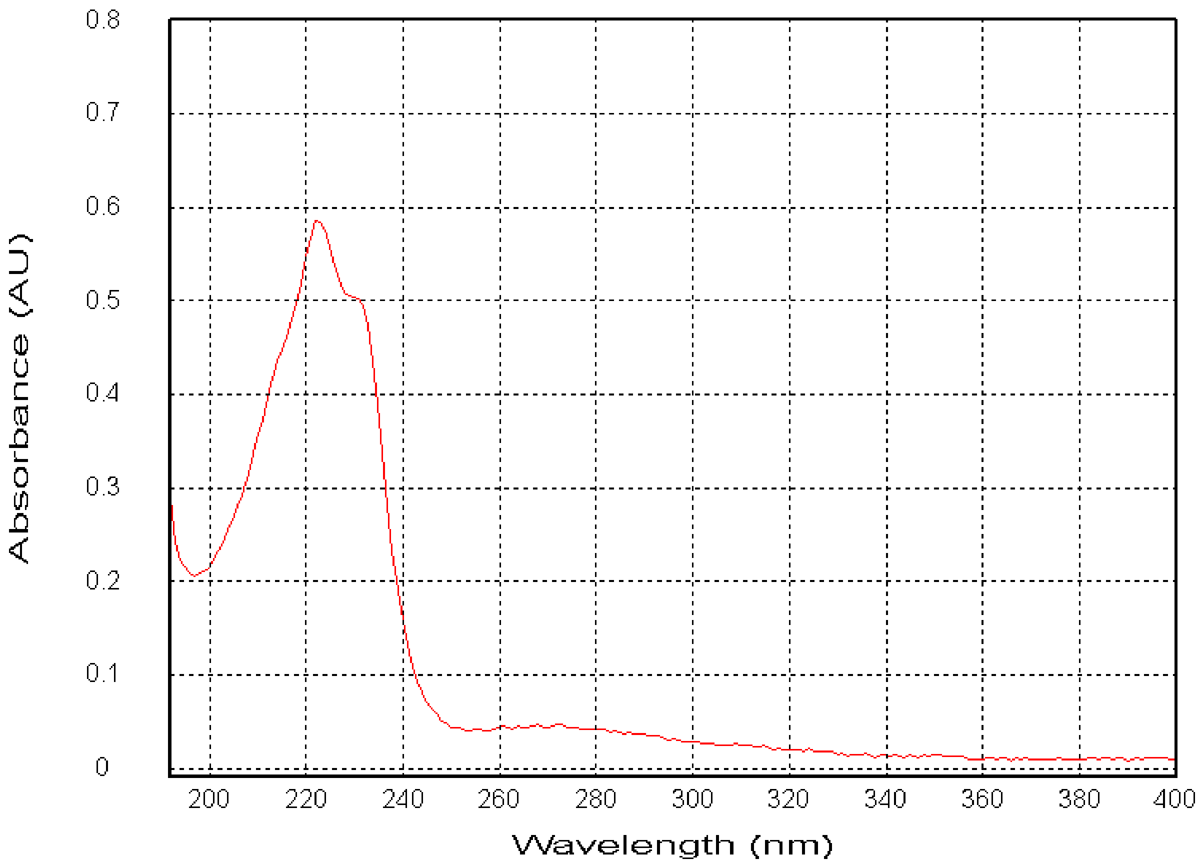

Figure S7. UV spectrum of **1**

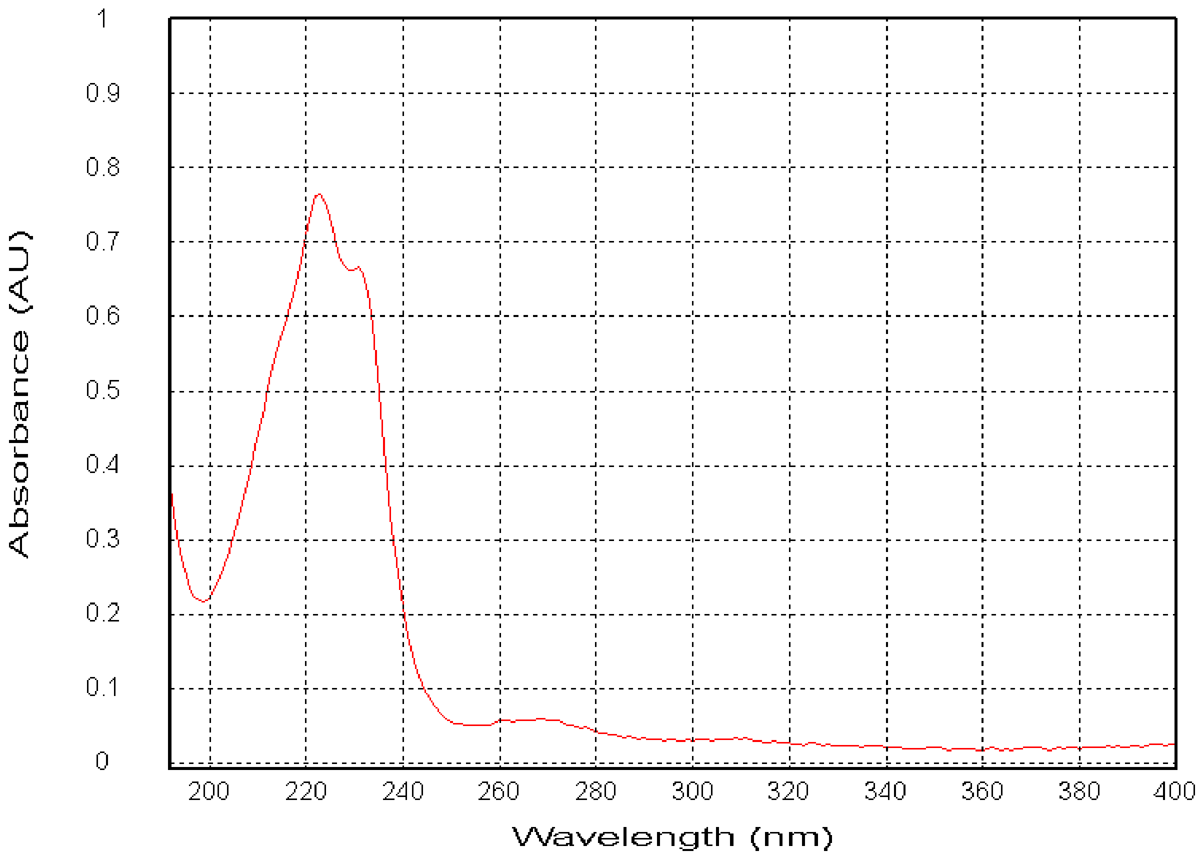

Figure S8. UV spectrum of **2**

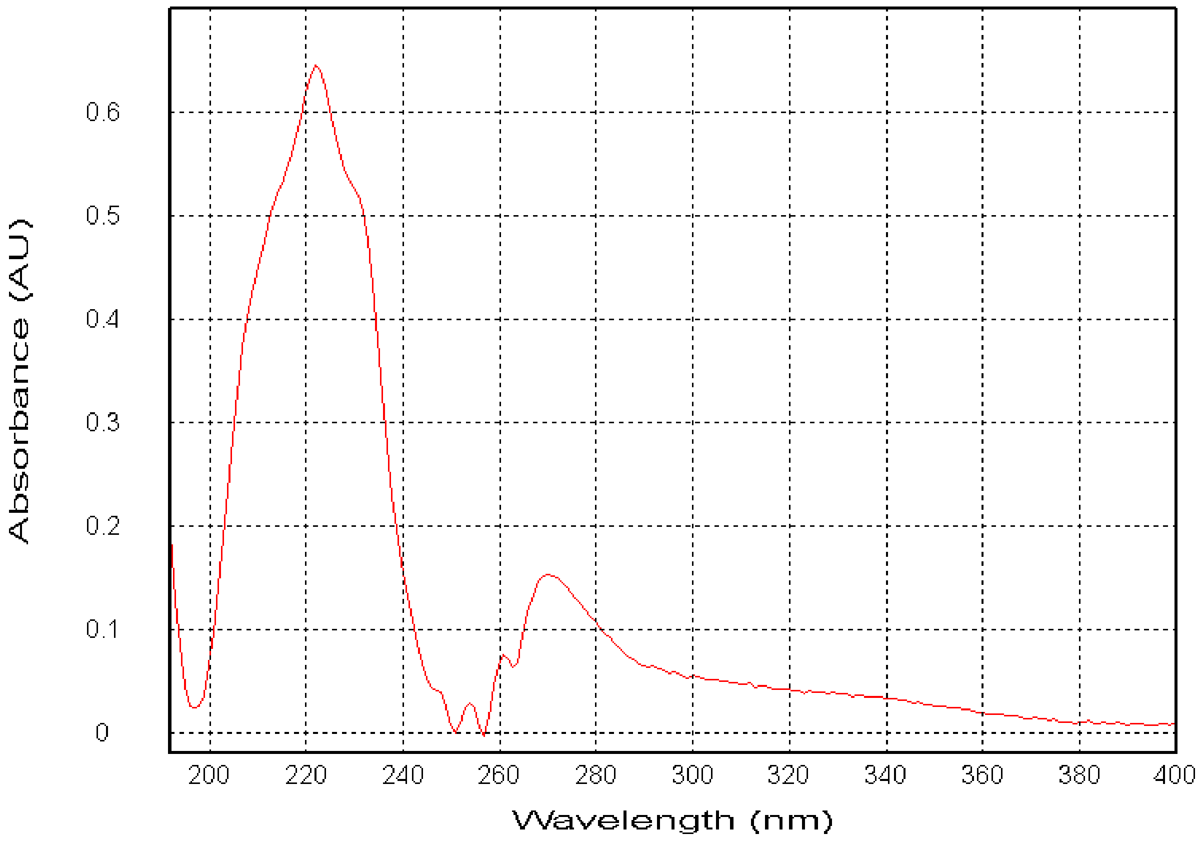

Figure S9. UV spectrum of **3**

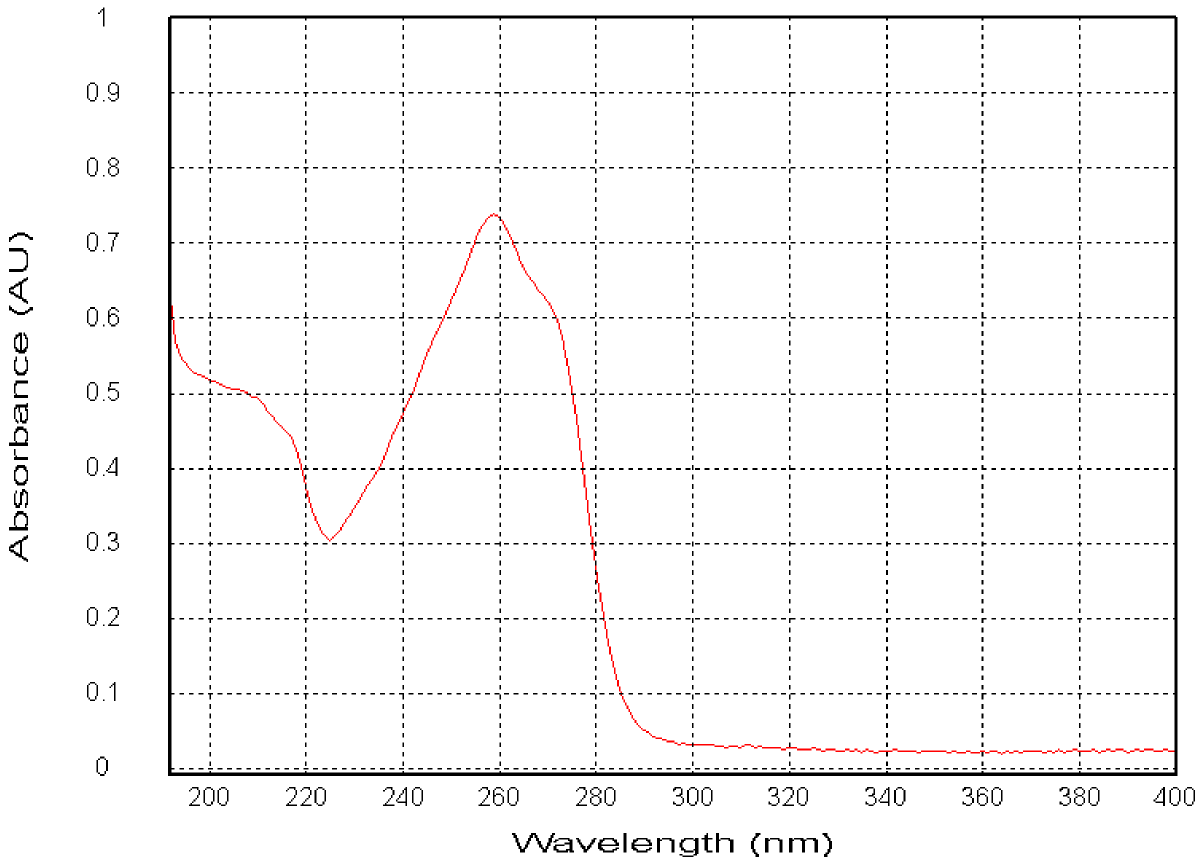

Figure S10. UV spectrum of **4**

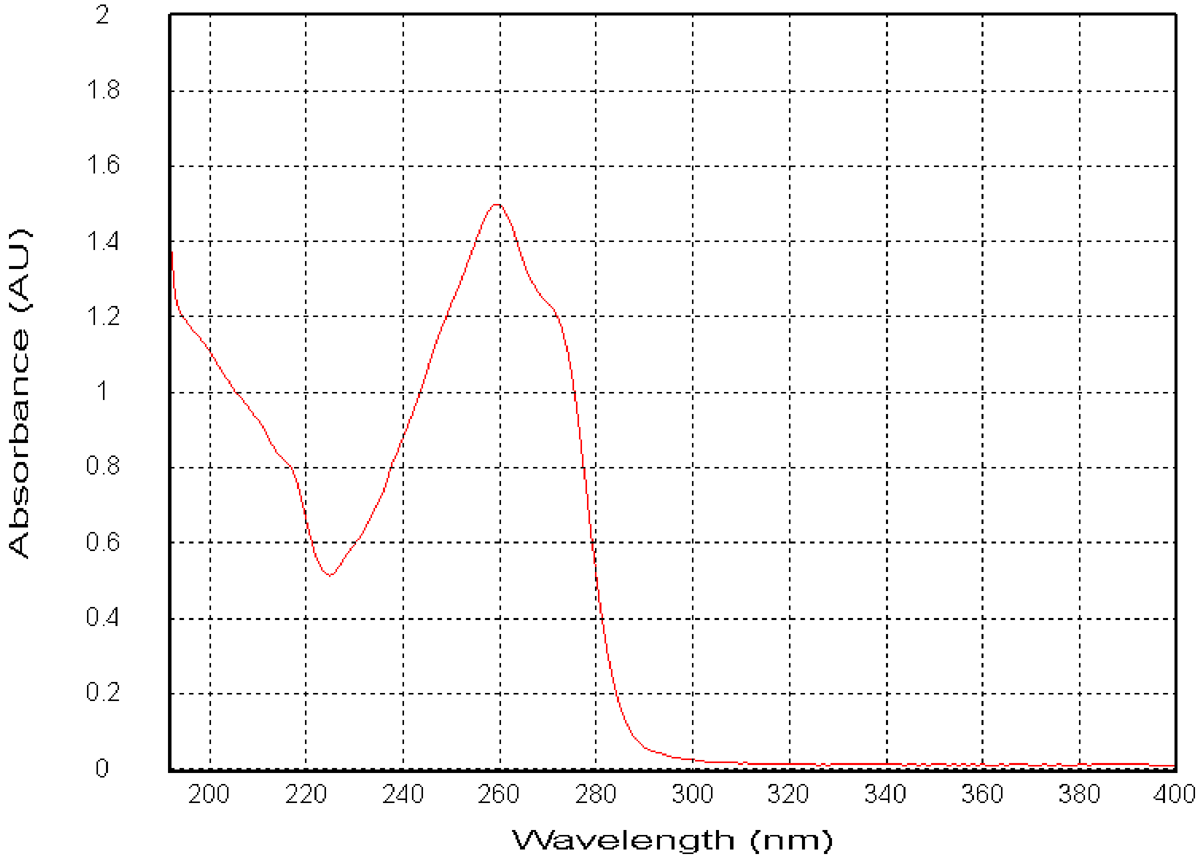

Figure S11. UV spectrum of **5**

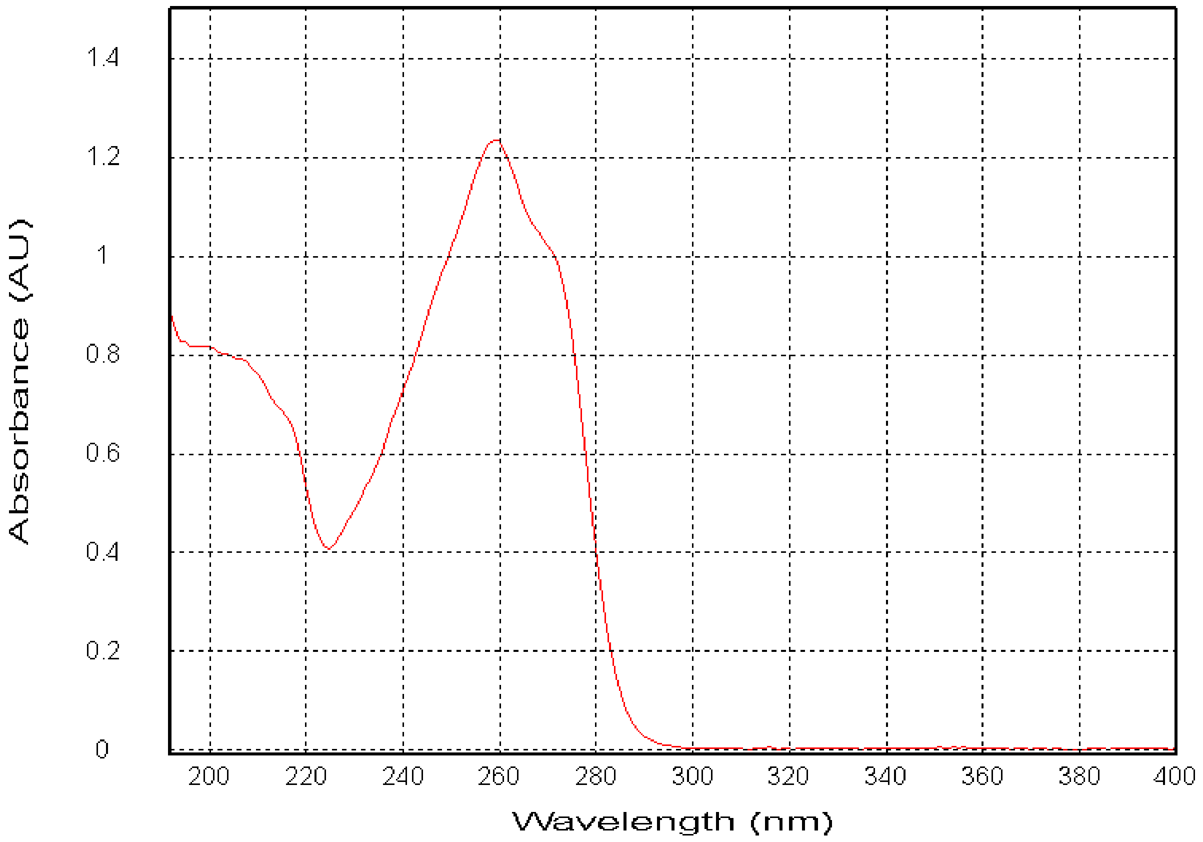

Figure S12. UV spectrum of **6**

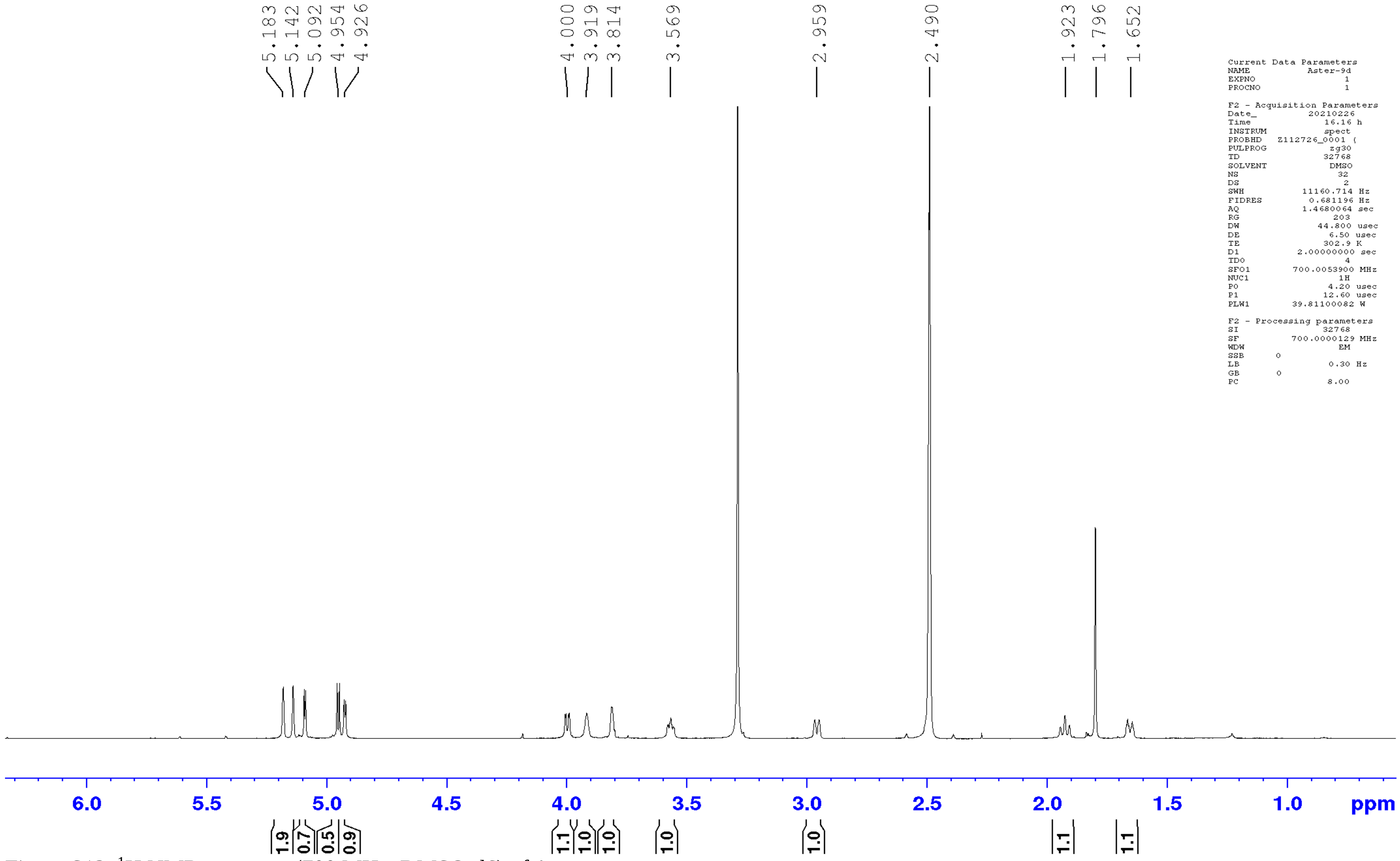

Figure S13. ^{1}H NMR spectrum (700 MHz, DMSO-d6) of **1**

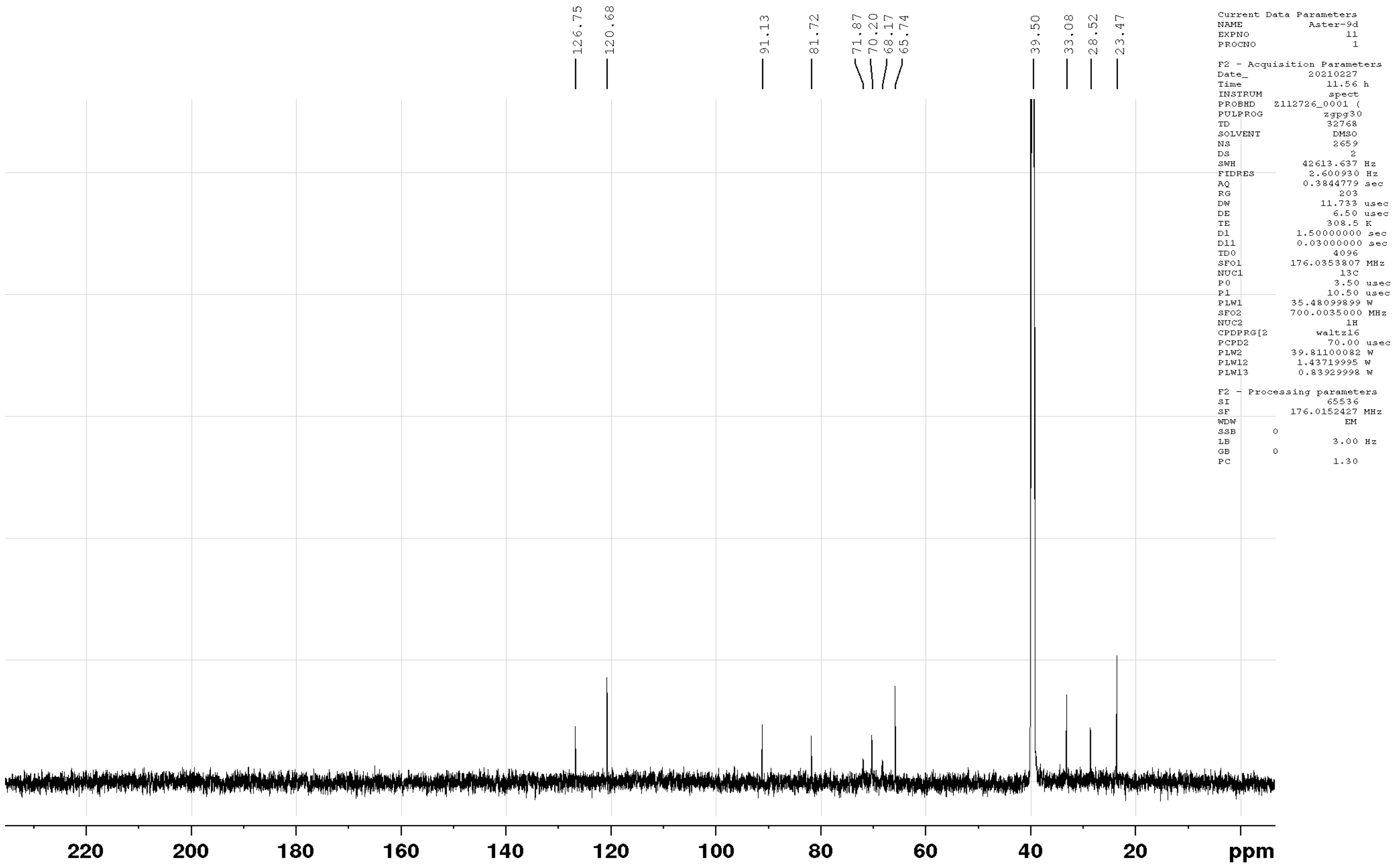

Figure S14. ^{13}C NMR spectrum (176 MHz, DMSO-d6) of **1**

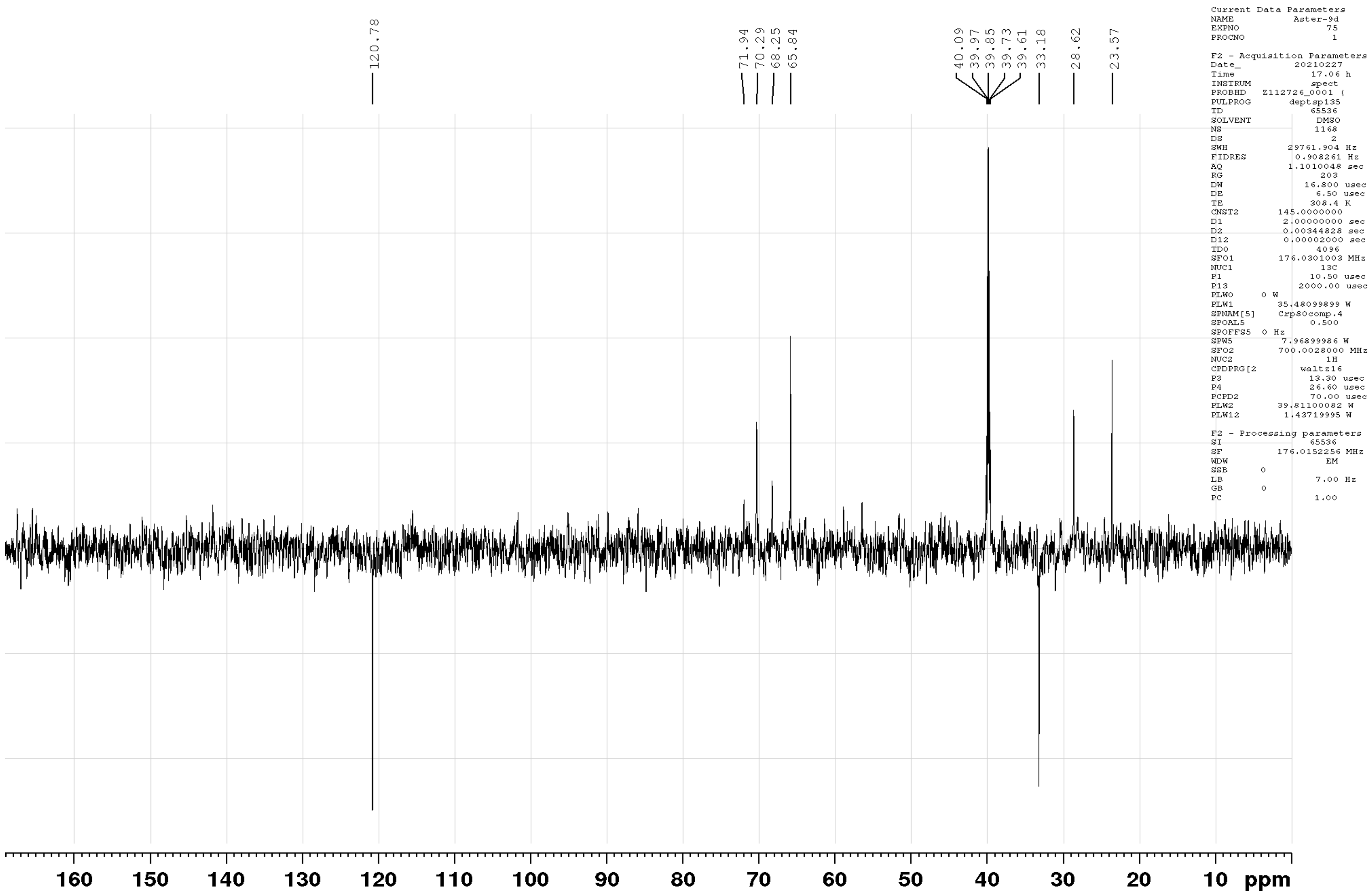

Figure S15. DEPT-135 NMR spectrum (176 MHz, DMSO-d6) of **1**

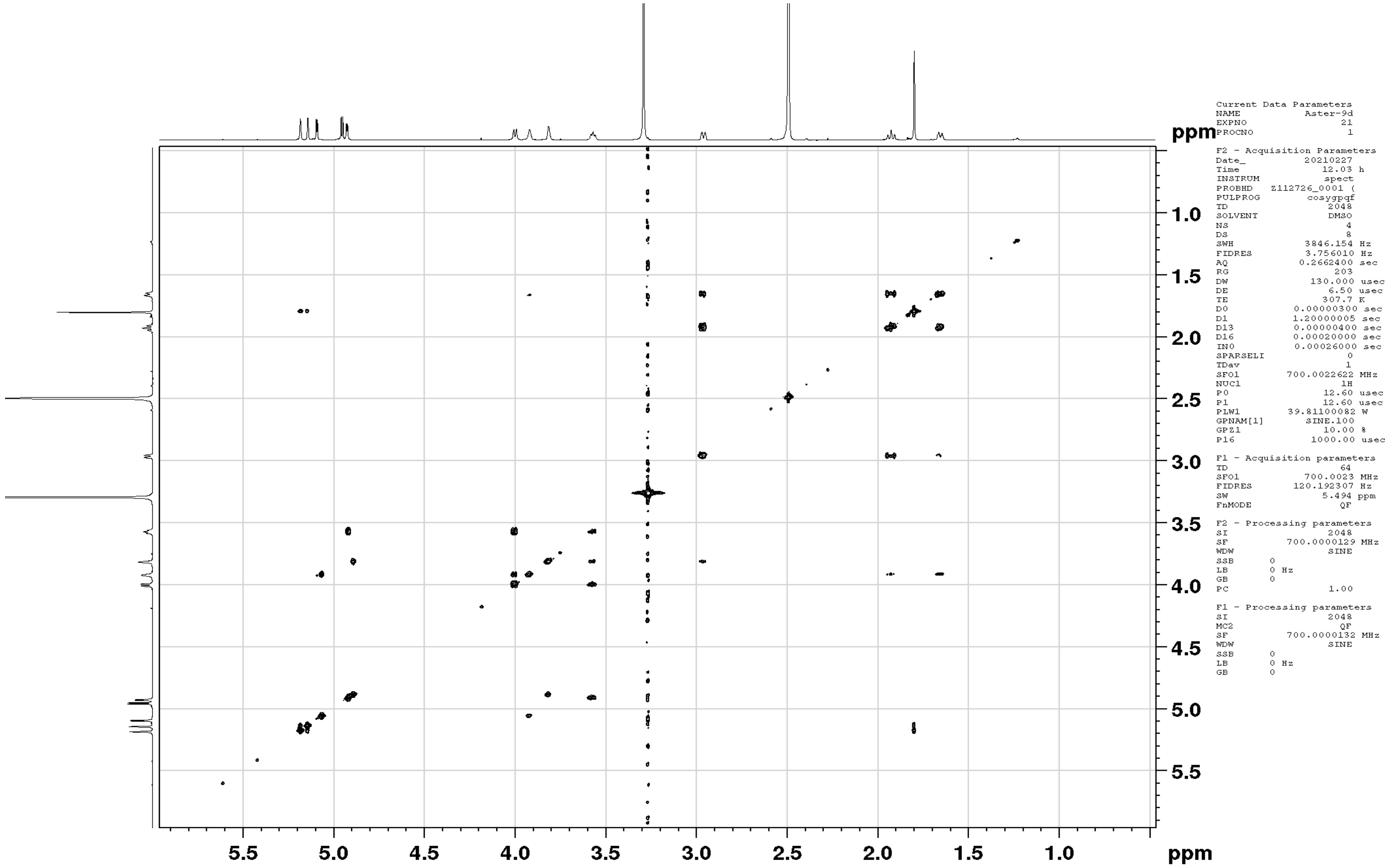

Figure S16. COSY-45 spectrum (700 MHz, DMSO-d6) of **1**

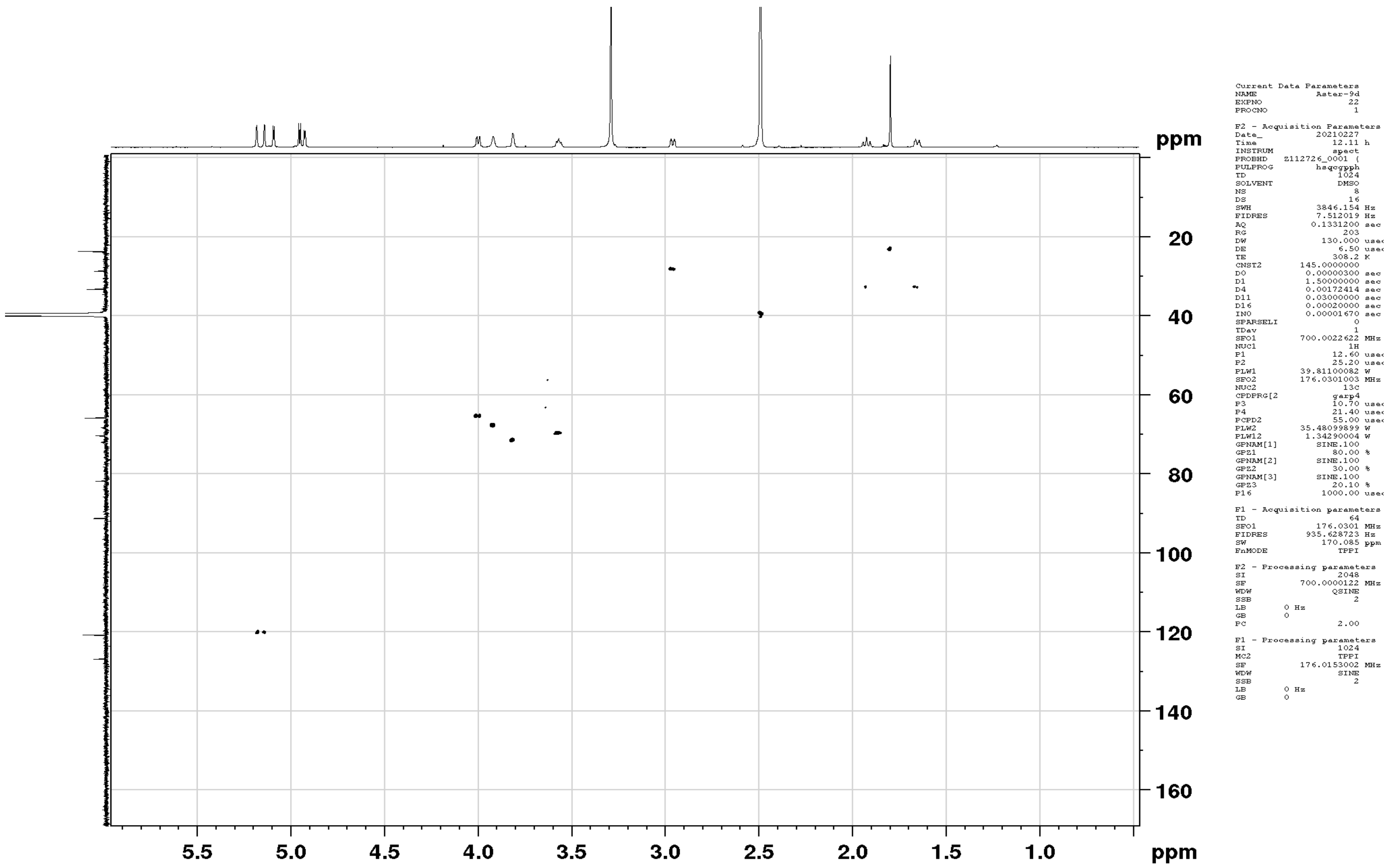

Figure S17. HSQC spectrum (700 MHz, DMSO-d6) of **1**

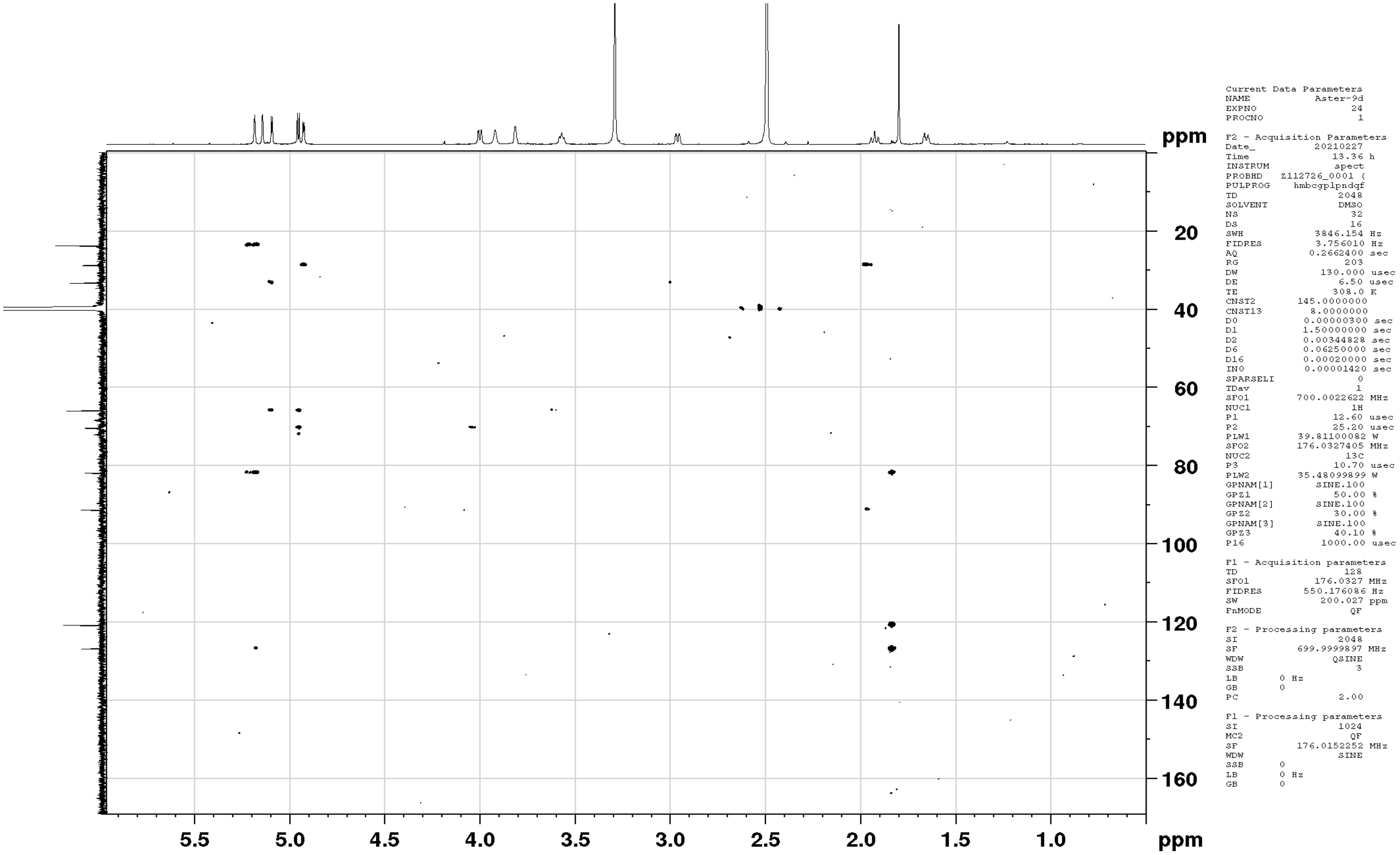

Figure S18. HMBC spectrum (700 MHz, DMSO-d6) of **1**

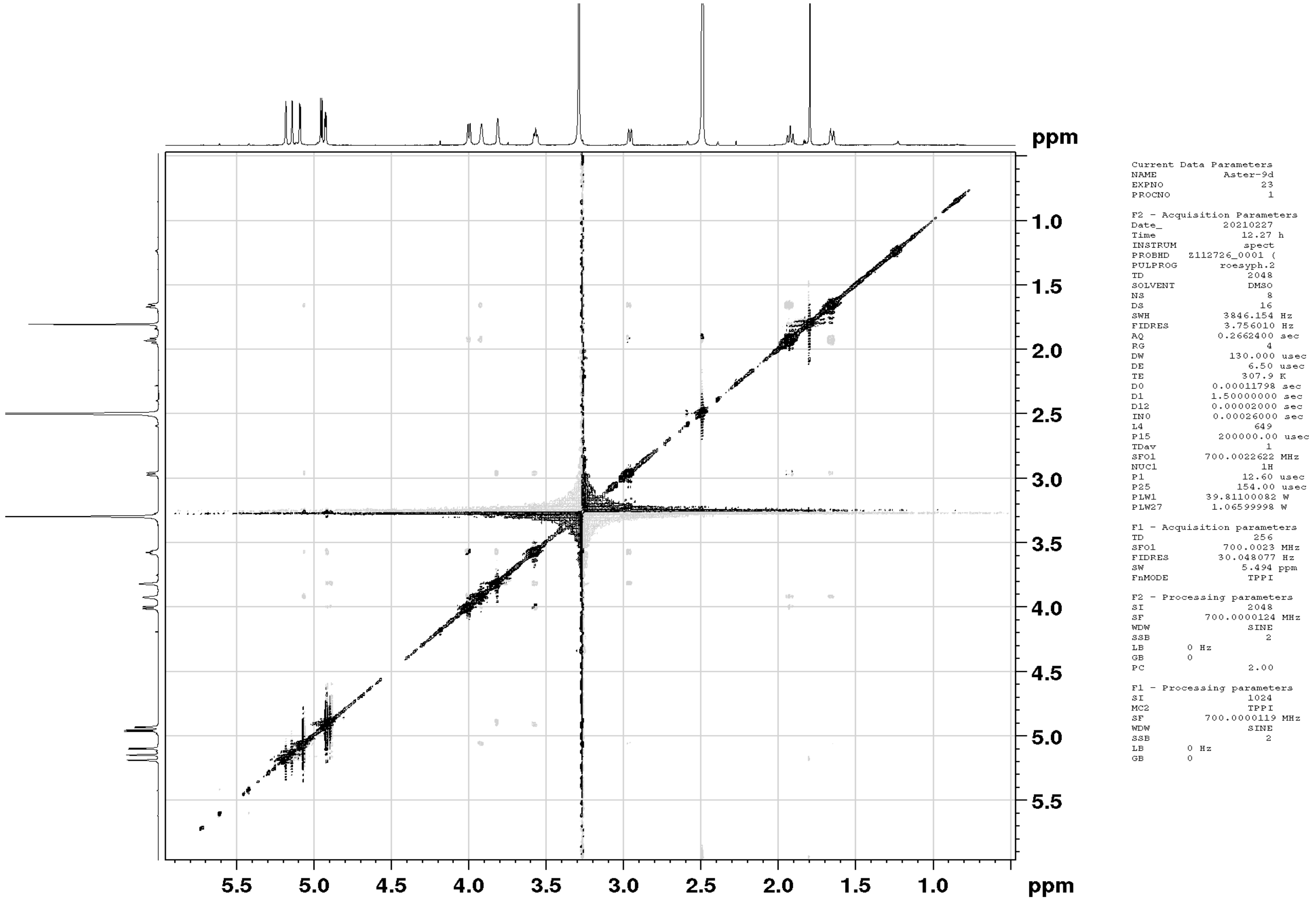

Figure S19. ROESY spectrum (700 MHz, DMSO-d6) of **1**

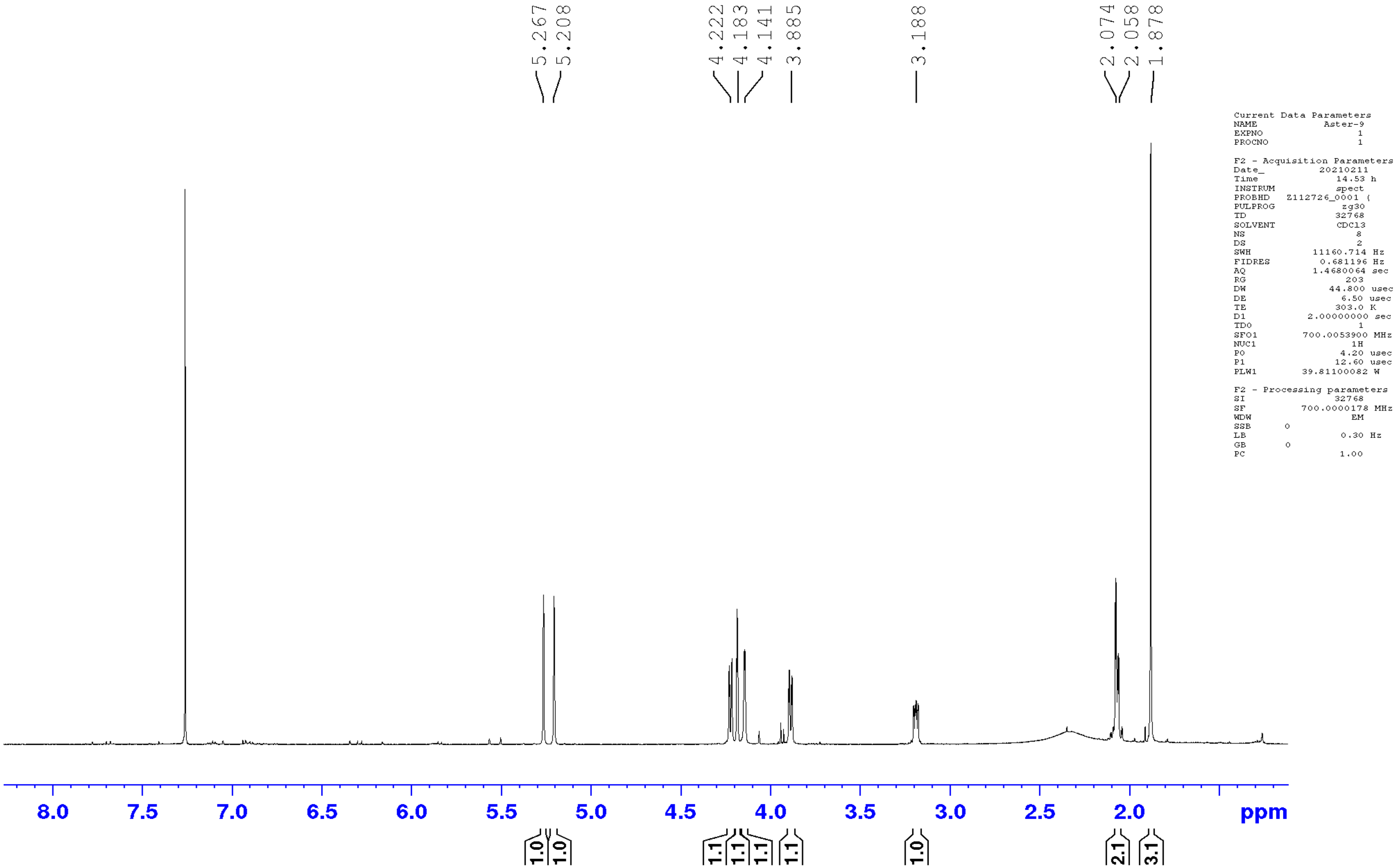

Figure S20. ^{1}H NMR spectrum (700 MHz, $CDCl_3$) of **1**

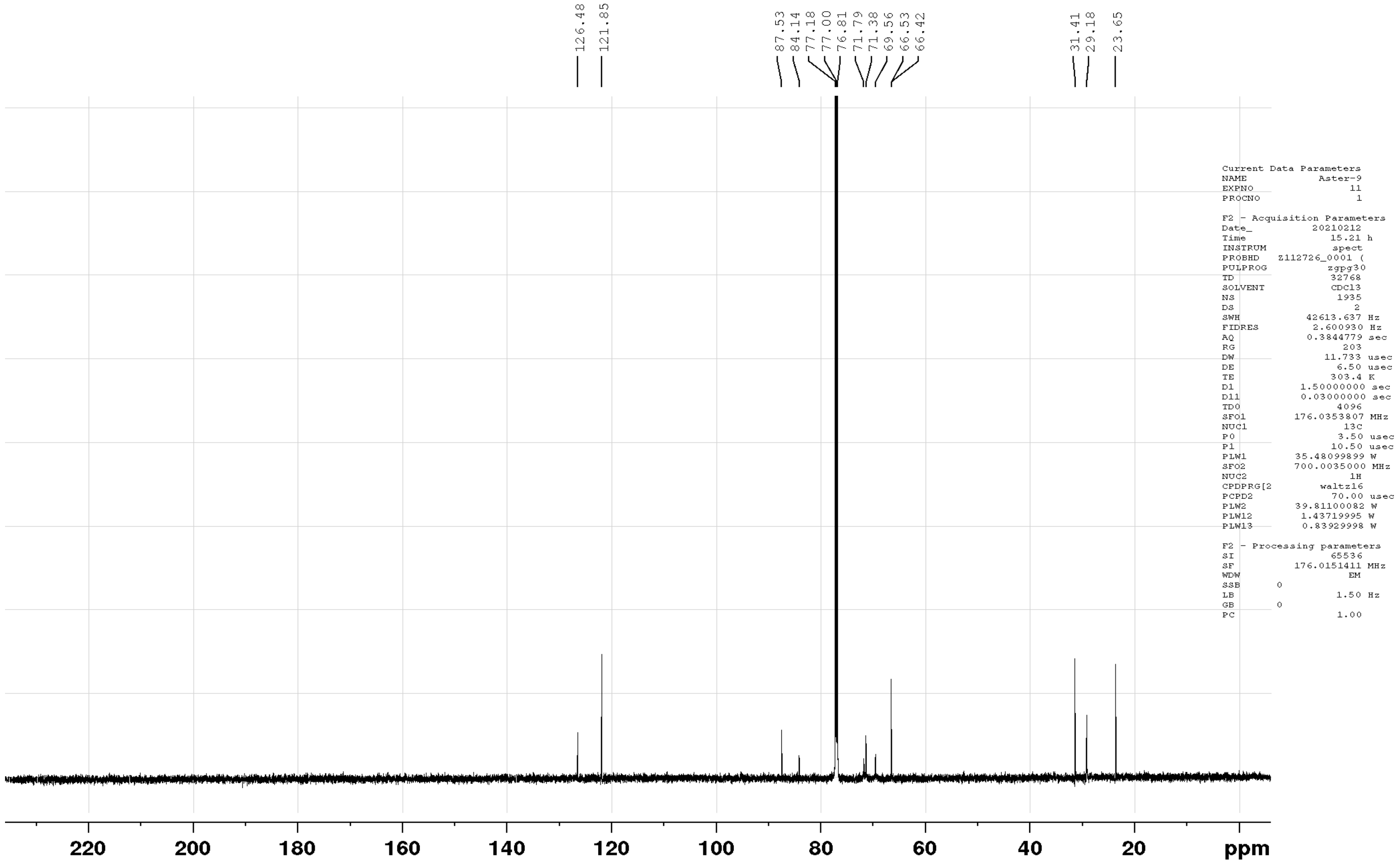

Figure S21. ^{13}C NMR spectrum (176 MHz, $CDCl_3$) of **1**

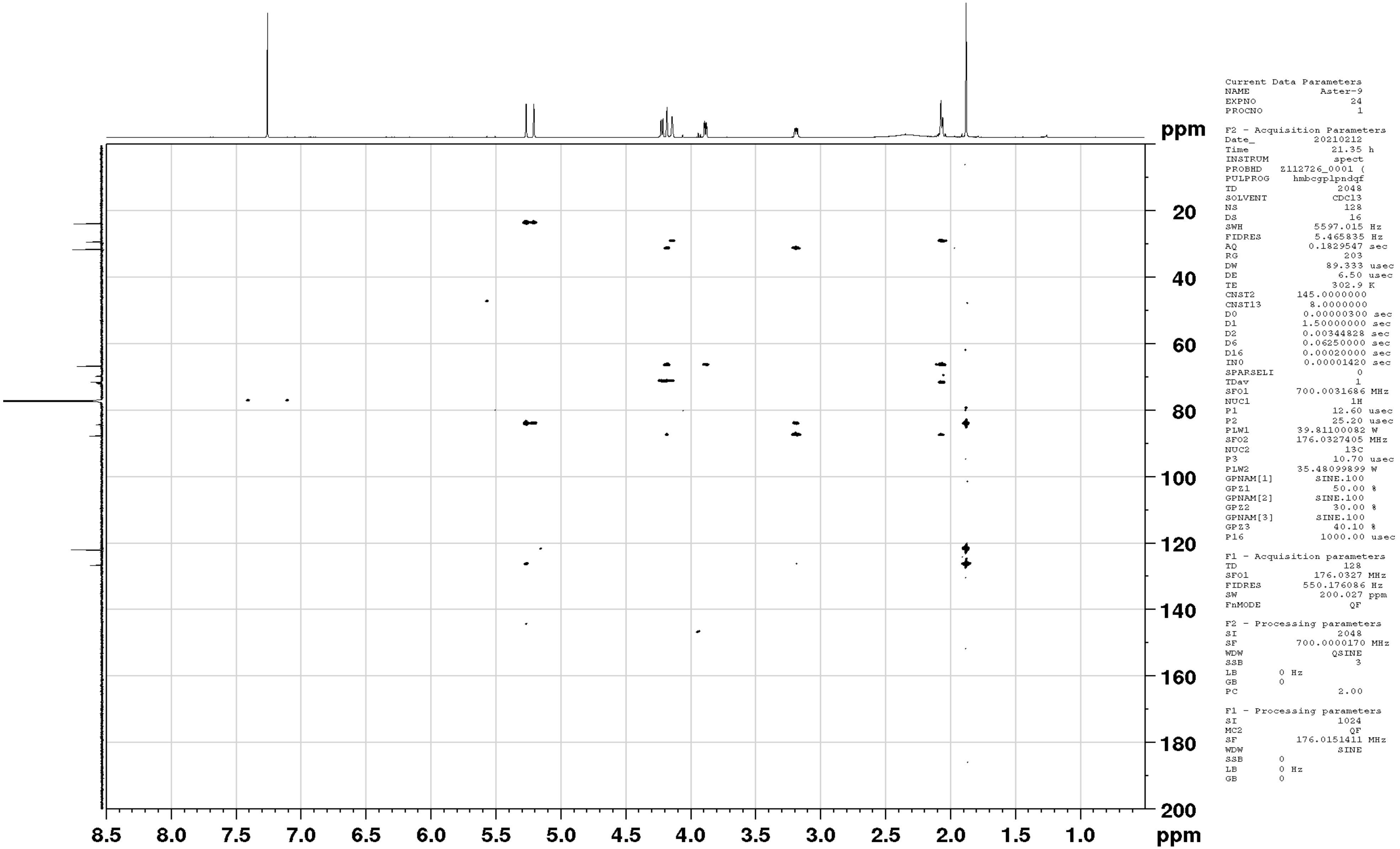

Figure S22. HMBC spectrum (700 MHz, $CDCl_3$) of **1**

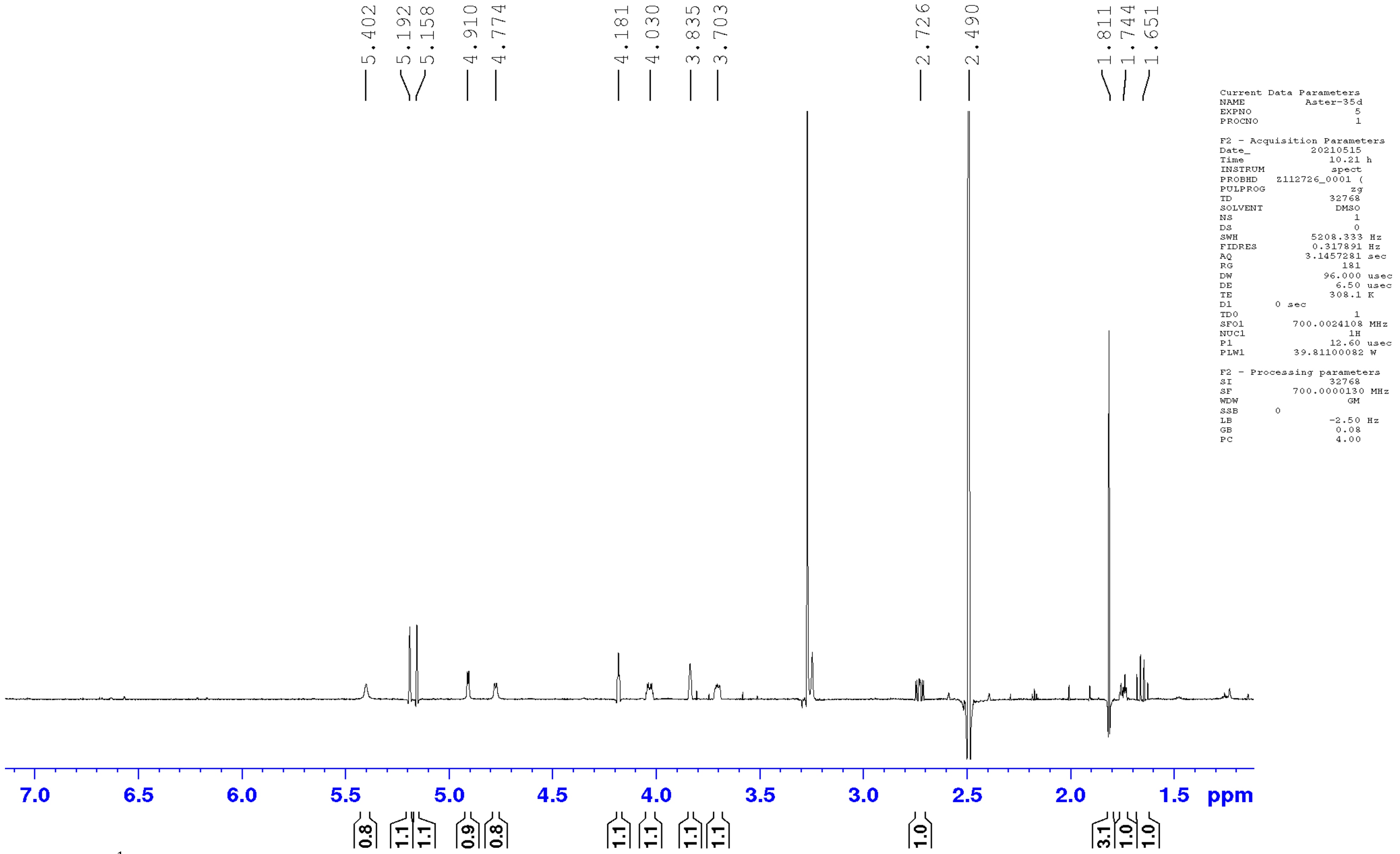

Figure S23. ^{1}H NMR spectrum (700 MHz, DMSO-d6) of **2**

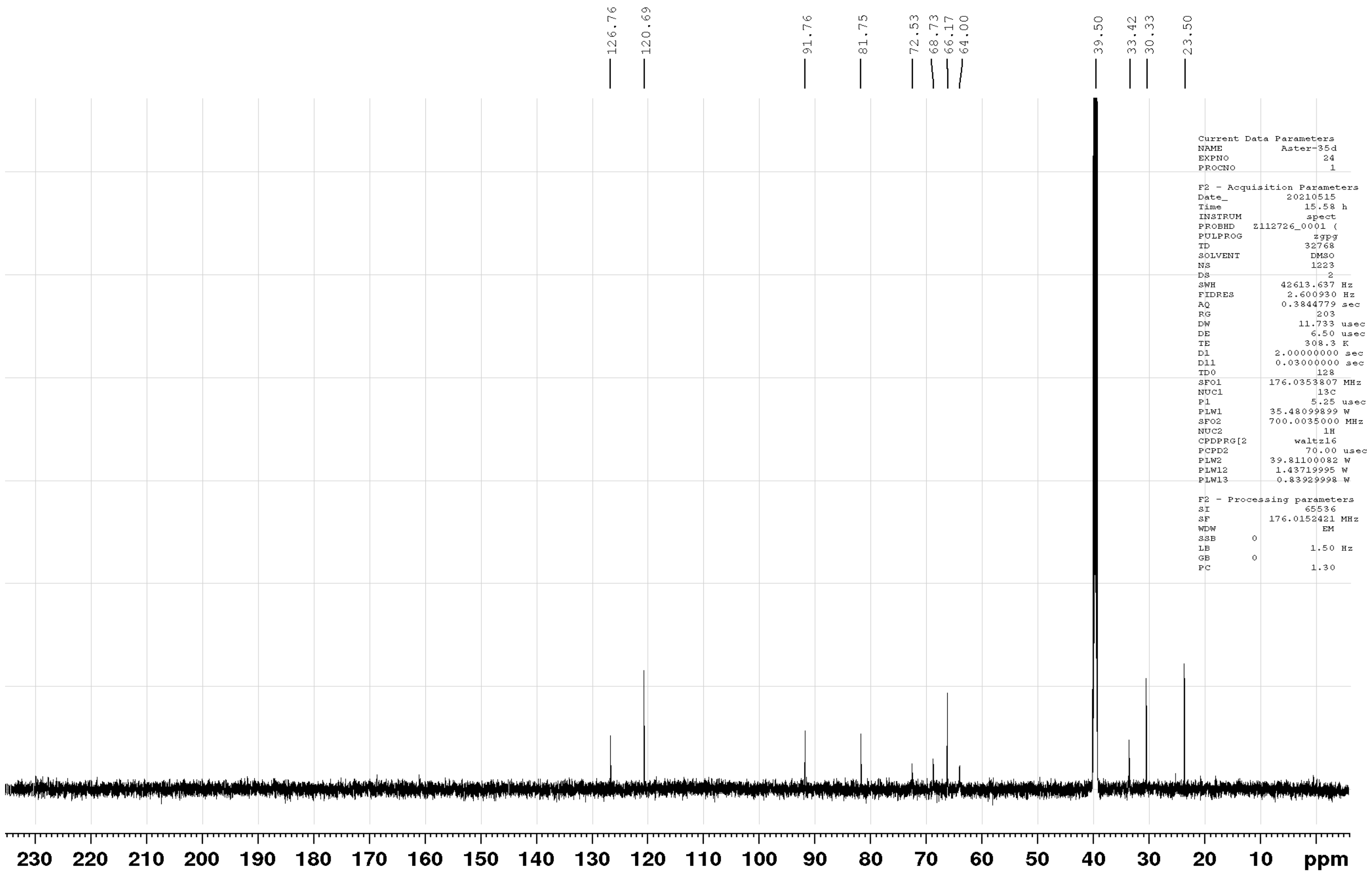

Figure S24. ^{13}C NMR spectrum (176 MHz, DMSO-d6) of **2**

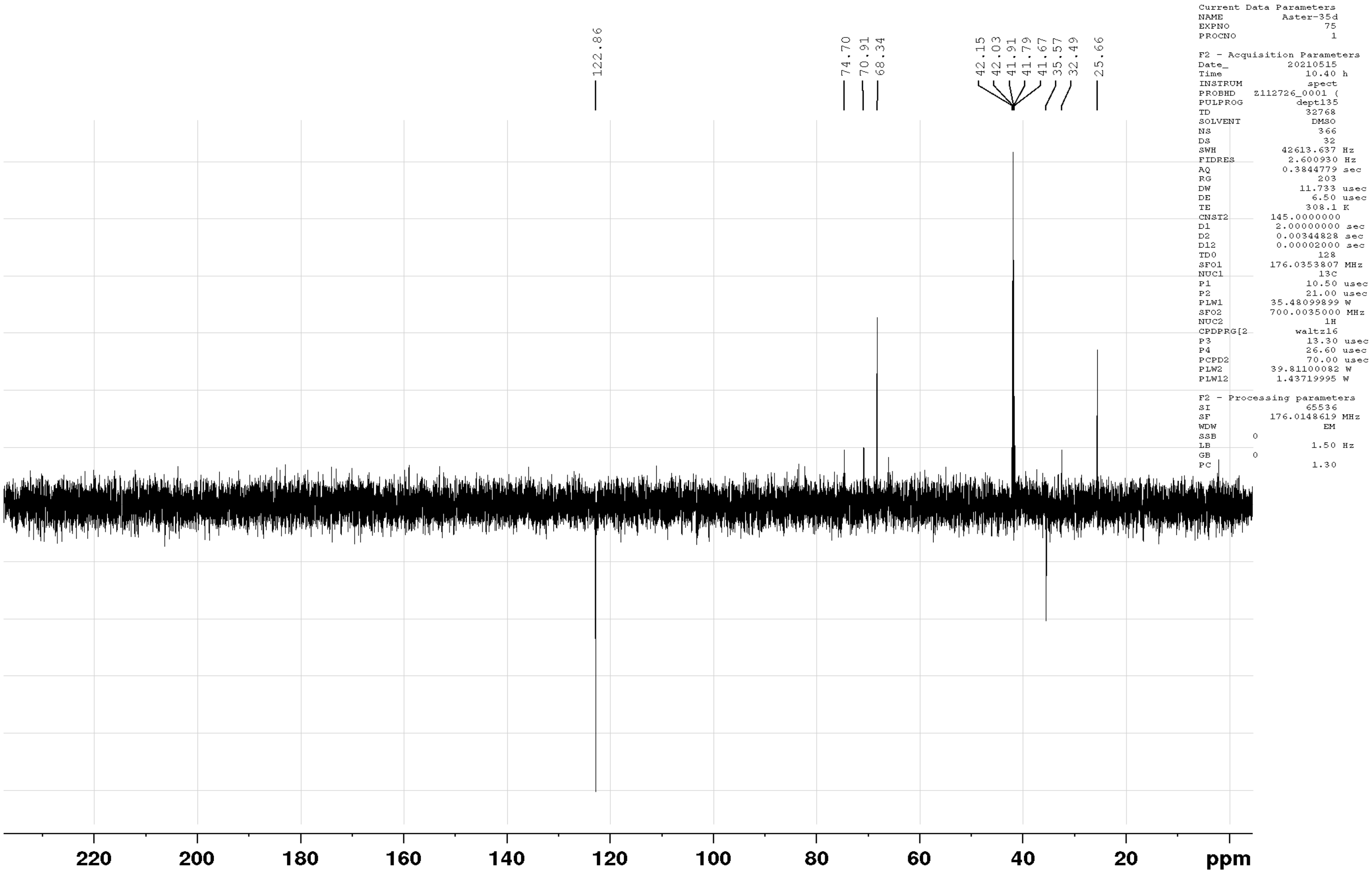

Figure S25. DEPT-135 NMR spectrum (176 MHz, DMSO-d6) of **2**

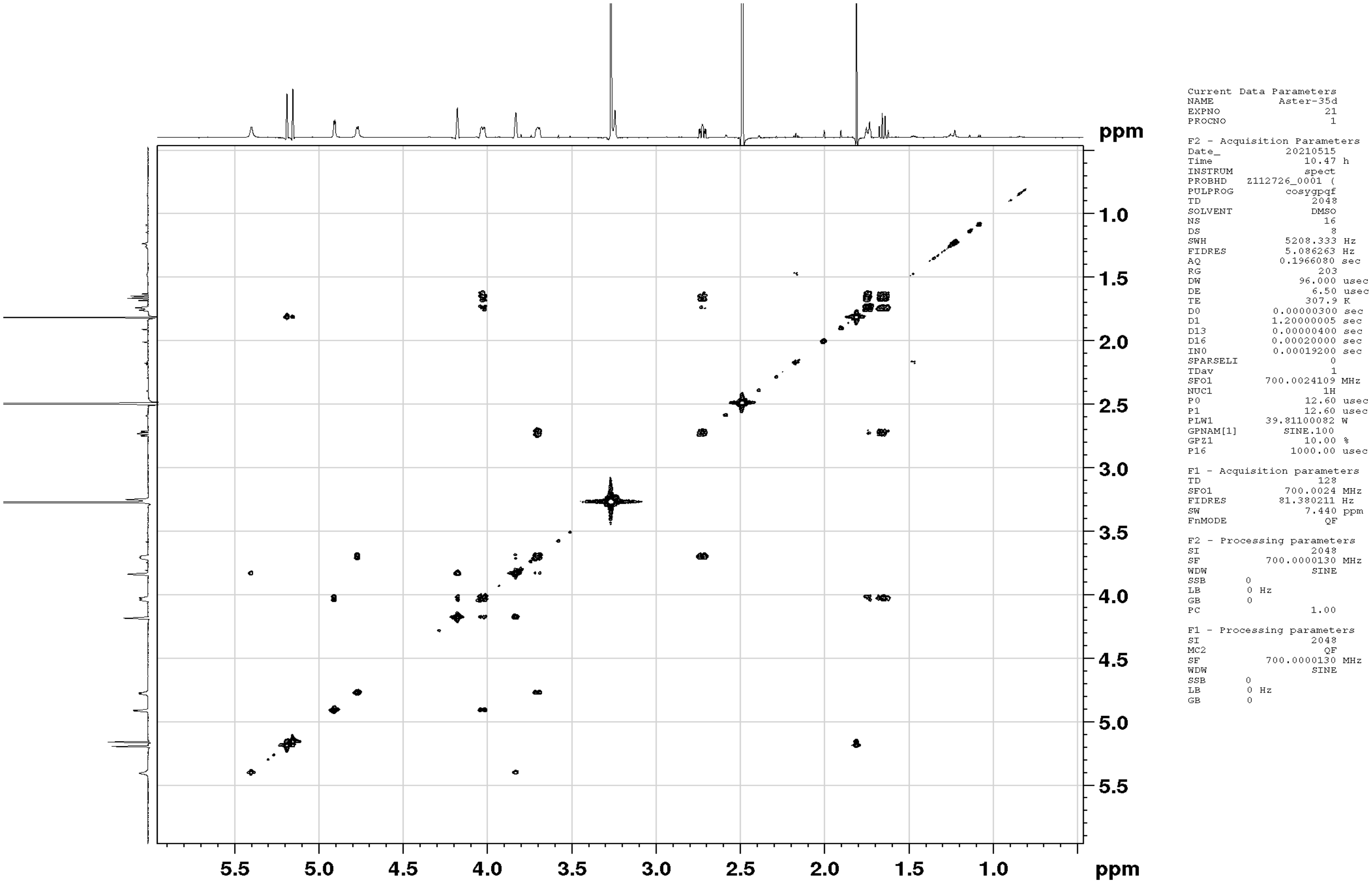

Figure S26. COSY-45 spectrum (700 MHz, DMSO-d6) of **2**

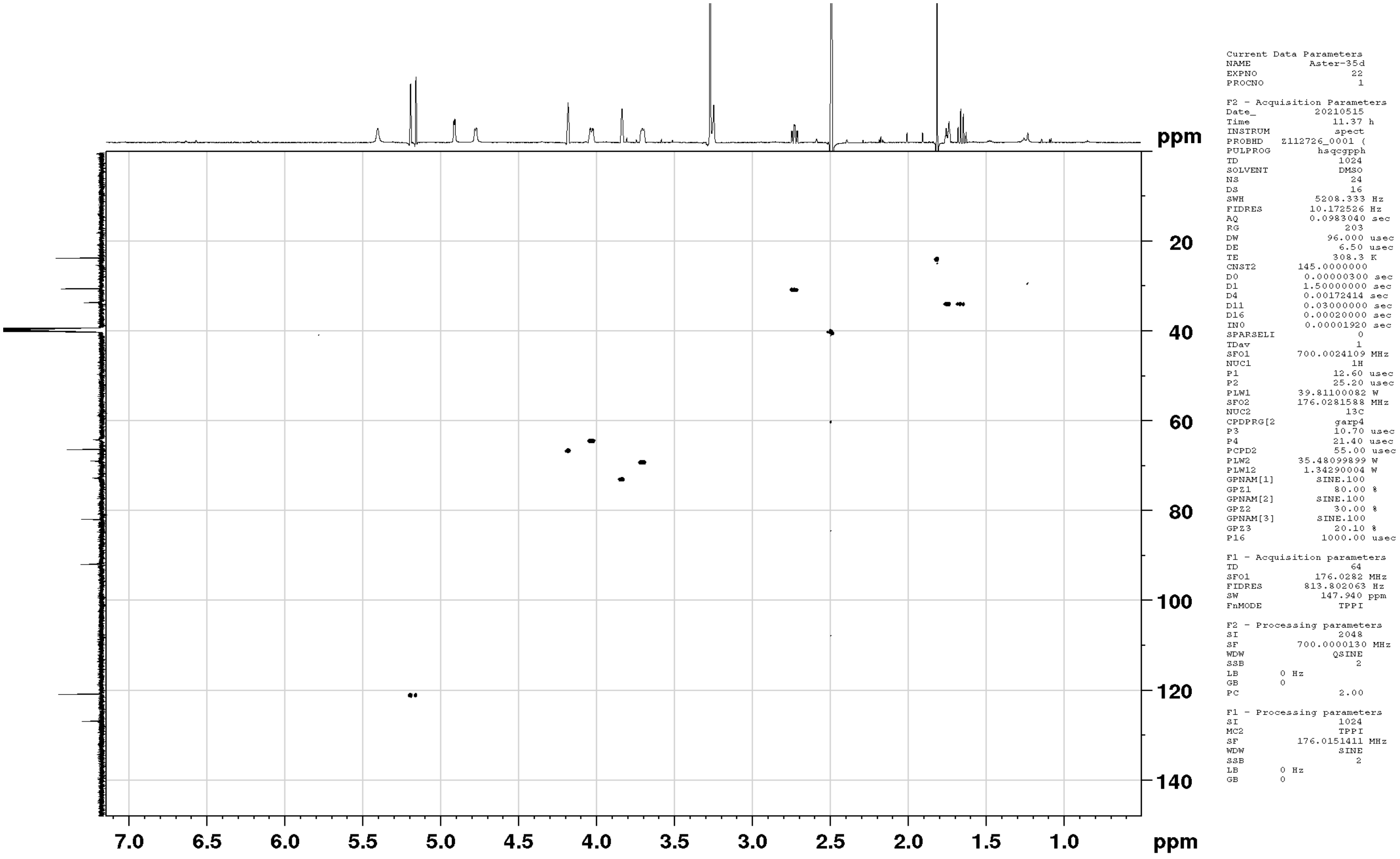

Figure S27. HSQC spectrum (700 MHz, DMSO-d6) of **2**

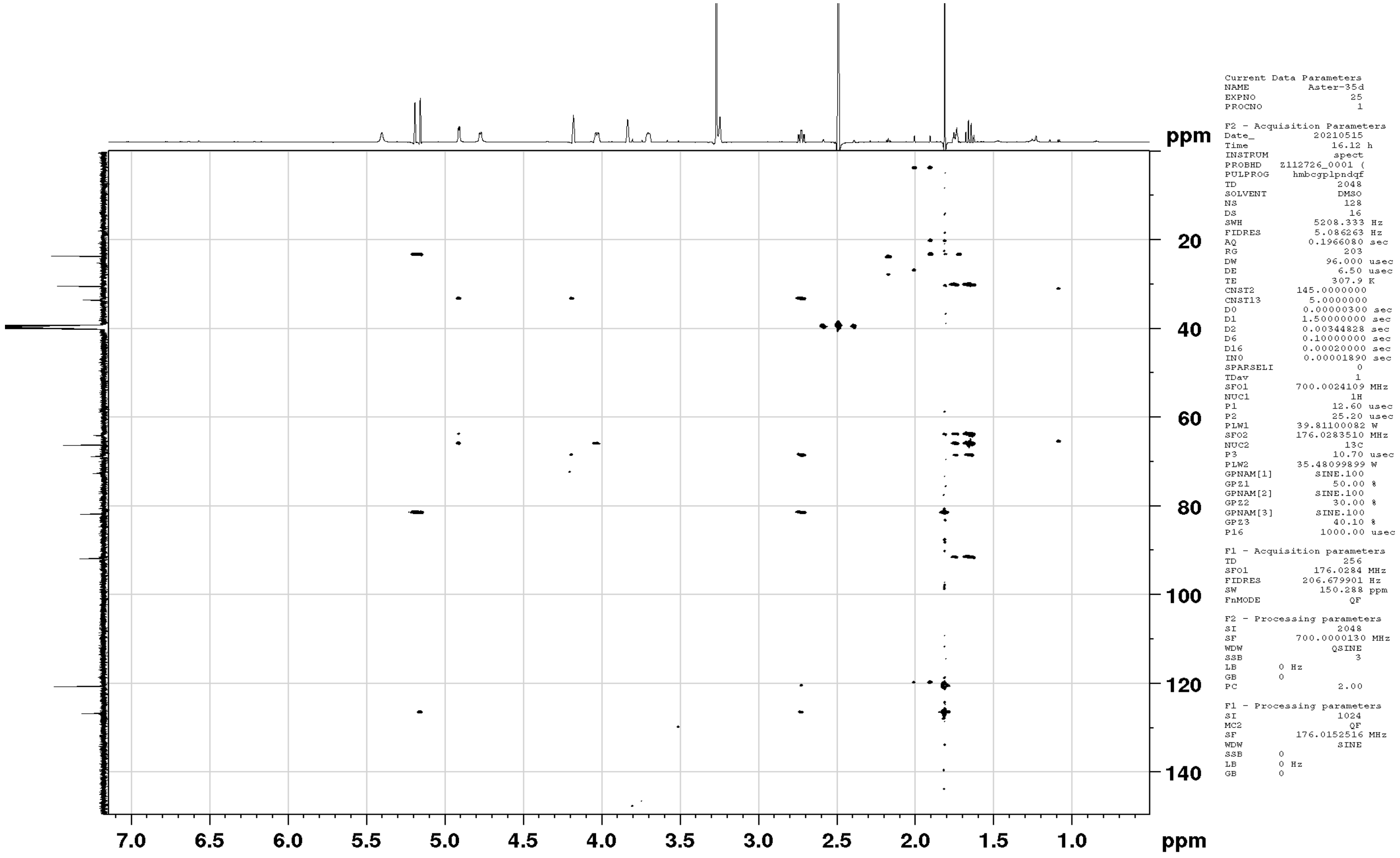

Figure S28. HMBC spectrum (700 MHz, DMSO-d6) of **2**

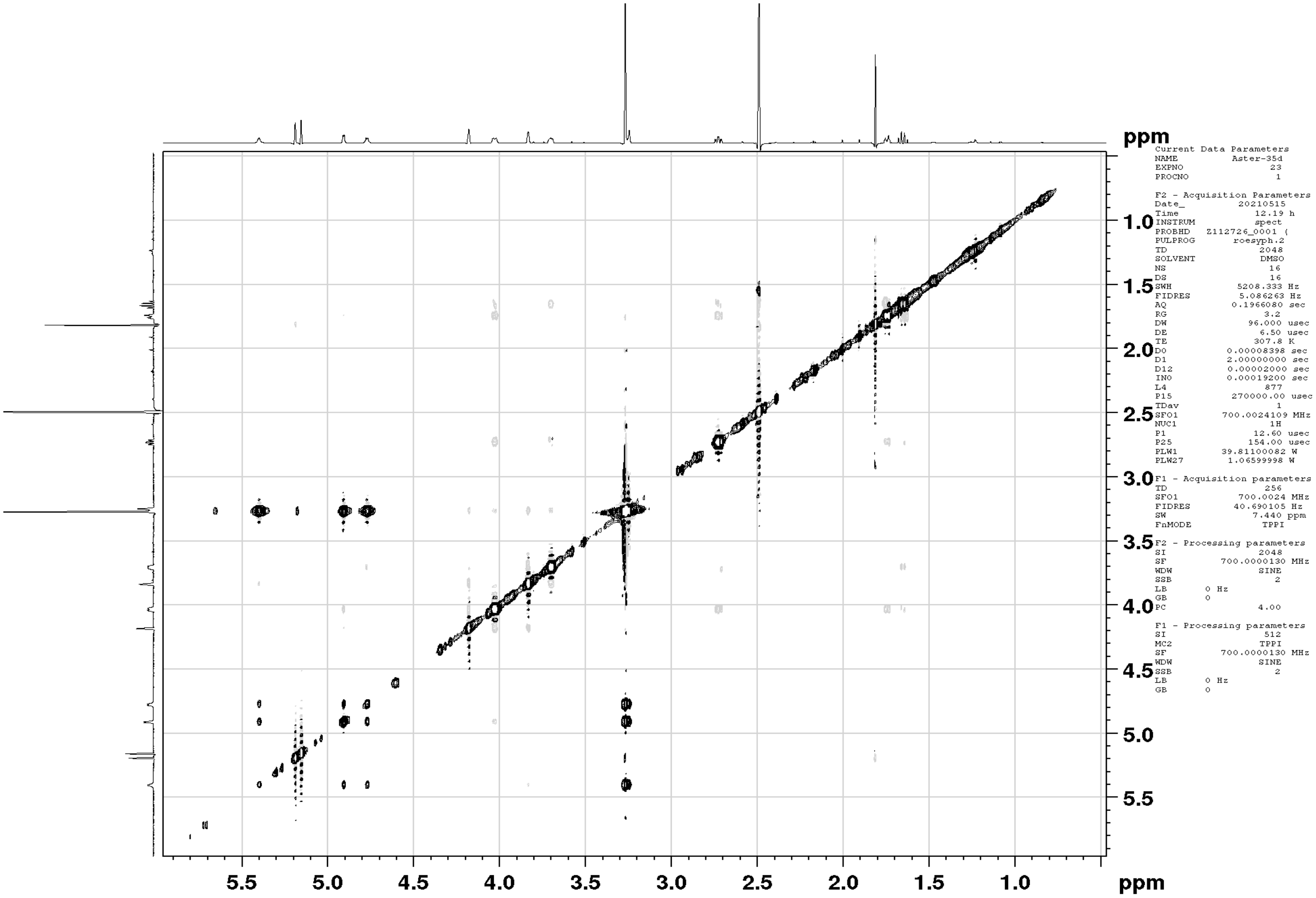

Figure S29. ROESY spectrum (700 MHz, DMSO-d6) of **2**

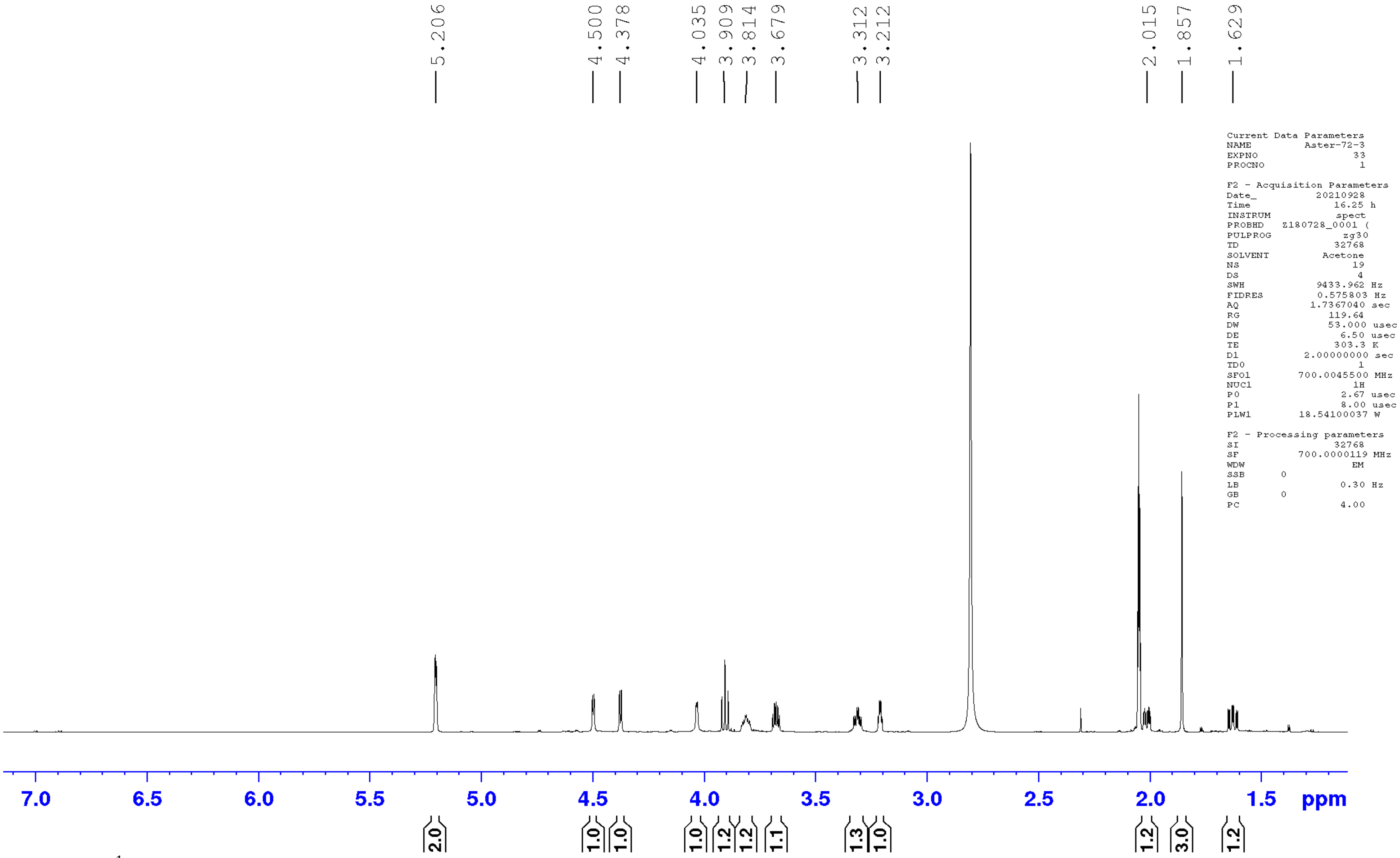

Figure S30. ^{1}H NMR spectrum (700 MHz, acetone-d_6) of **3**

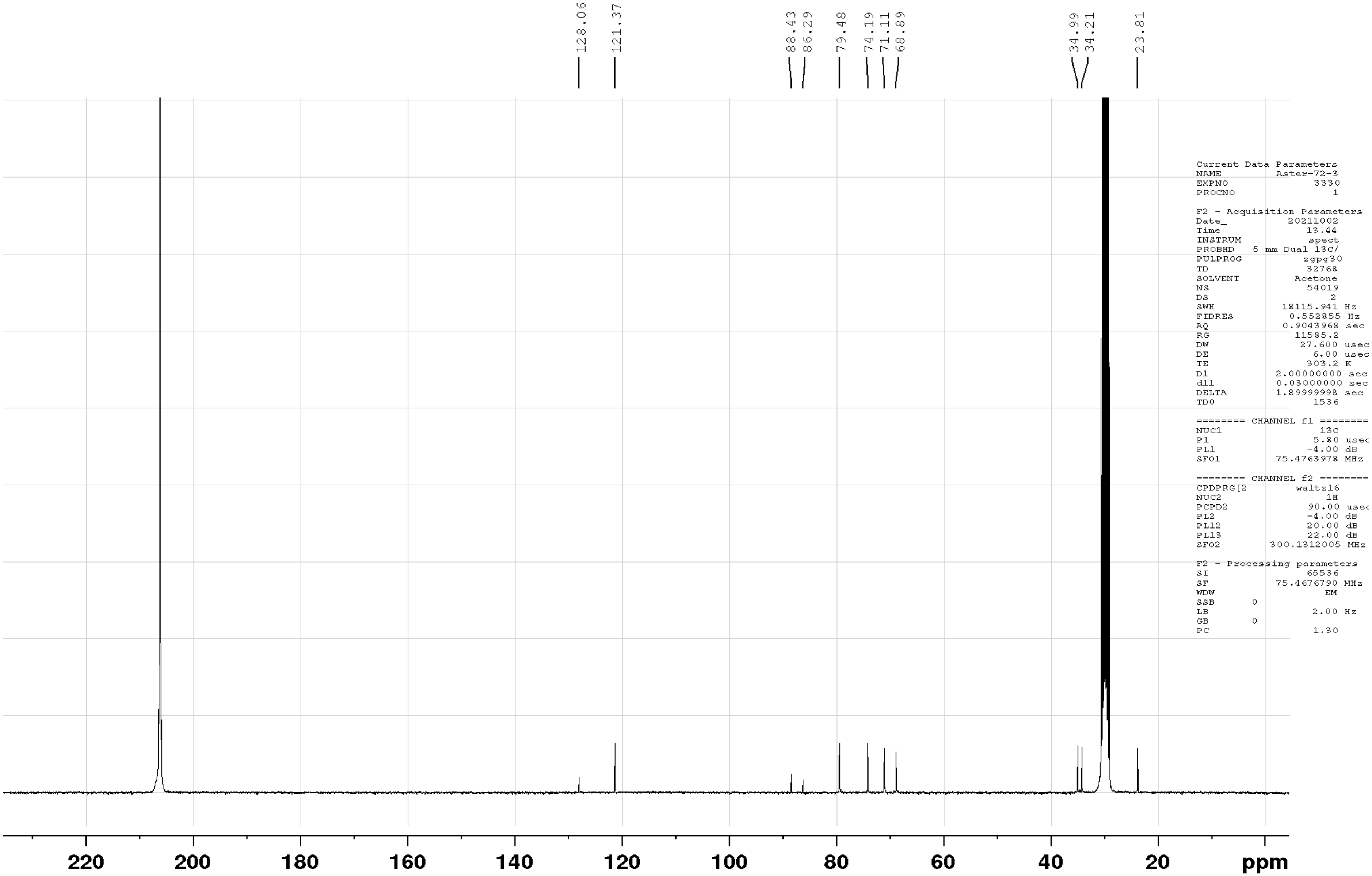

Figure S31. ^{13}C NMR spectrum (75 MHz, acetone-d_6) of **3**

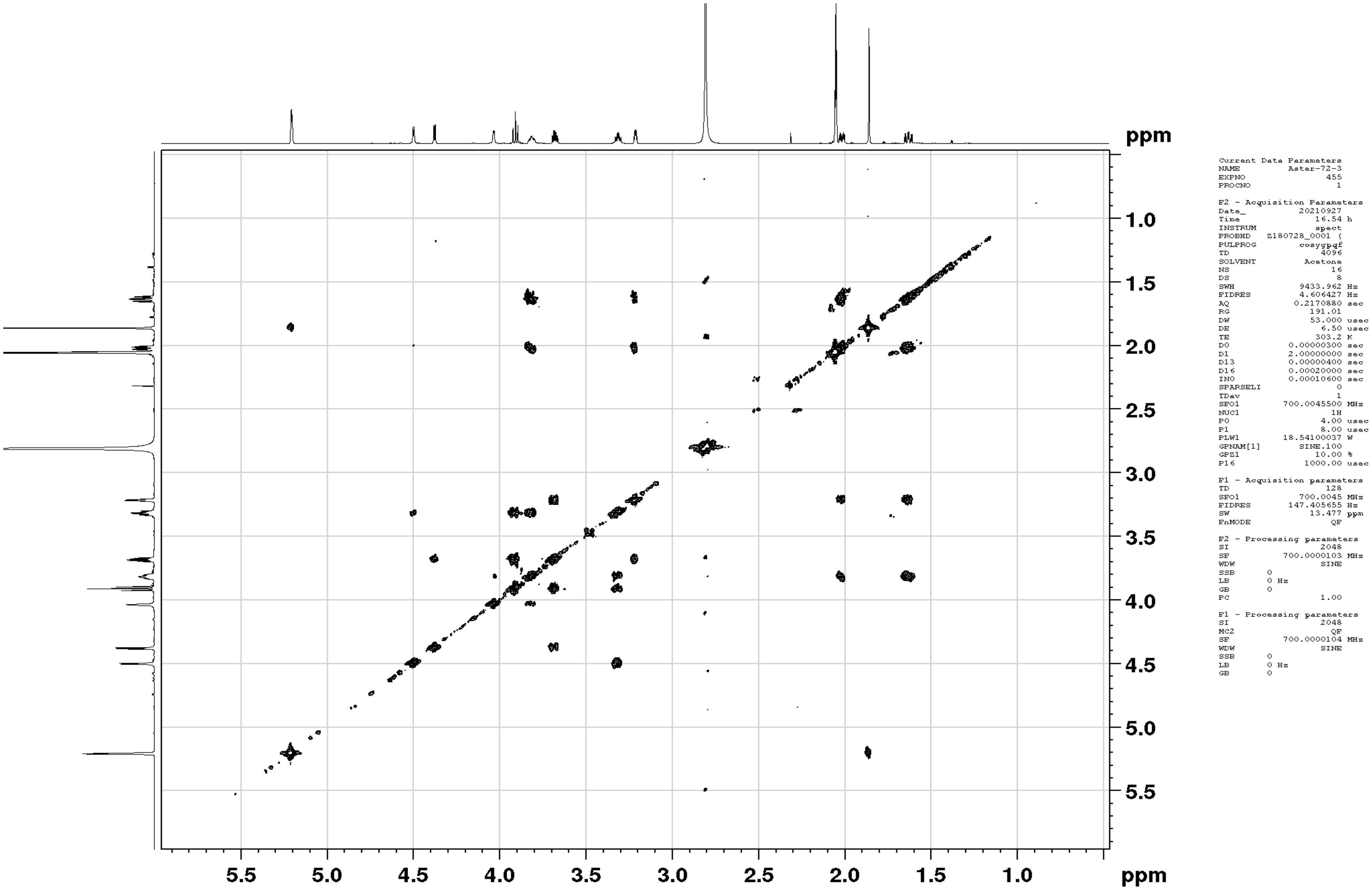

Figure S32. COSY-45 spectrum (700 MHz, acetone-d_6) of **3**

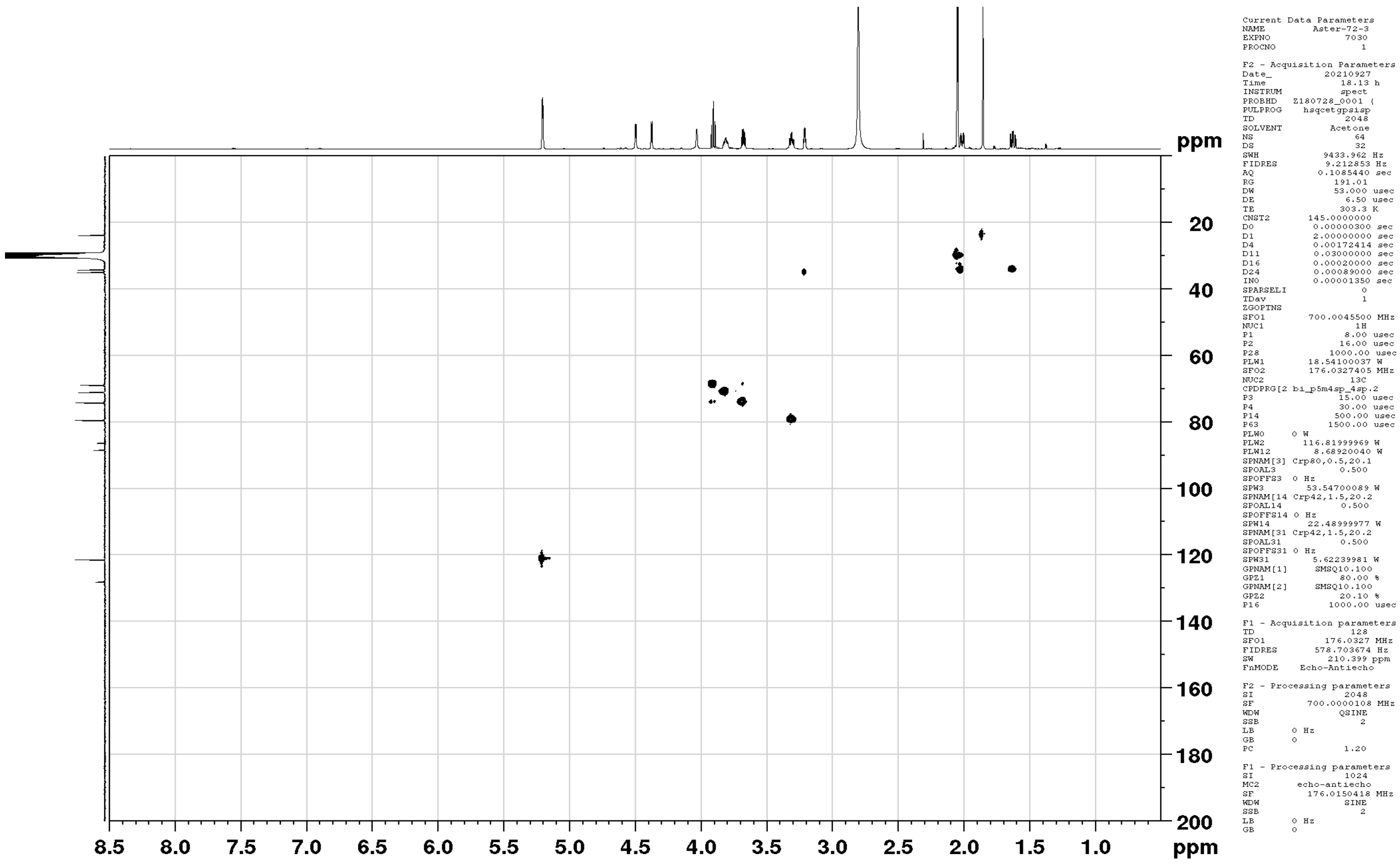

Figure S33. HSQC spectrum (700 MHz, acetone-d_6) of **3**

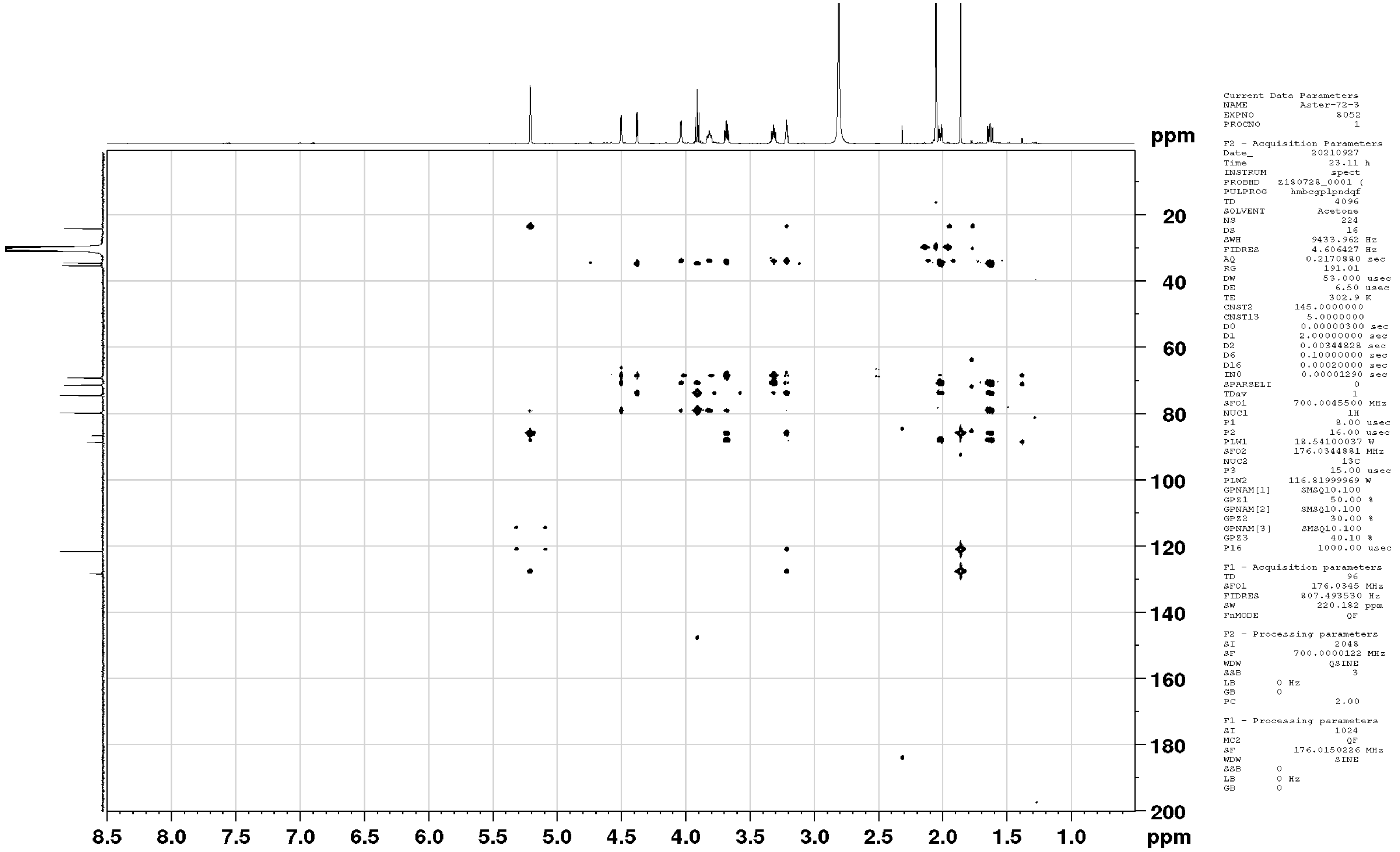

Figure S34. ^1H-^{13}C HMBC spectrum (700 MHz, acetone-d_6) of **3**

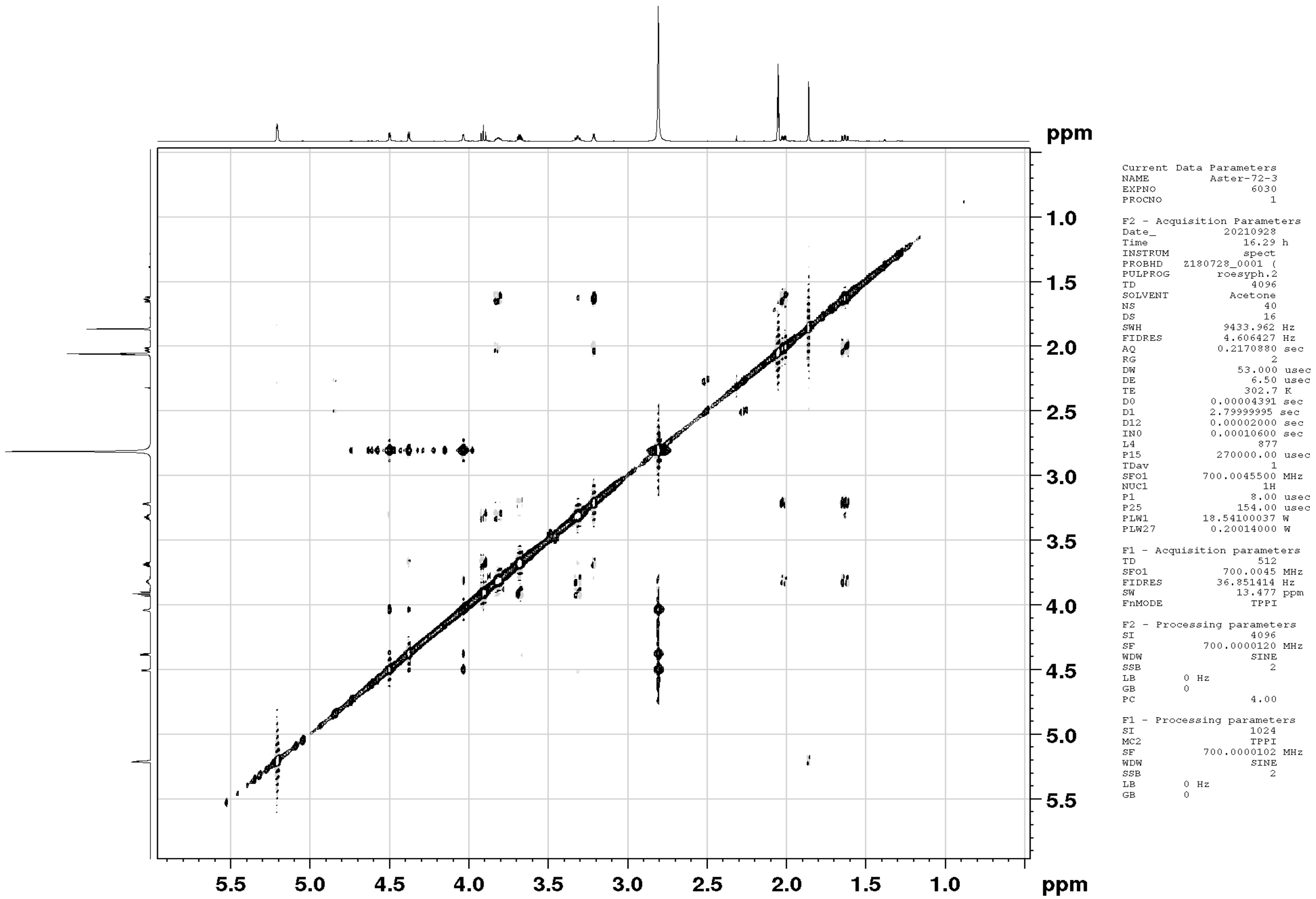

Figure S35. ROESY spectrum (700 MHz, acetone-d_6) of **3**

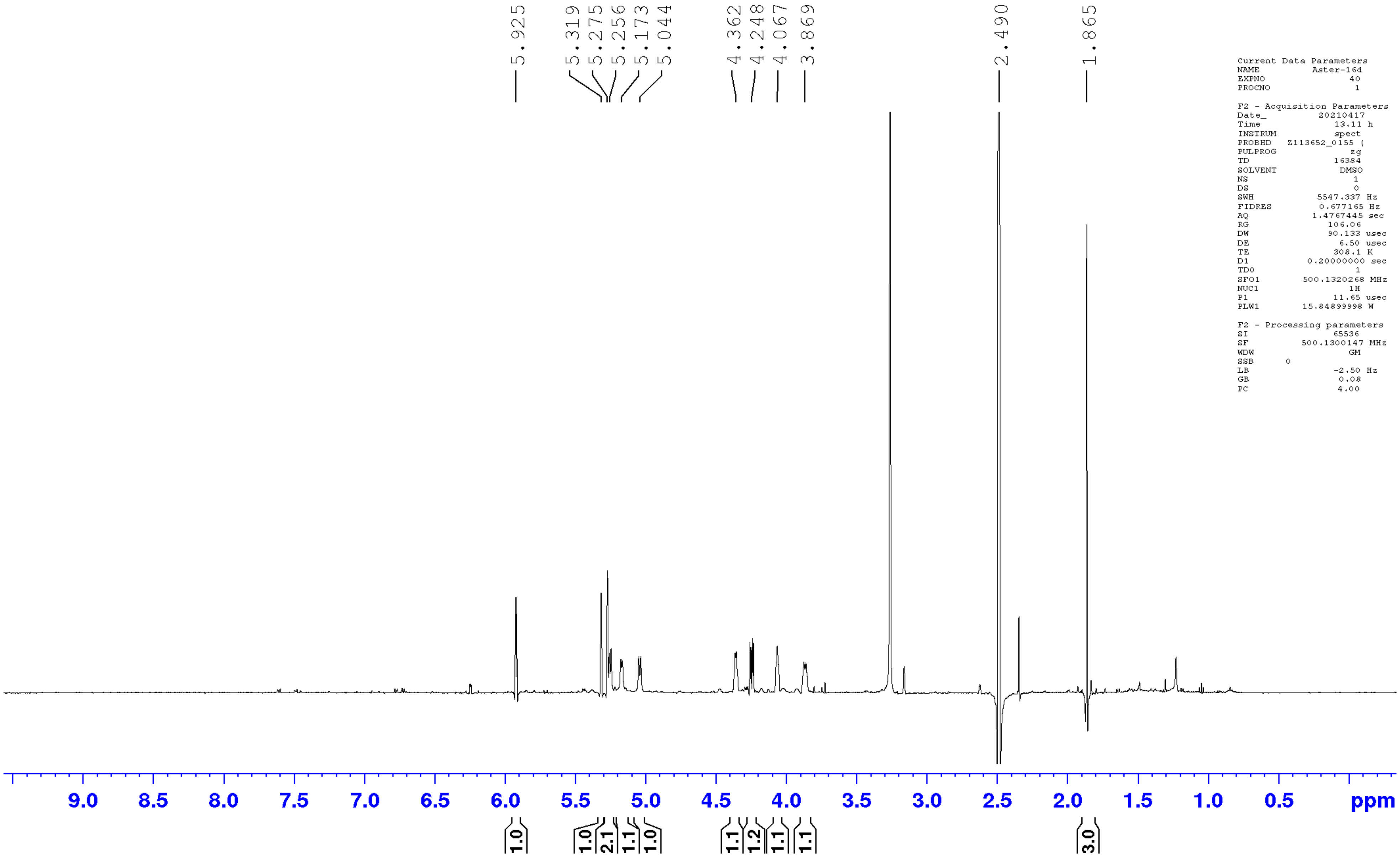

Figure S36. ^{1}H NMR spectrum (500 MHz, DMSO-d_6) of **4**

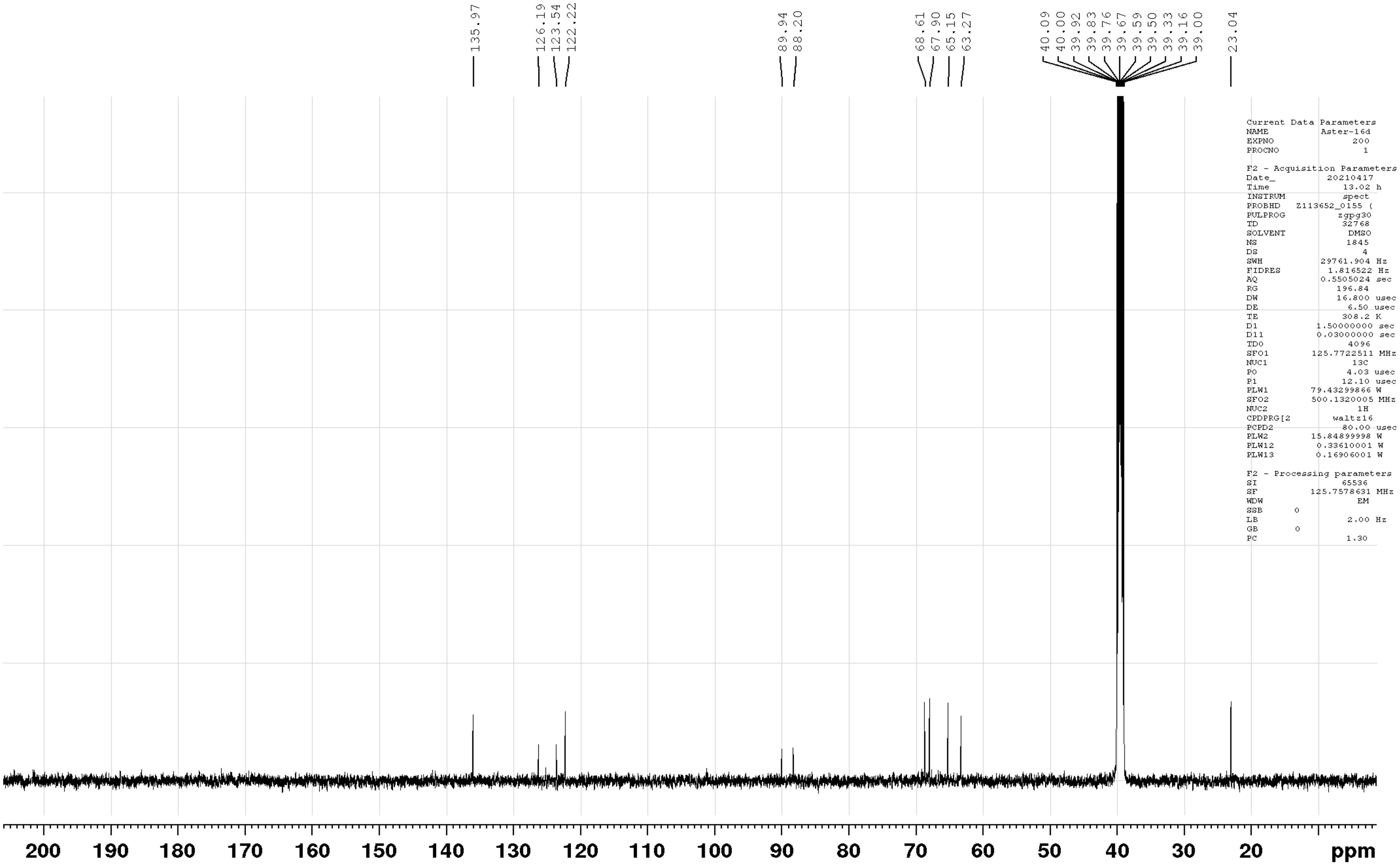

Figure S37. ^{13}C NMR spectrum (125 MHz, DMSO-d_6) of **4**

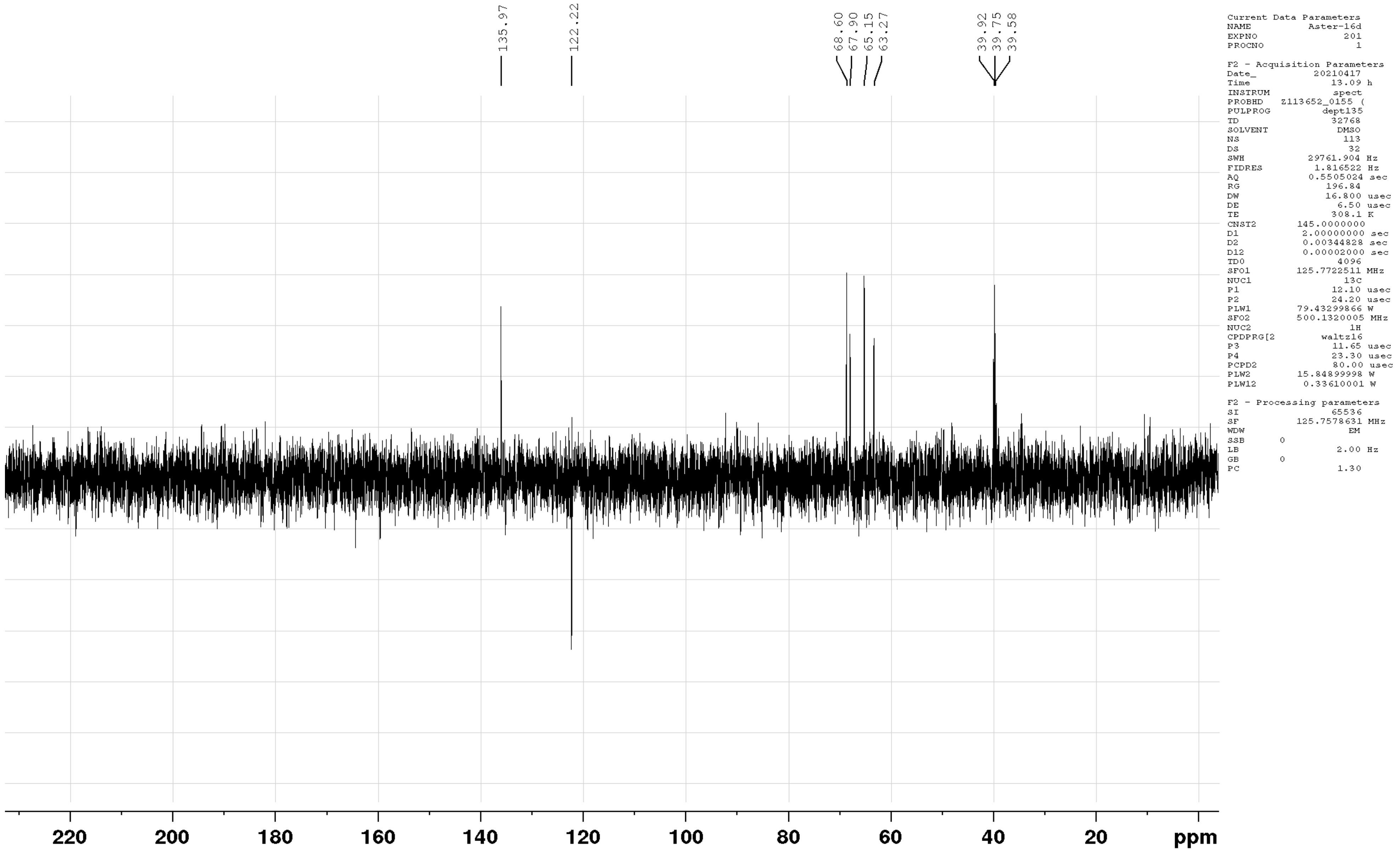

Figure S38. DEPT-135 NMR spectrum (125 MHz, DMSO-d_6) of **4**

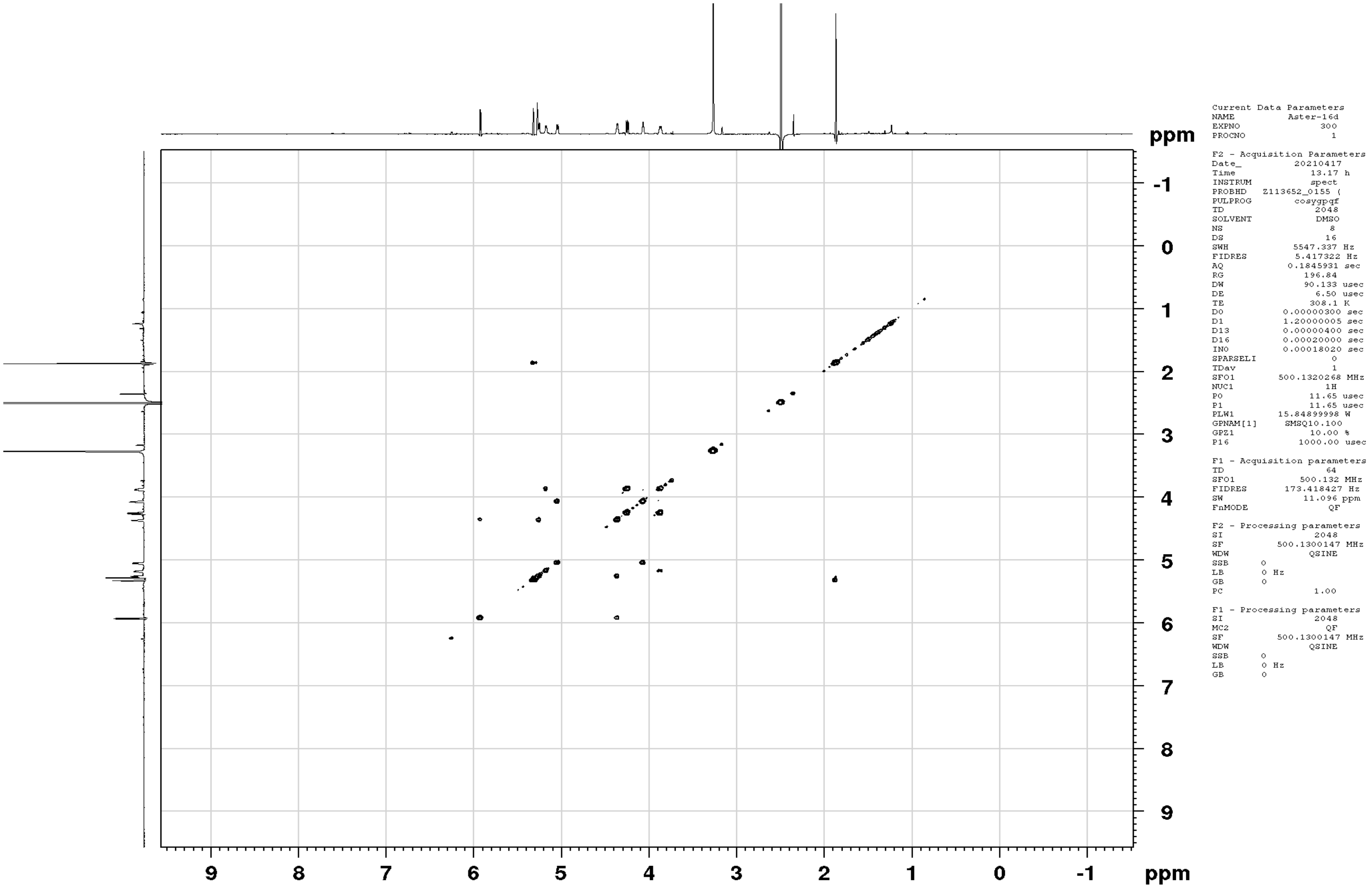

Figure S39. COSY-45 spectrum (500 MHz, DMSO-d_6) of **4**

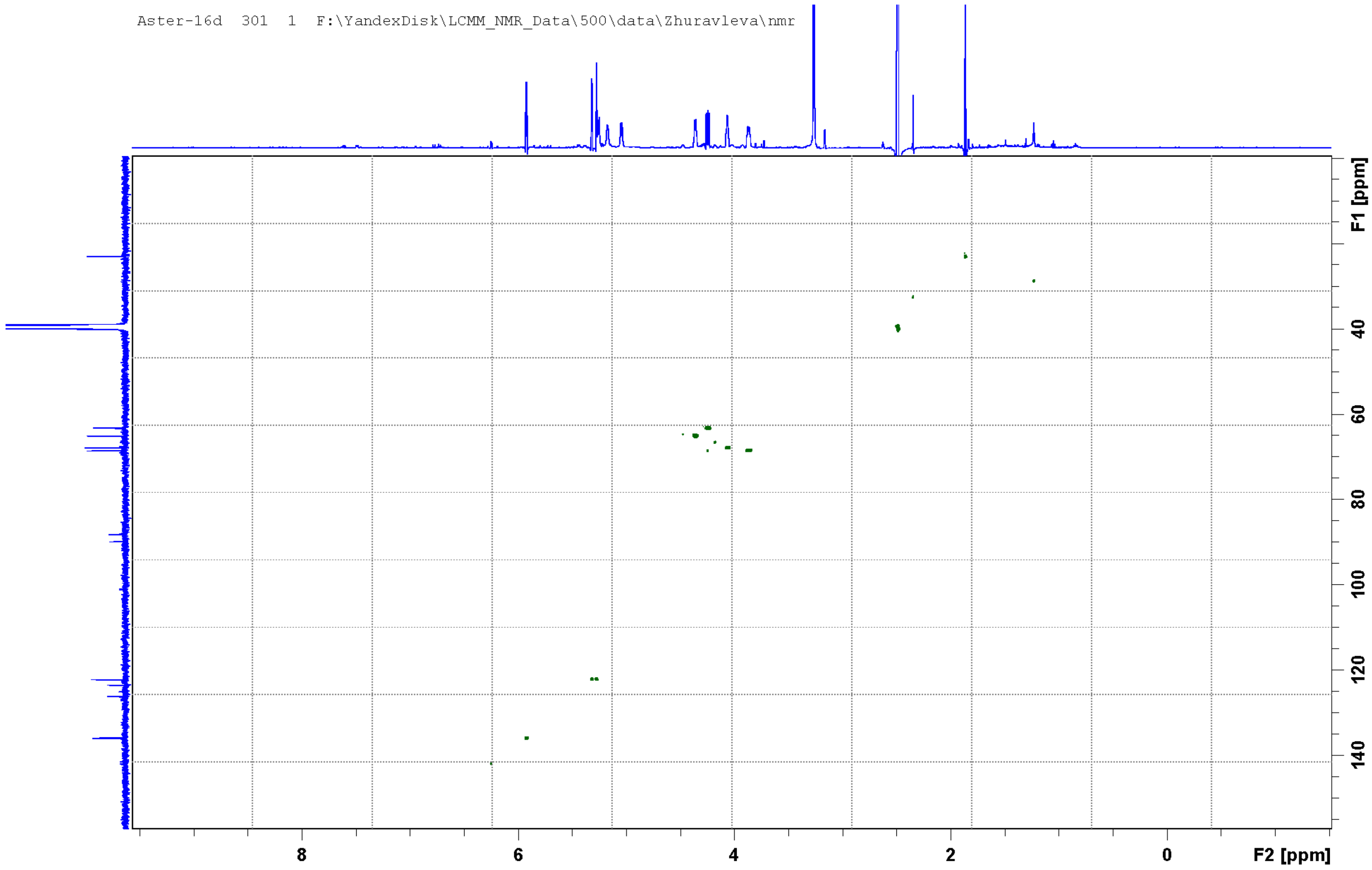

Figure S40. HSQC spectrum (500 MHz, DMSO-d_6) of **4**

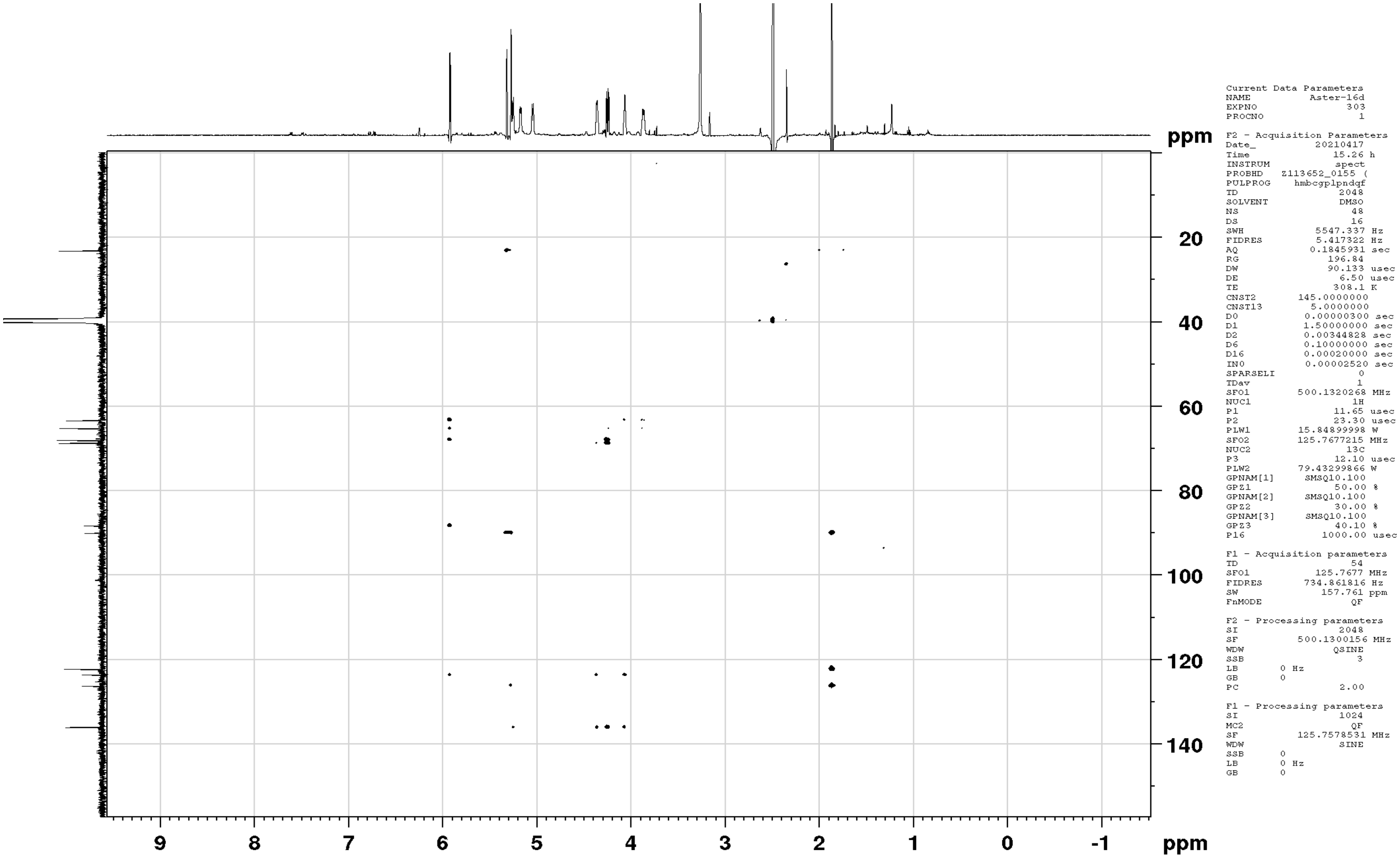

Figure S41. HMBC spectrum (500 MHz, DMSO-d_6) of **4**

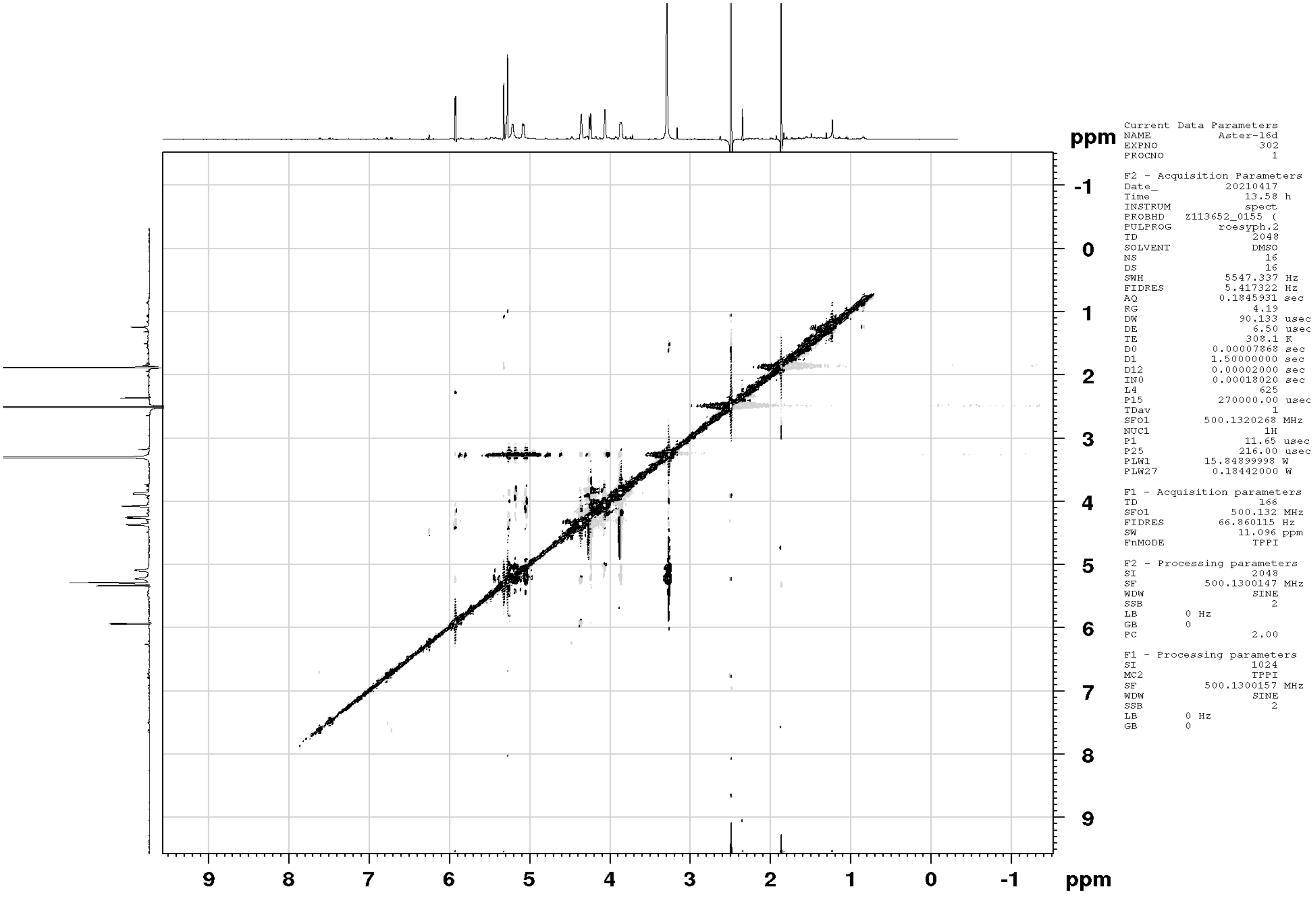

Figure S42. ROESY spectrum (500 MHz, DMSO-d_6) of **4**

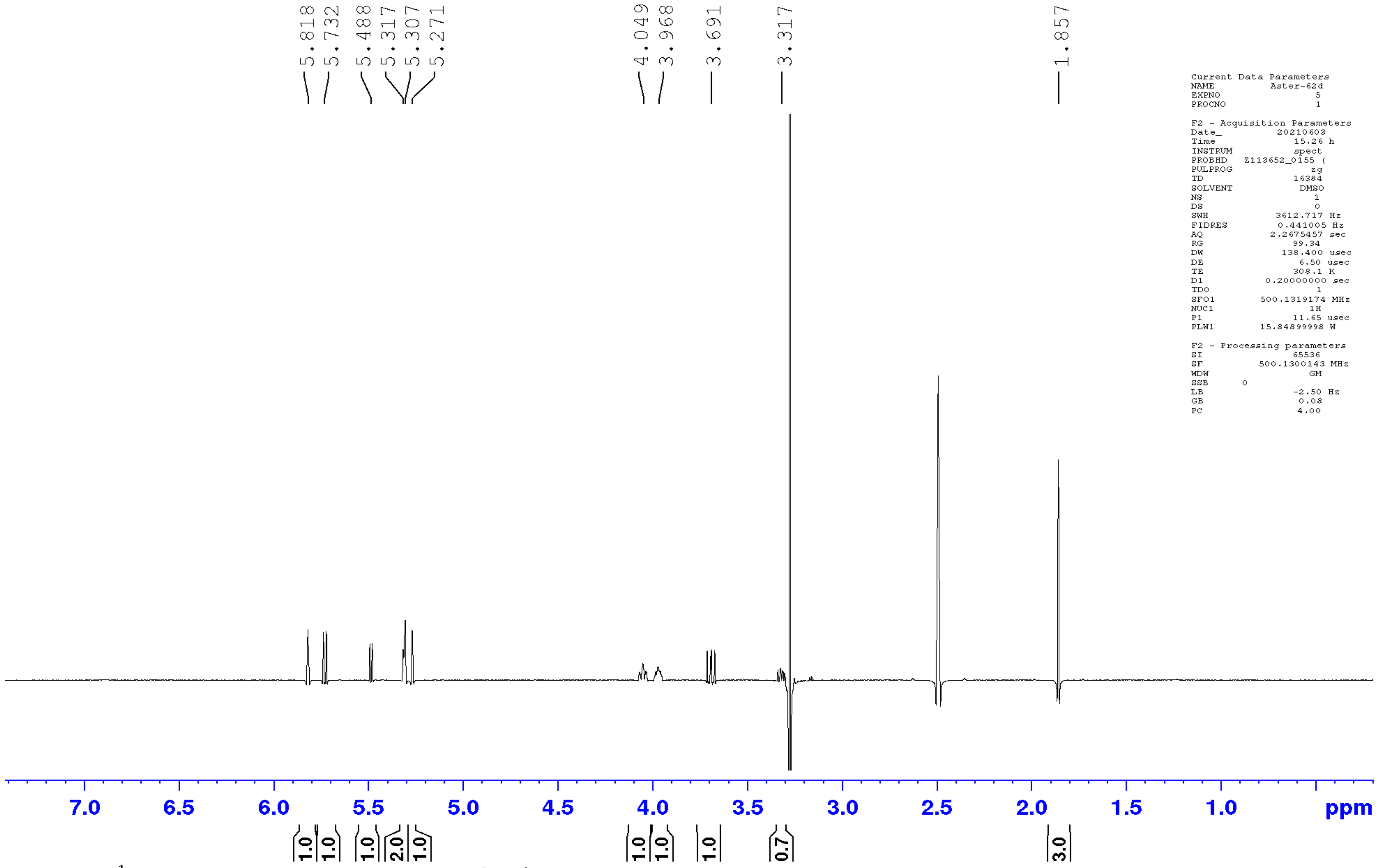

Figure S43. ^{1}H NMR spectrum (500 MHz, DMSO-d$_6$) of **5**

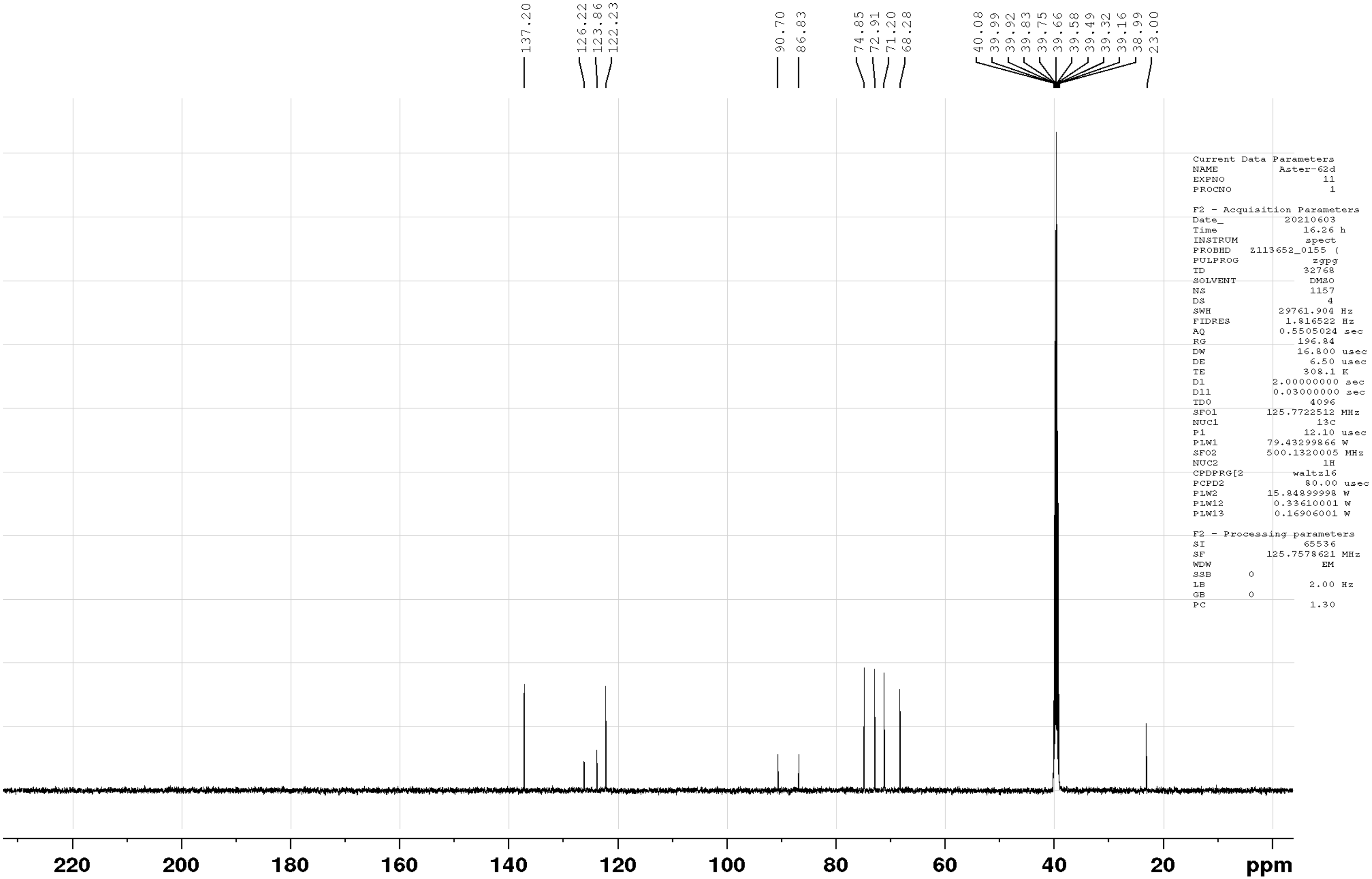

Figure S44. ^{13}C NMR spectrum (125 MHz, DMSO-d_6) of **5**

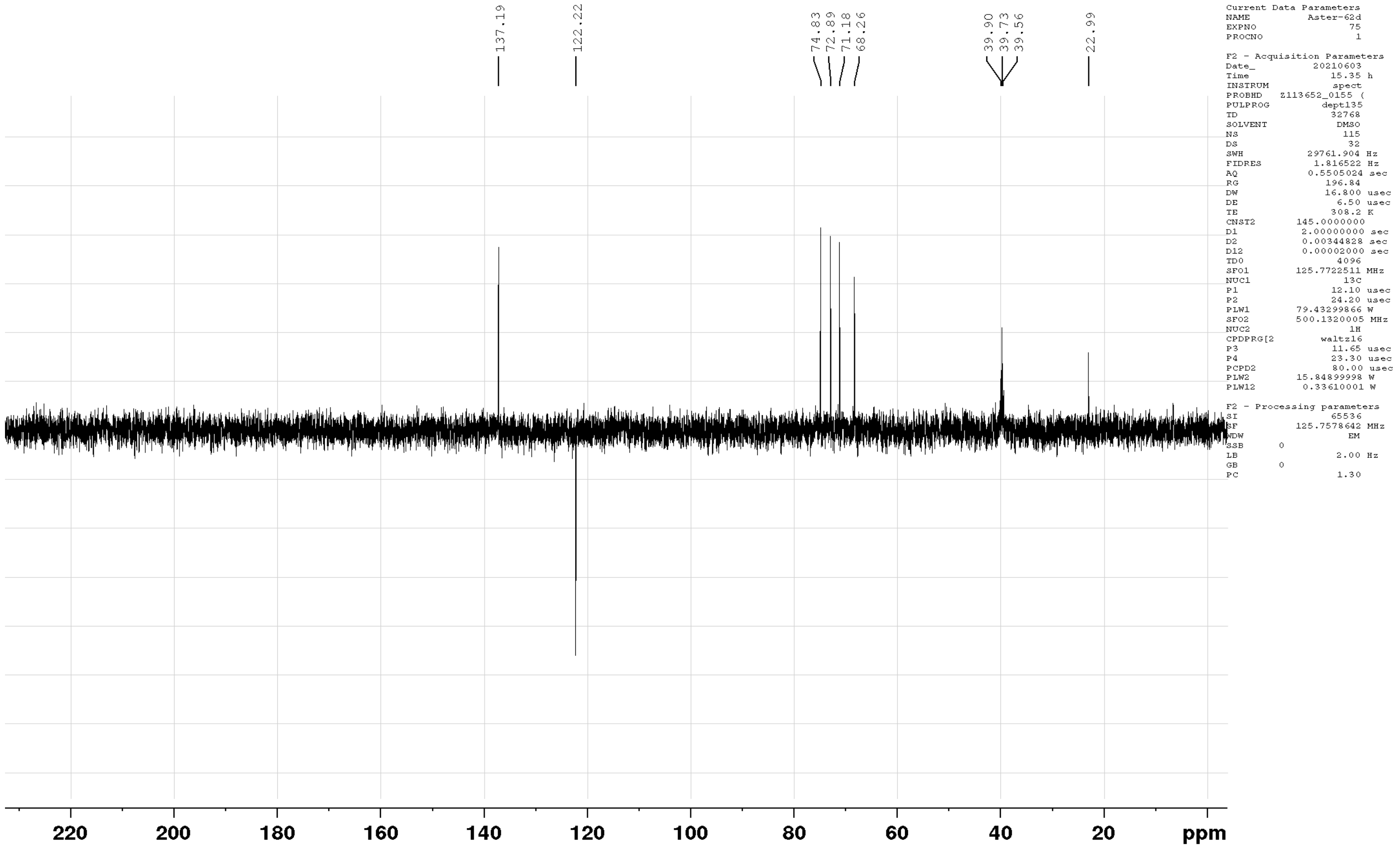

Figure S45. DEPT-135 NMR spectrum (125 MHz, DMSO-d_6) of **5**

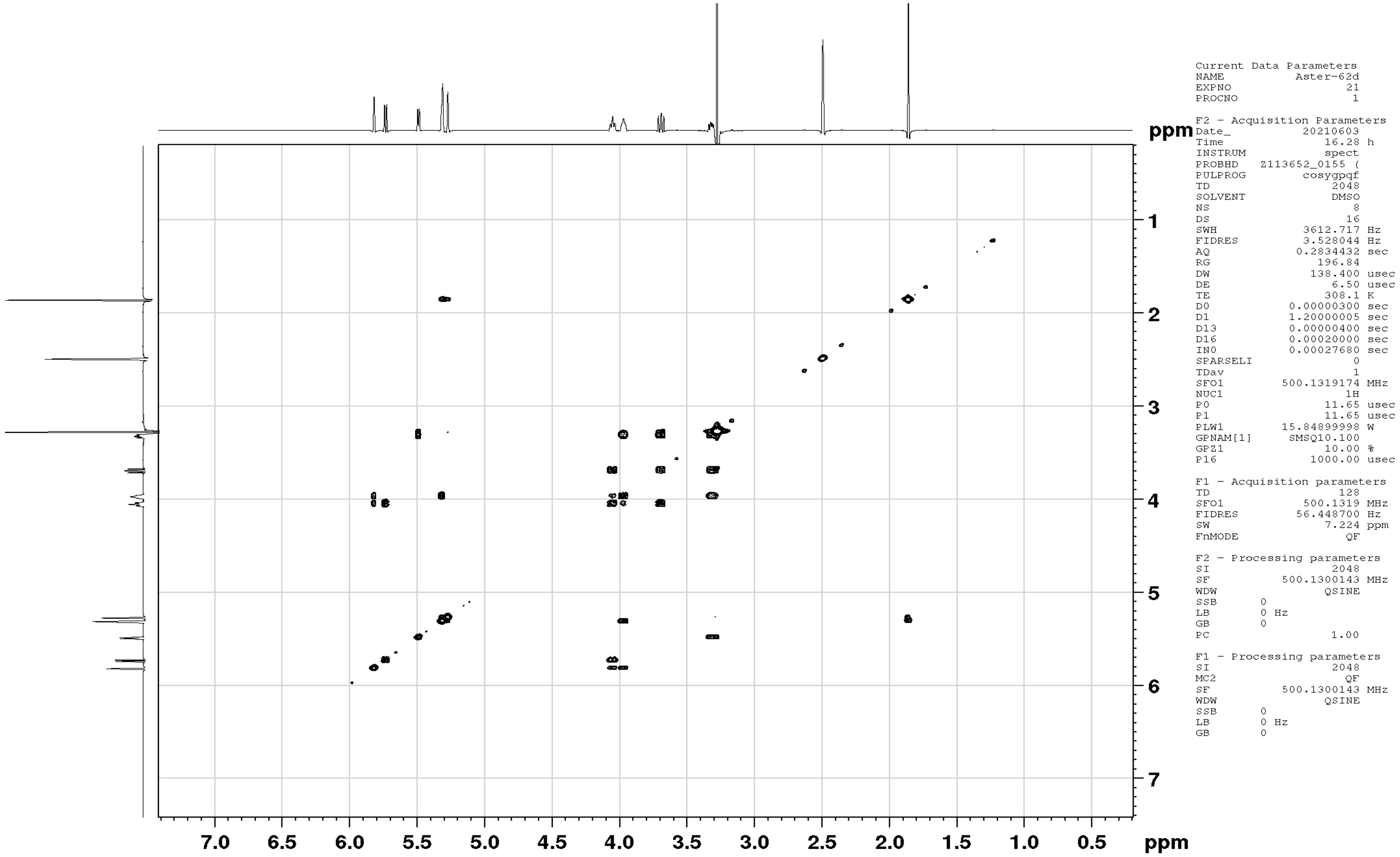

Figure S46. COSY-45 spectrum (500 MHz, DMSO-d_6) of **5**

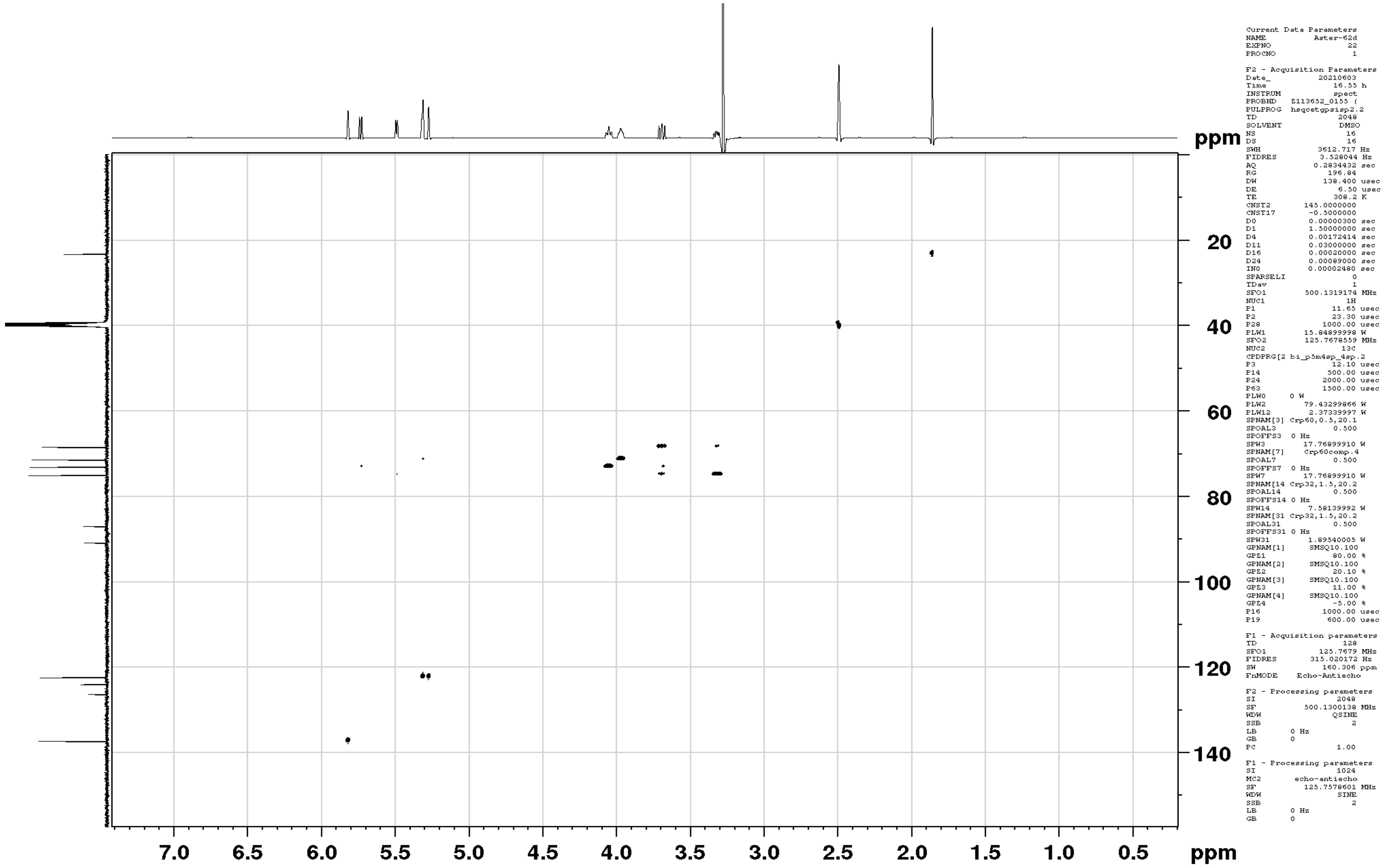

Figure S47. HSQC spectrum (500 MHz, DMSO-d_6) of **5**

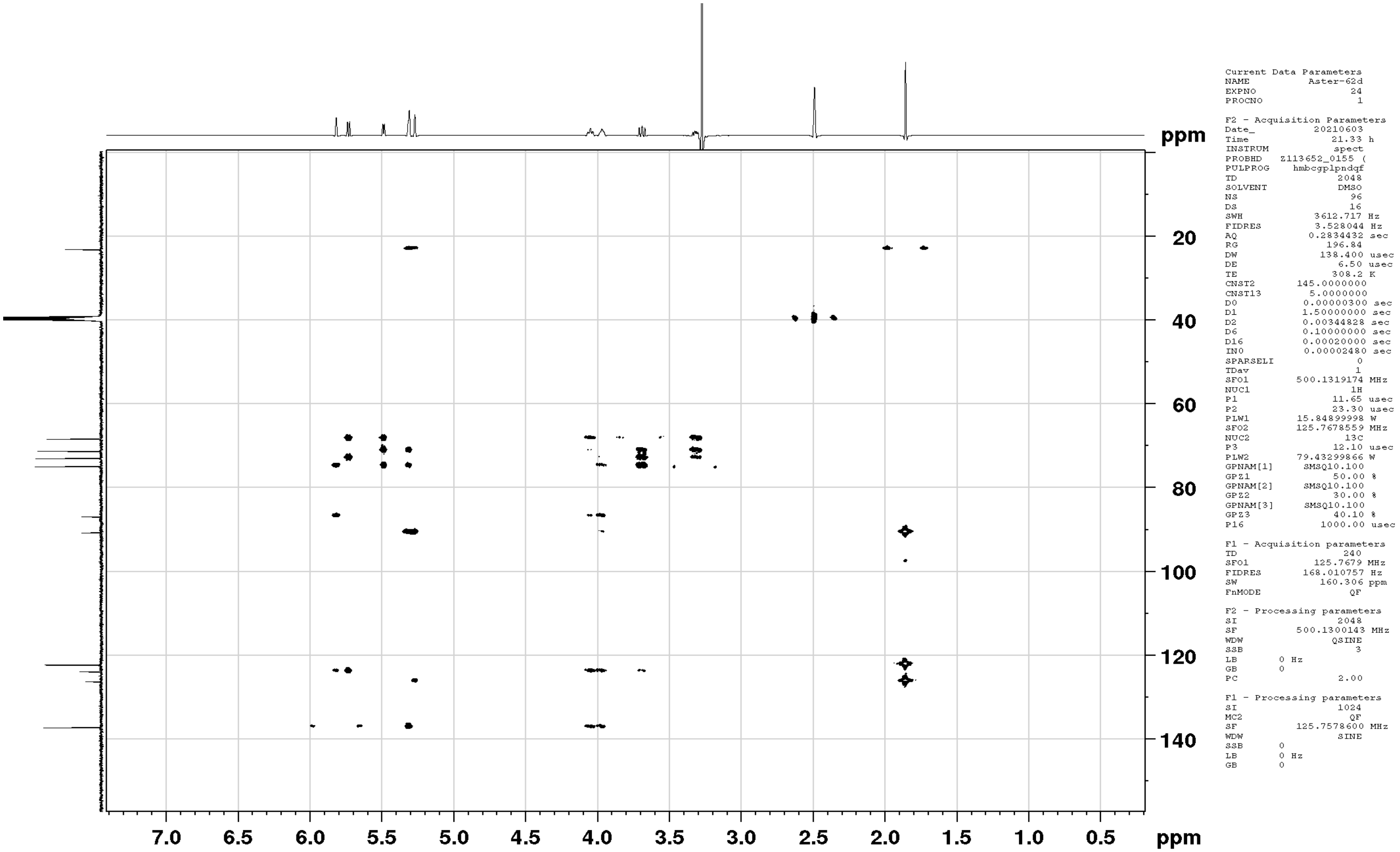

Figure S48. HMBC spectrum (500 MHz, DMSO-d_6) of **5**

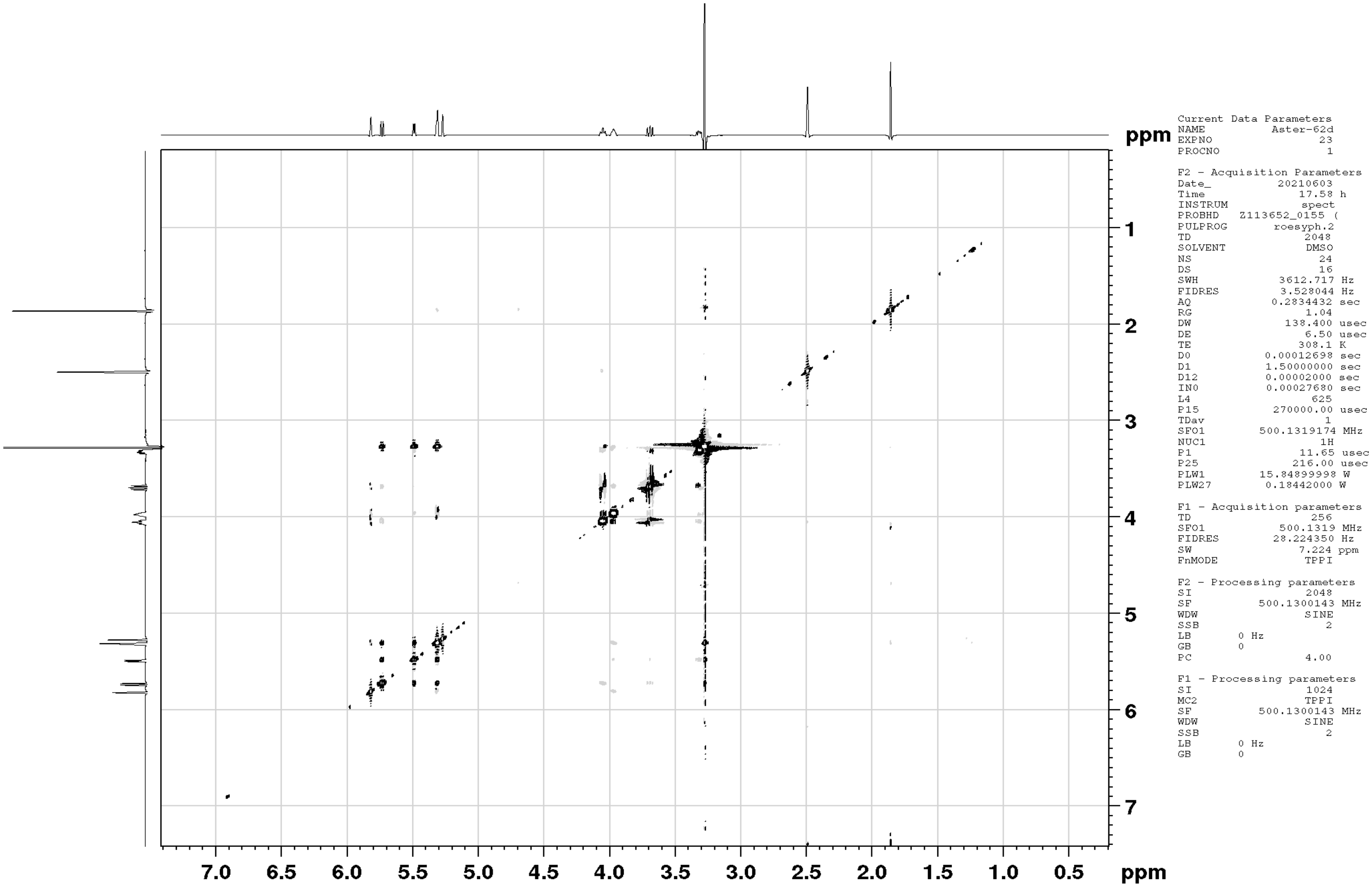

Figure S49. ROESY spectrum (500 MHz, DMSO-d_6) of **5**

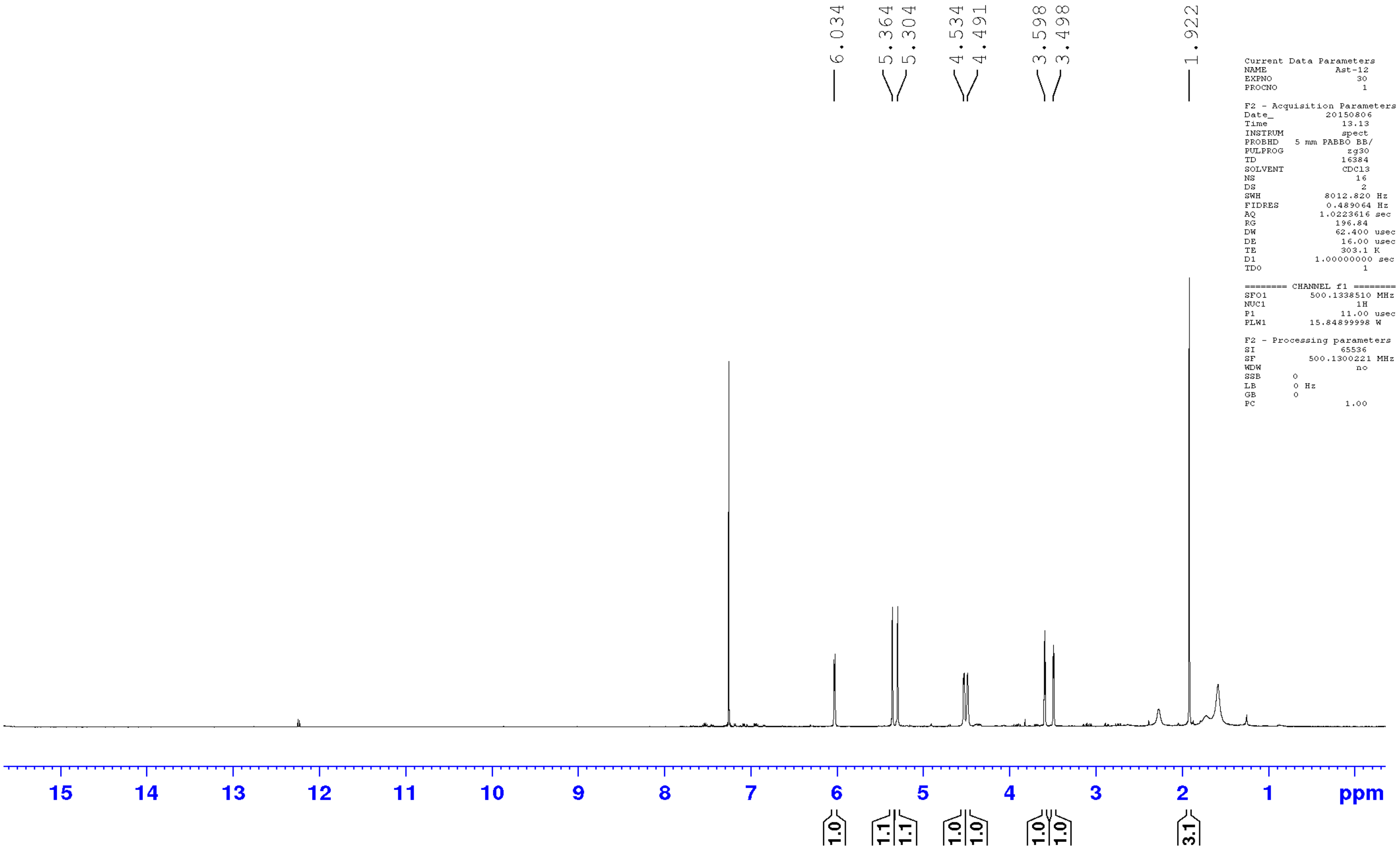

Figure S50. ^{1}H NMR spectrum (500 MHz, $CDCl_3$) of **6**

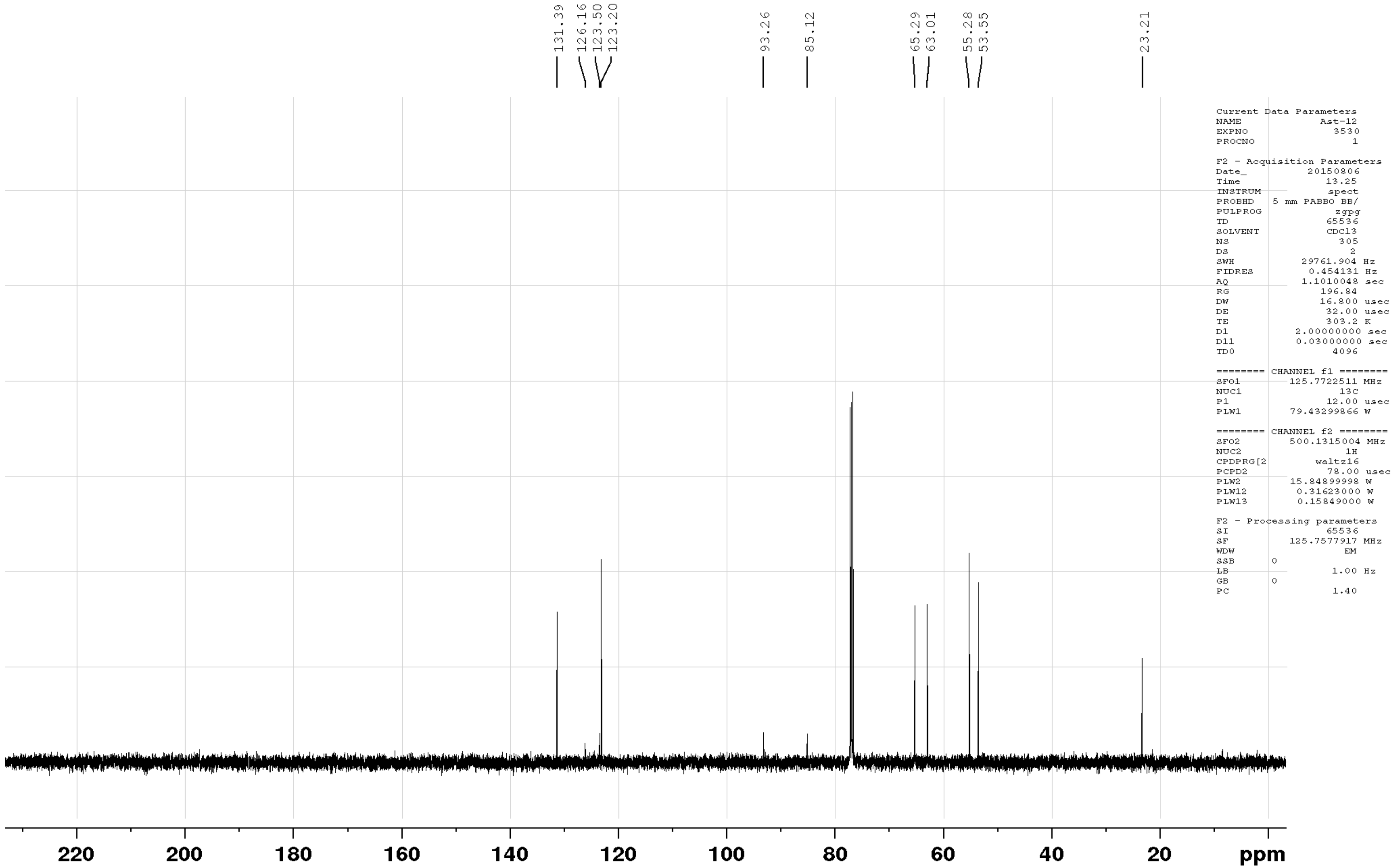

Figure S51. ^{13}C NMR spectrum (125 MHz, $CDCl_3$) of **6**

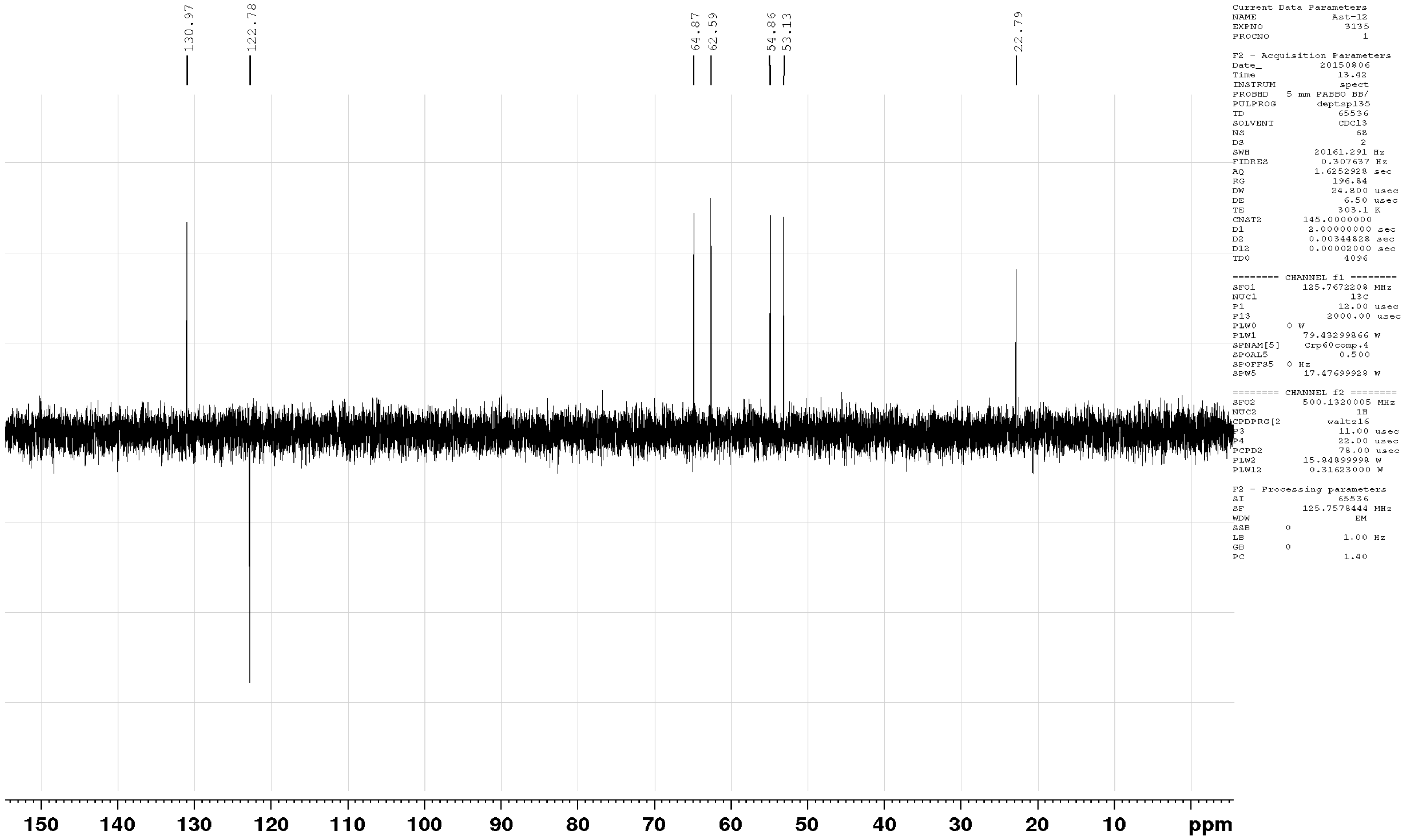

Figure S52. DEPT-135 NMR spectrum (125MHz, $CDCl_3$) of **6**

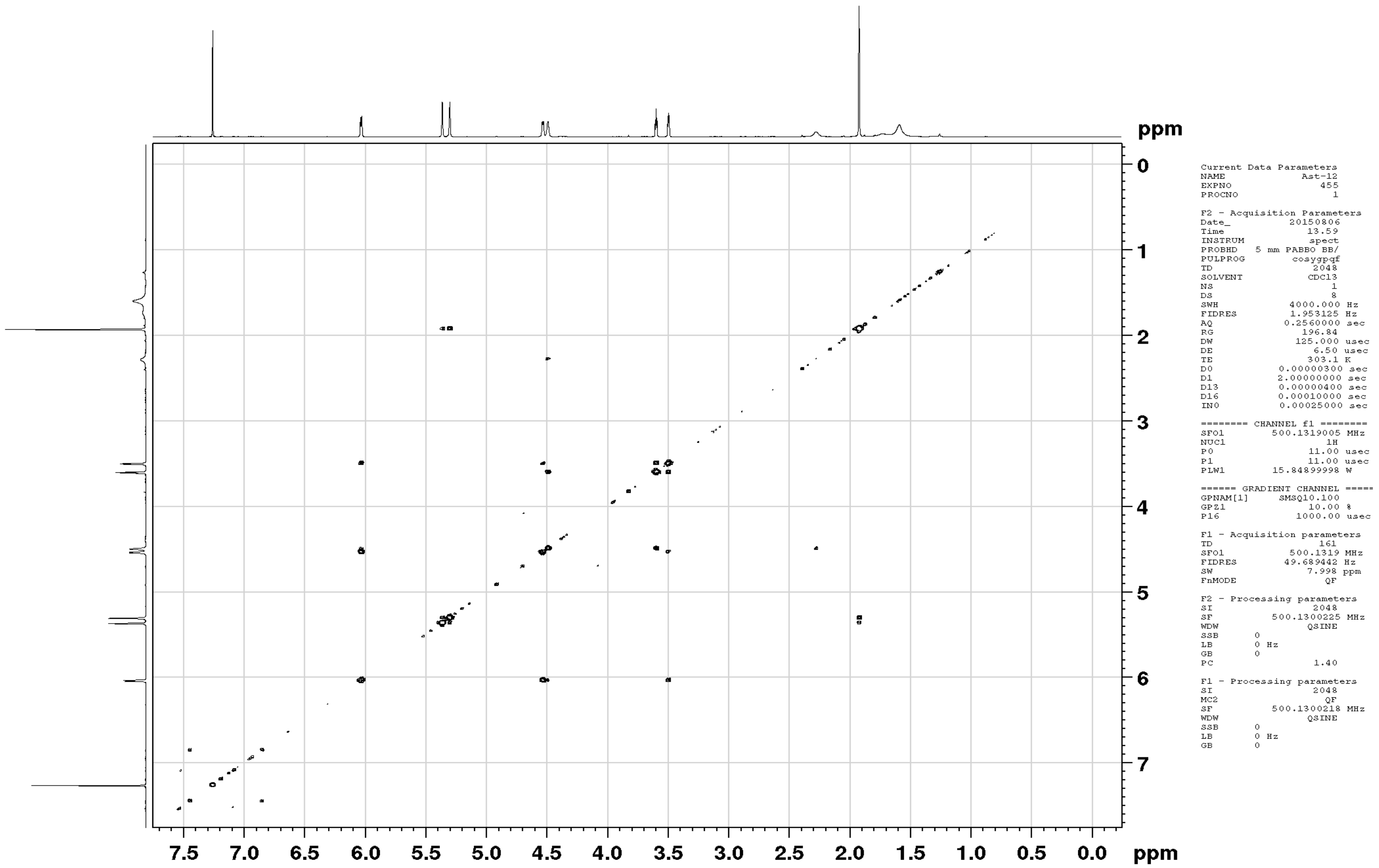

Figure S53. COSY-45 spectrum (500 MHz, $CDCl_3$) of **6**

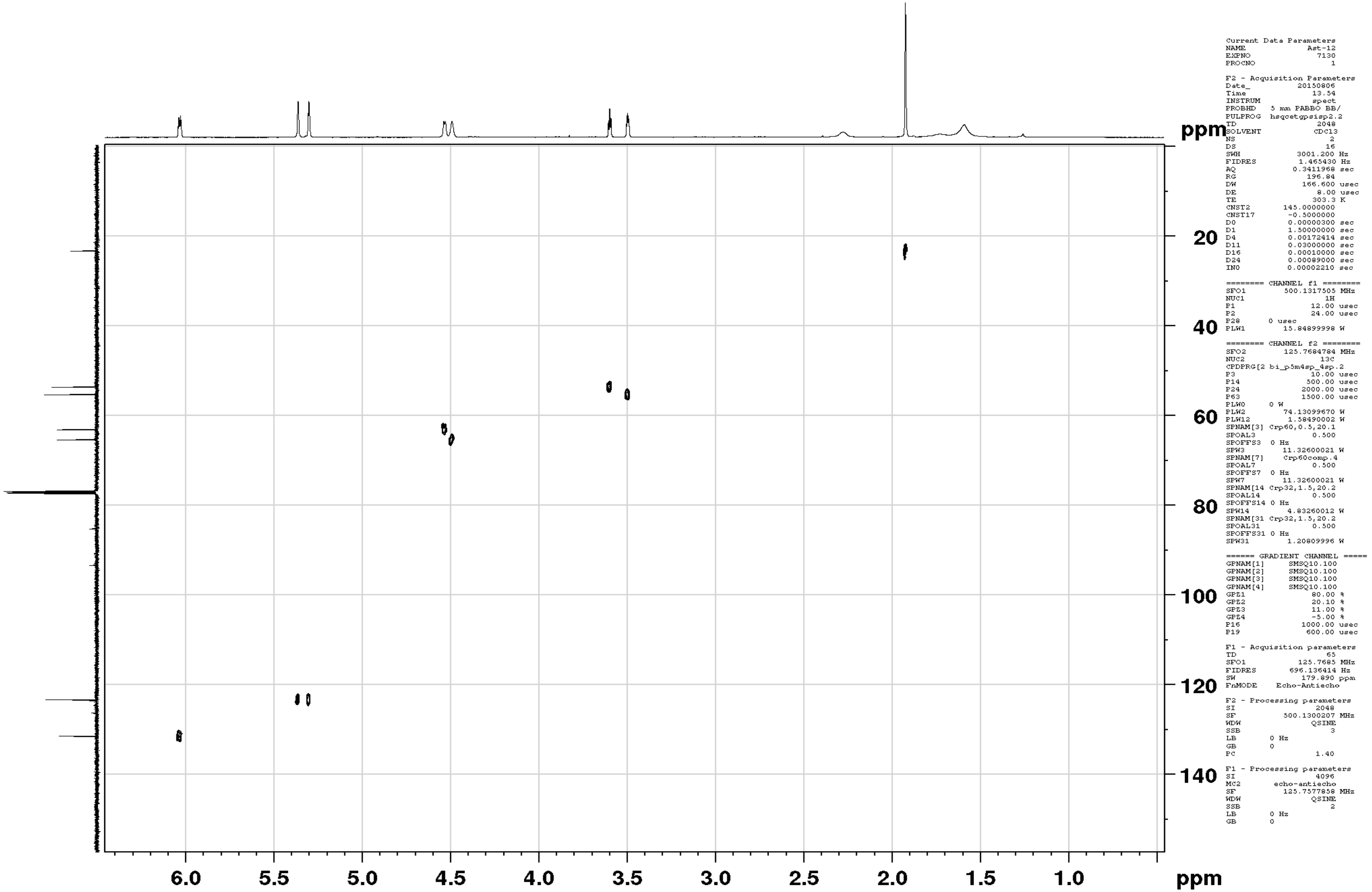

Figure S54. HSQC spectrum (500 MHz, $CDCl_3$) of **6**

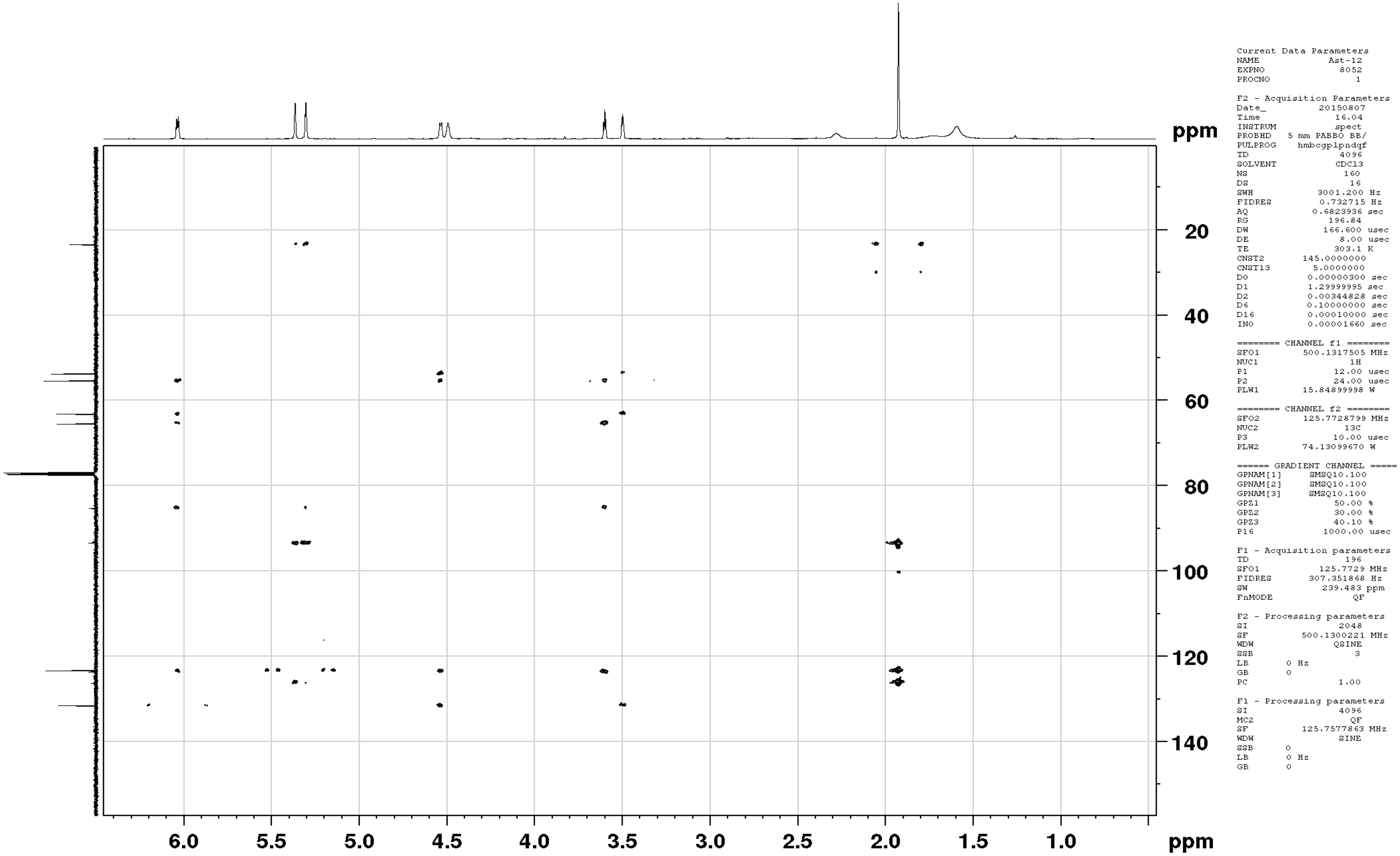

Figure S55. HMBC spectrum (500 MHz, $CDCl_3$) of **6**

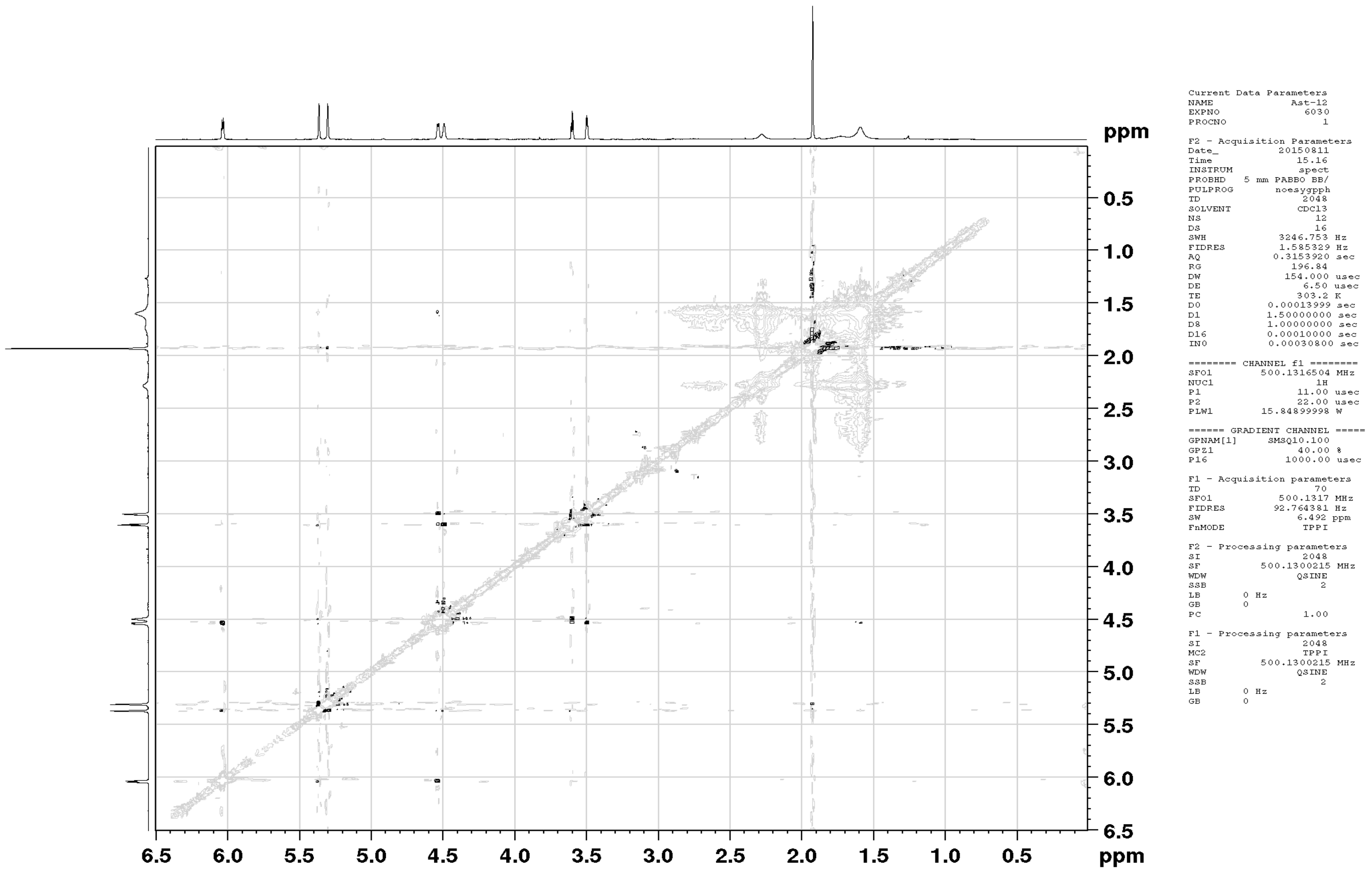

Figure S56. NOESY spectrum (500 MHz, $CDCl_3$) of **6**

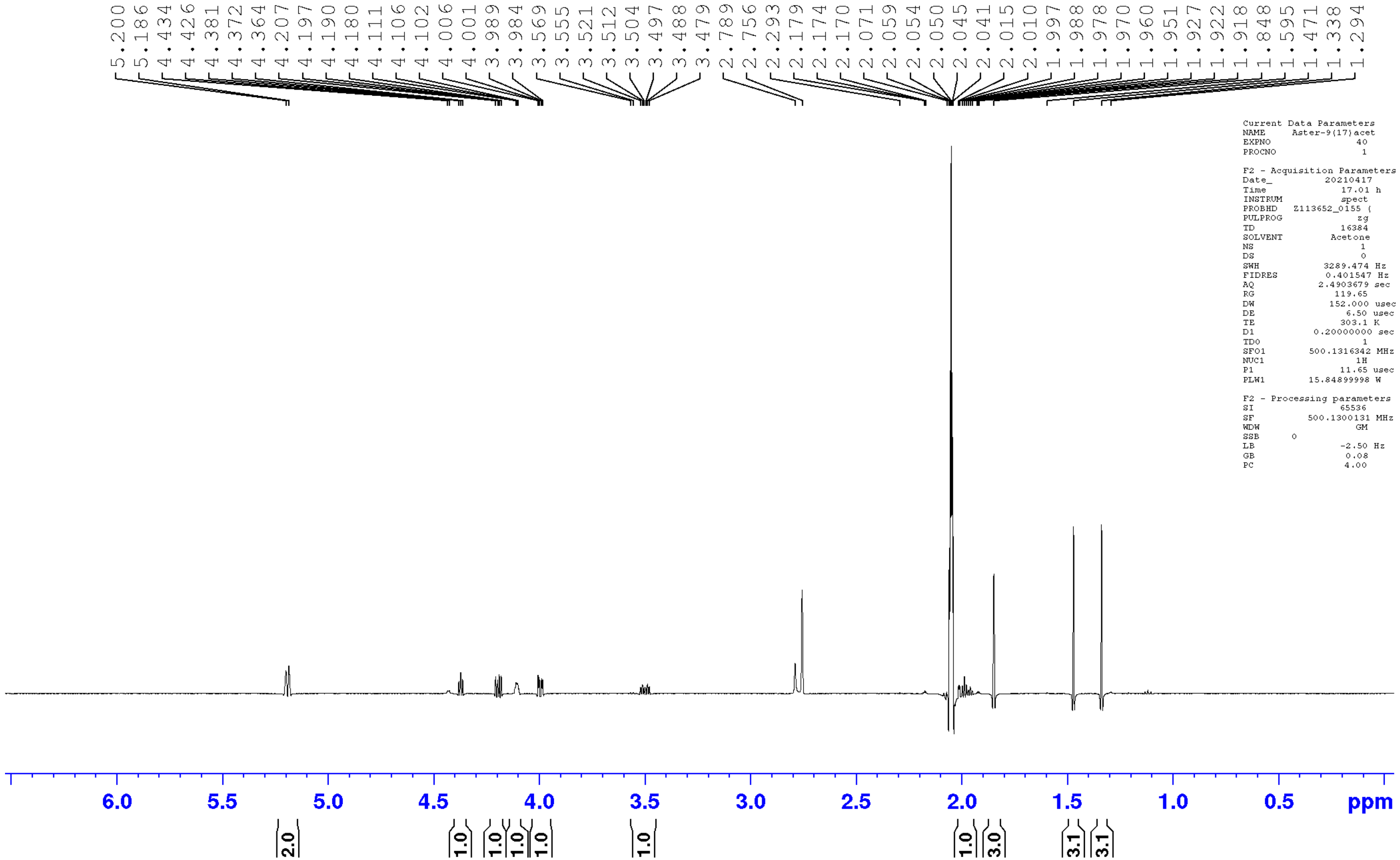

Figure S57. ^{1}H NMR spectrum (500 MHz, acetone-d_6) of **1a**

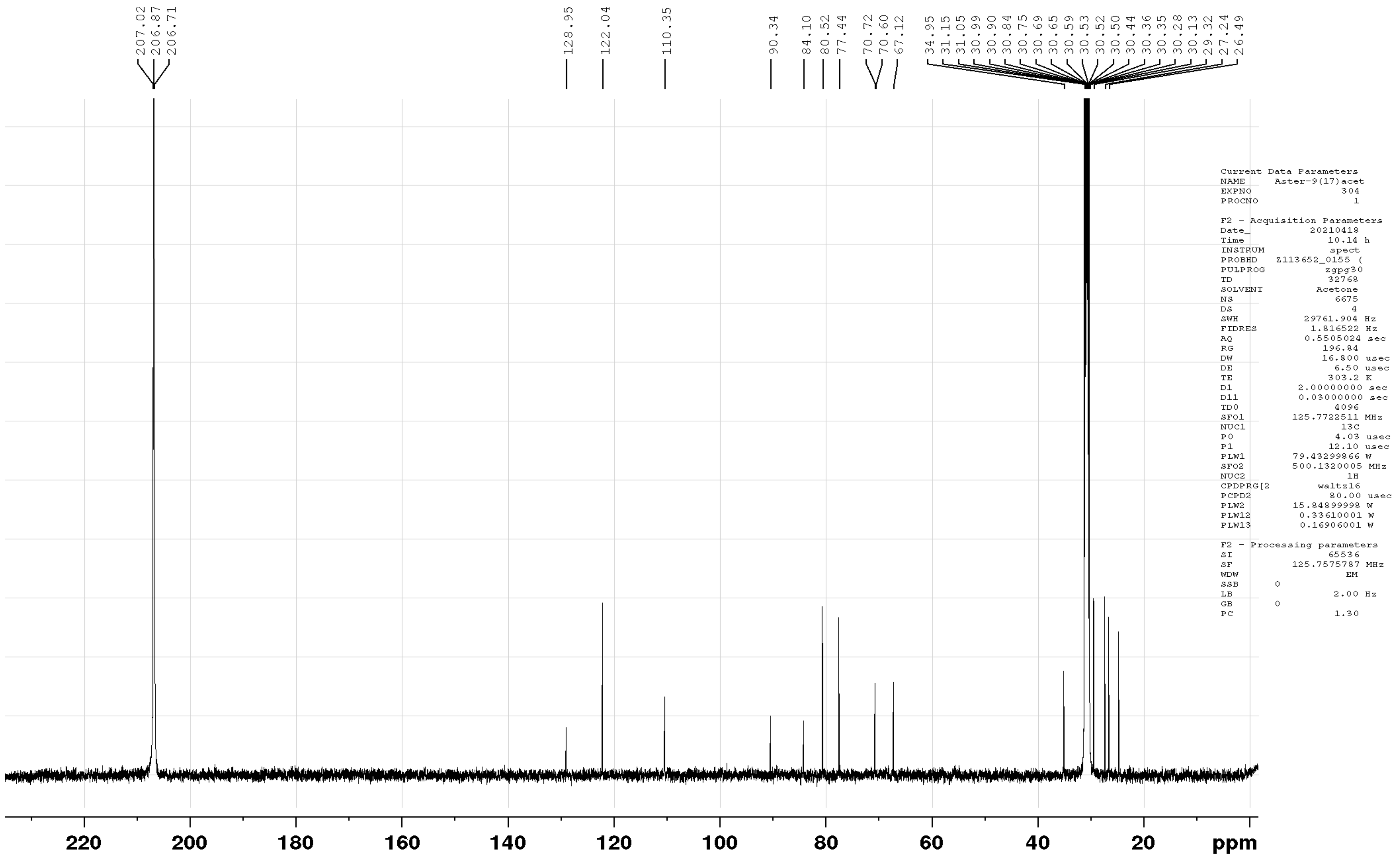

Figure S58. ^{13}C NMR spectrum (125 MHz, acetone-d_6) of **1a**

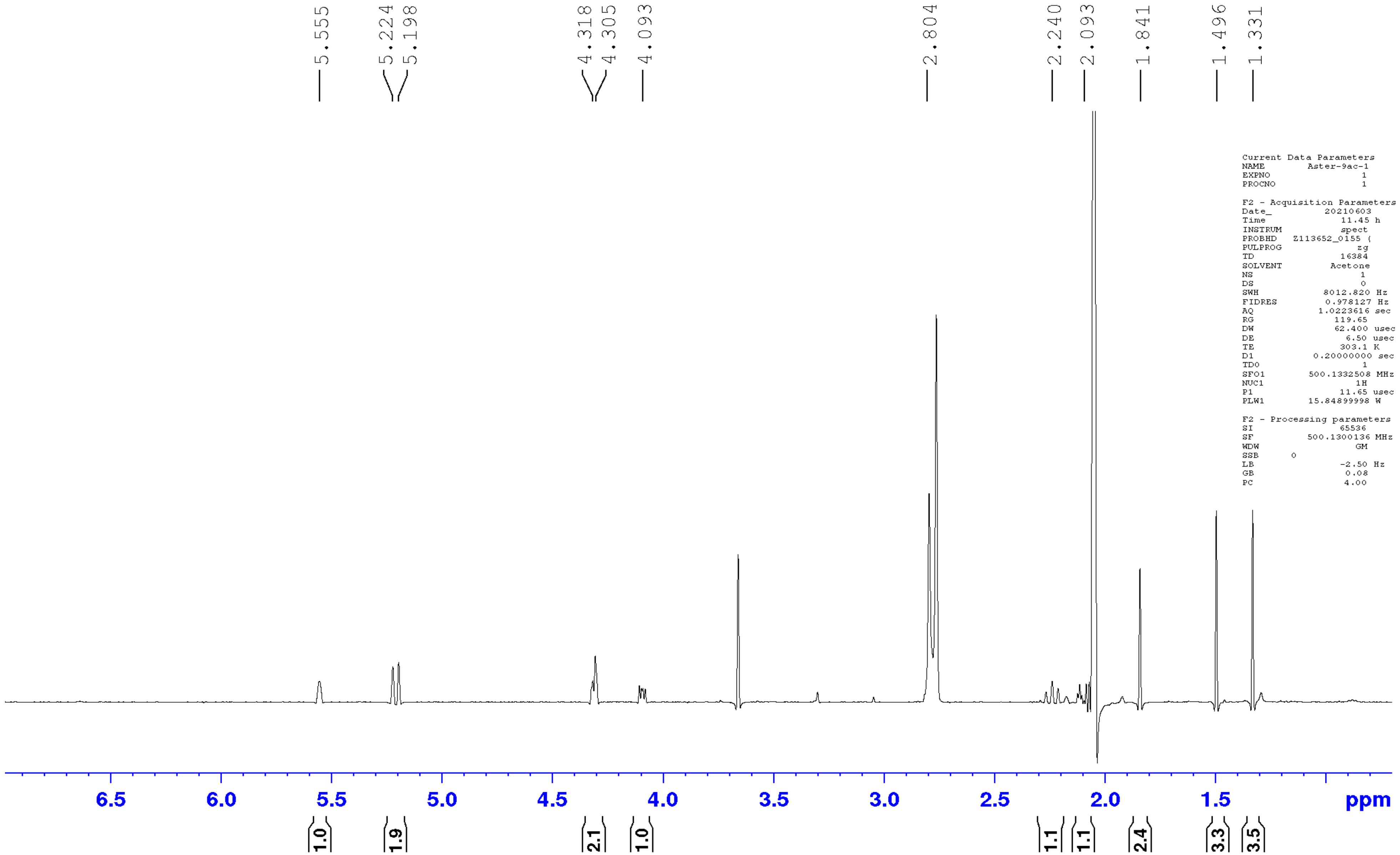

Figure S59. ^{1}H NMR spectrum (500 MHz, acetone-d_6) of **1a-1**

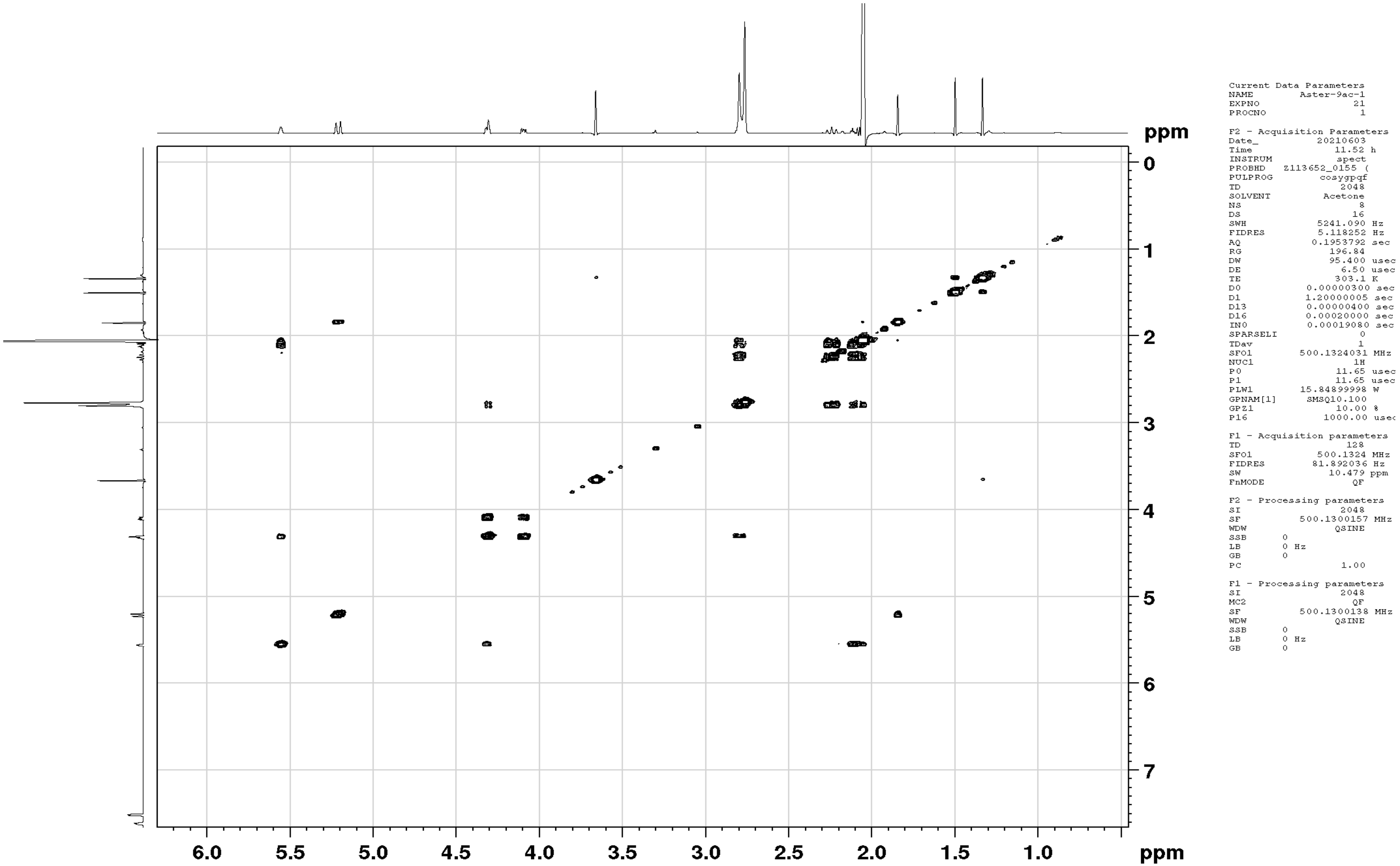

Figure S60. COSY-45 spectrum (500 MHz, acetone-d_6) of **1a-1**

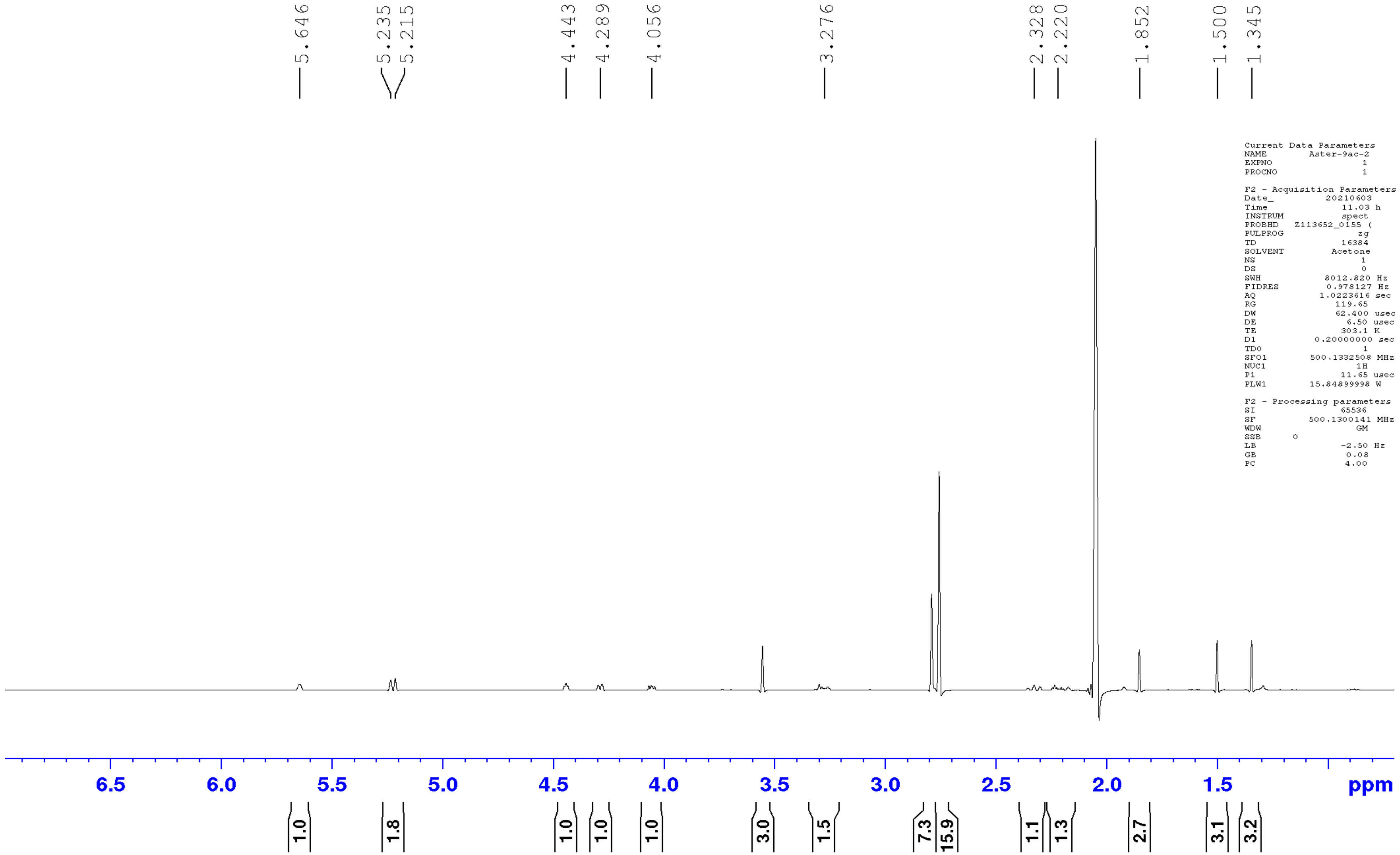

Figure S61. ^{1}H NMR spectrum (500 MHz, acetone-d_6) of **1a-2**

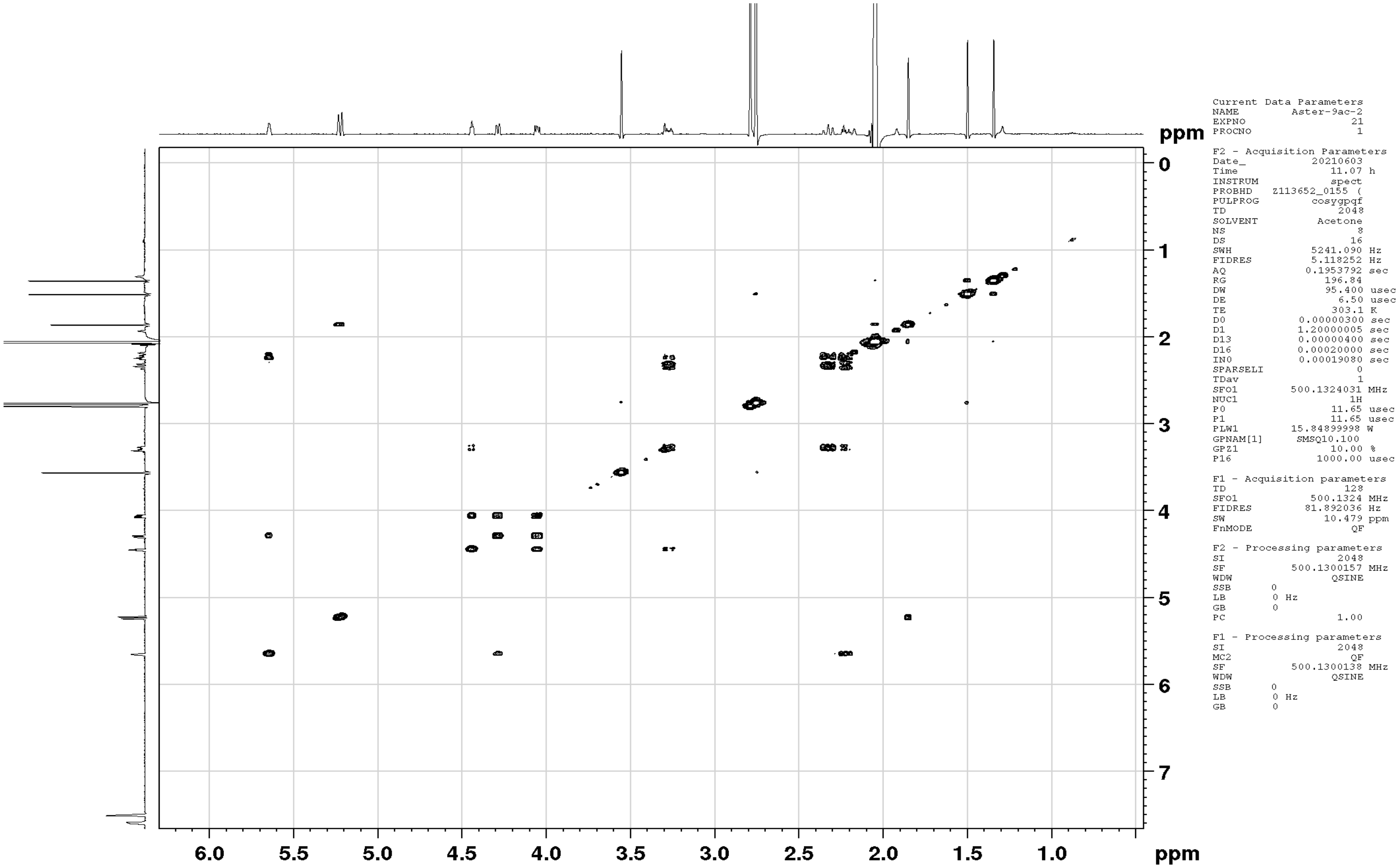

Figure S62. COSY-45 spectrum (500 MHz, acetone-d_6) of **1a-2**

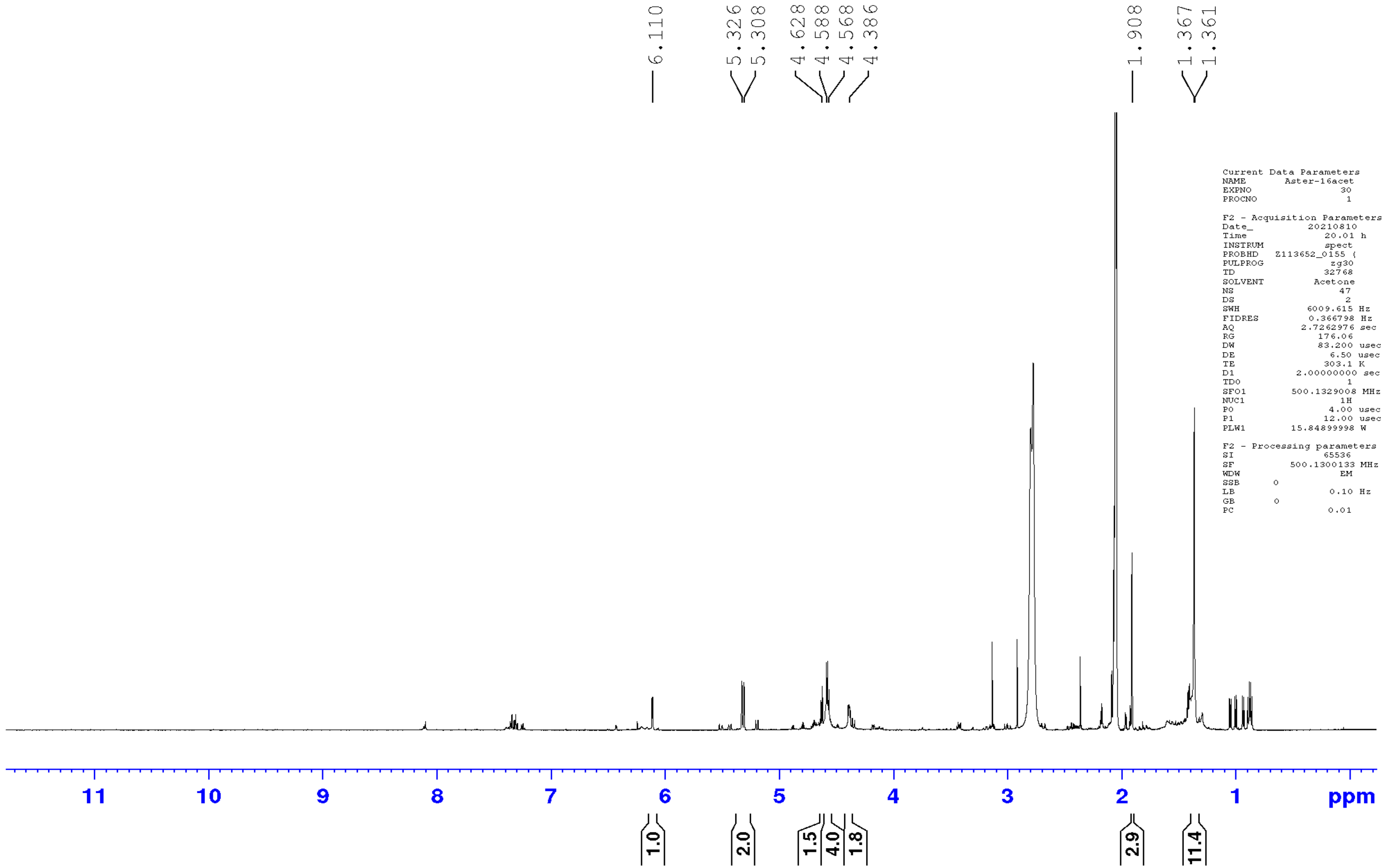

Figure S63. ^{1}H NMR spectrum (700 MHz, acetone-d_6) of **4a**

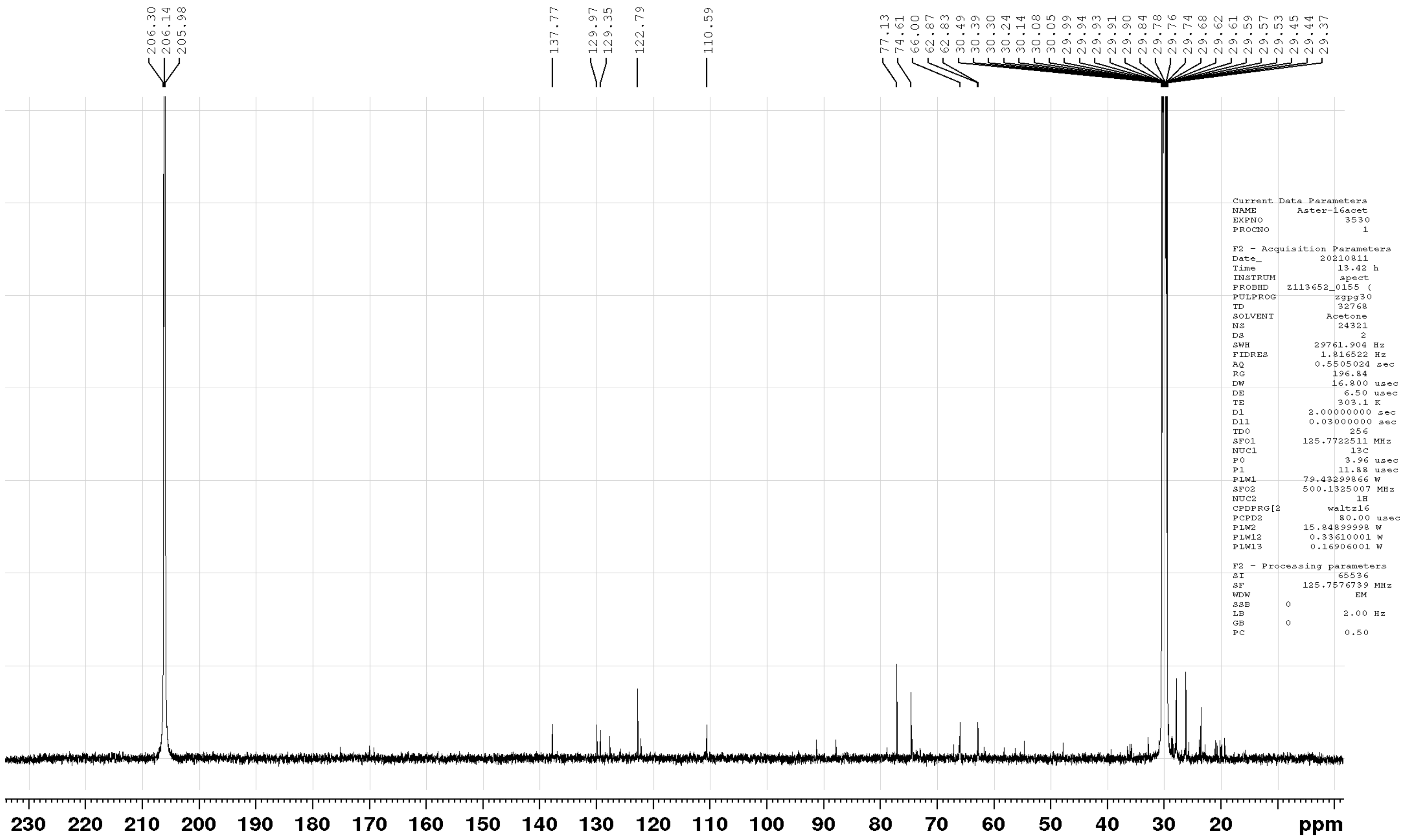

Figure S64. ^{13}C NMR spectrum (125 MHz, acetone-d_6) of **4a**

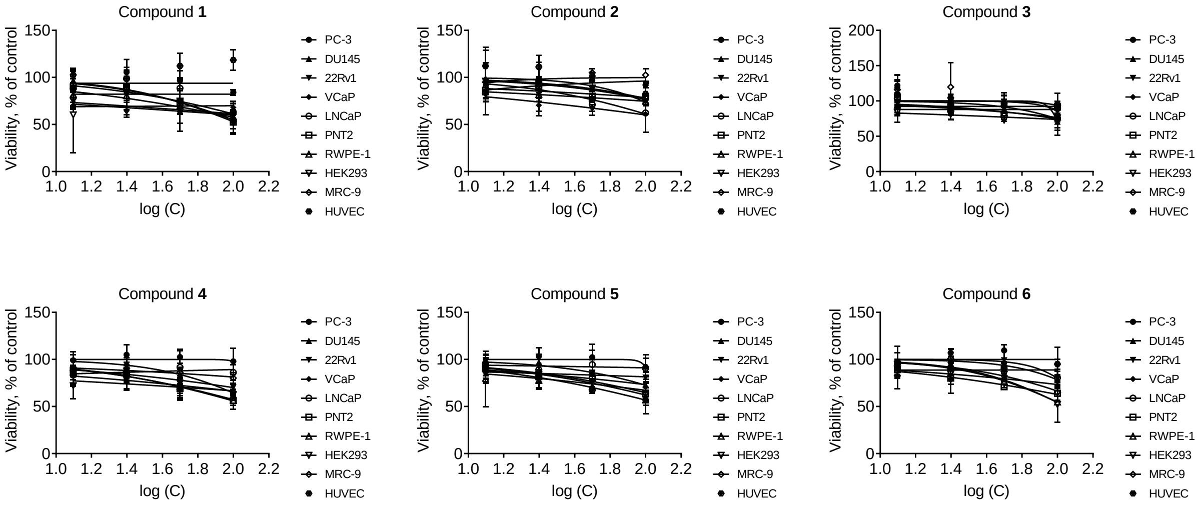

Figure S65. Viability of the cells exposed to the investigated compounds for 72 h. Viability was measured using MTT assay and normalized to control.

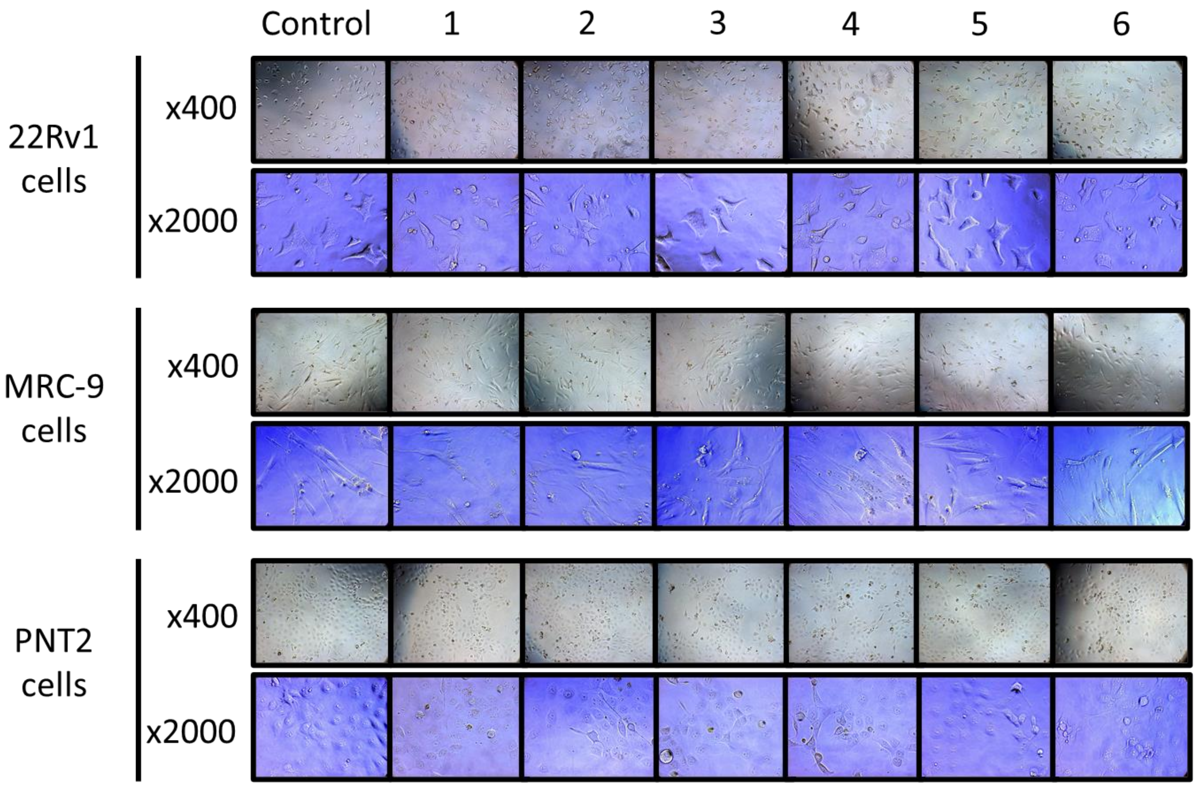

Figure S66. Microphotographies of the cells exposed to the investigated compounds for 72 h. Viability was measured using MTT assay and normalized to control.
